# A structural equation modelling approach to understanding the determinants of childhood vaccination in Nigeria, Uganda and Guinea

**DOI:** 10.1371/journal.pgph.0001289

**Published:** 2023-03-29

**Authors:** James Bell, Belinda Lartey, Marcos Fernandez, Natasha Darrell, Holly Exton-Smith, Cassie Gardner, Emily Richards, Abolaji Akilo, Emmanuel Odongo, James Ssenkungu, Rigobert Kotchi Kouadio, Mamadi Cissé, Axel Bruno Ayiya Igowa Rérambyah, Maikol Adou, Rebecca West, Sunny Sharma

**Affiliations:** 1 Ipsos Healthcare, London, United Kingdom; 2 Ipsos Nigeria, Lagos, Nigeria; 3 Ipsos Uganda, Kampala, Uganda; 4 Ciblage, Dakar, Senegal; 5 Ciblage, Conakry, Guinea; 6 Ciblage, Abidjan, Ivory Coast; 7 Boston University School of Public Health, Boston, Massachusetts, United States of America; University of Michigan, UNITED STATES

## Abstract

Vaccines have contributed to reductions in morbidity and mortality from preventable diseases globally, but low demand for vaccination threatens to reverse these gains. Explorations of the determinants of vaccination uptake may rely on proxy variables to describe complex phenomena and construct models without reference to underlying theories of vaccine demand. This study aimed to use the results of a formative qualitative study (described elsewhere) to construct and test a model to explain the determinants of vaccination uptake. Using the results of a survey among more than 3,000 primary caregivers of young children in Nigeria, Uganda and Guinea, factor analysis produced six explanatory factors. We then estimated the effects of each of these factors on uptake of immunization using a structural equation model. The results showed that the probability that a child is fully vaccinated increases if a caregiver has support from others to vaccinate them (B = 0.33, β = 0.21, p<0.001) and if caregivers had poor experiences with the healthcare system (B = 0.09, β = 0.09, p = 0.007). Conversely, the probability of full vaccination decreases if the caregiver’s husband exerts control over her decision-making ability (B = -0.29, β = -0.20, p<0.001), or if the caregiver perceives vaccines to be of low importance (B = -0.37, β = -0.27, p<0.001). Belief in religious protection (B = -0.07, β = -0.05, p = 0.118) and a belief that vaccines are harmful (B = -0.12, β = -0.04, p = 0.320) did not have an observed effect on vaccination status. This research suggests that interventions may benefit from that including entire families and communities in their design.

## Introduction

Since the establishment of the Expanded Programme on Immunization (EPI) in 1974, vaccinations have contributed to significant reductions in deaths from preventable childhood diseases in low and middle income countries [1]. However in recent years vaccination coverage has plateaued or even decreased in some regions, which jeopardises achieving the Immunization Agenda 2030 goal of reducing mortality and morbidity from vaccine-preventable diseases [2,3]. In the World Health Organisation (WHO) Africa region, for instance, it was estimated that in 2019, 9.4 million children were under- or unvaccinated, which risks epidemics of infectious disease [4].

Low demand for vaccination among caregivers of young children contributes to stagnating coverage rates across Africa [5]. There are various ways to define demand for vaccination, but UNICEF and the World Health Organisation (WHO) describe it as ‘the actions of individuals and communities to seek, support and/ or advocate for vaccines and vaccination services’ [5]. Research on this topic in sub-Saharan Africa proposes that demand for vaccination is informed by family and community priorities and power structures; belief in traditional or religious forms of disease prevention; the exchange of information (including rumours and conspiracy theories) in communities; personal experience of vaccination; and interactions with healthcare systems and providers at the point of delivery [6–18].

The research on vaccine demand to date suggests that many inter-dependent and context-specific factors contribute to uptake of vaccination services [19]. The complexity of the topic is reflected in frameworks such as the 5A or 5C models, which describe the psychological and practical dimensions of vaccination uptake [20,21]. Despite these detailed considerations of vaccination behaviour, quantitative analyses of determinants of demand or uptake of vaccination are rarely based on an underlying theory, may use single variables as proxies for complex and multidimensional factors, and often use statistical models that do not consider the relationships between constructs that drive demand for vaccination in the real world. For example, as Degarege et al. have pointed out, studies of demand for routine vaccination in India typically assume direct relationships between individual sociodemographic, environmental and psychological variables and the outcome in logistic regression models [22]. Research which uses an evidence-based theoretical model of vaccine demand and statistical methods that can account for the multi-faceted determinants of demand and complex relationships between them is required to better understand this topic [23]. Consequently, the aims of this study were to i) propose a theoretical model for vaccination demand based on published literature and formative qualitative research, ii) use data from quantitative surveys of caregivers of young children to test the overall fit of the model to the theory, and iii) understand the comparative importance of predictors of vaccine demand.

The research was conducted in Nigeria, Uganda and Guinea, which were chosen to represent African countries with a range of cultural and religious backgrounds as well as differing vaccination coverage rates. Guinea, a majority Muslim country in West Africa, ranks 182 out of 191 countries on the United Nation’s Human Development Index and faces ongoing political instability following a 2021 coup d’état [24–26]. In 2018, Guinea had the lowest basic vaccination coverage of the three countries (23.9%) as recommended by the Expanded Programme on Immunization (EPI), which are the Bacillus Calmette Guerin vaccine for TB, three doses of DTP-HepB-Hib against diphtheria, tetanus, pertussis, Hepatitis B and Haemophilus influenzae b, three doses of oral polio and one dose of measles [27]. Nigeria, an ethnically and religiously diverse country with relative prosperity in the majority Christian south and higher levels of poverty in the Muslim north, had 31.3% coverage of basic vaccines in 2018 [28–30]. In Uganda, a majority Christian country with one of the world’s highest fertility rates at 5.5 children per women, coverage was reported at 55.2% in 2016 [31,32]. In an analysis of Demographic and Health (DHS) vaccination coverage surveys, Guinea had the lowest percentage of fully vaccinated children of the 25 countries included, Nigeria ranked 22/25 and Uganda 16/25 [33].

## Methods

### Ethics statement

Written informed consent was secured from all participants. An honorarium was provided in the form of a small household item in Nigeria (approximate value of 1000 NGN/ 2.40 USD) and Uganda (5,300 UGX/ 1.50 USD) and in cash in Guinea (369,000 FG/ 40 USD). The incentive amounts varied across the three countries due to local research norms. The study protocol received approval from Makerere University College of Health Sciences Review Board in Uganda (Ref: 724), the National Health Research Ethics Committee of Nigeria (Approval number: NHREC/01/01/2007-25/09/2019) and the Comité Nationale d’Ethique pour la Recherche en Santé in Guinea (Ref: 026/CNERS/20). Additional information regarding the ethical, cultural, and scientific considerations specific to inclusivity in global research is included in the Supporting Information (S1 Checklist)

### Data collection

Data were collected using a questionnaire (S2 Text), designed using the results of a formative qualitative study [19] and a literature review. The qualitative work found that family relationships, social connections, attitudes to healthcare and governmental institutions, and interaction with social and cultural norms around health and child development informed vaccination behaviour. These themes informed the contents of the questionnaire, which collected demographic data, household income, and the vaccination status of the participant’s child, as well as perceptual information on their family and community relationships, traditional and religious beliefs, methods of child protection and attitudes to vaccination and vaccination services. The survey contained attitude statements on these topics, to which participants indicated their agreement or disagreement using a 5-point Likert scale. The questionnaire was translated into Yoruba, Hausa and Igbo in Nigeria; Luganda, Runyankole, Samia, Japadhola and Acholi in Uganda; and French in Guinea, so that enumerators could interpret the questions into Malinké, Soussou or Peul, as required. The survey was administered by trained enumerators using Computer Assisted Personal Interviewing (CAPI) devices. Enumerators were trained over the course of four days in each country.

The research was conducted in six states in Nigeria (Lagos, Kano, Enugu, Sokoto, Nasarawa and Rivers), five regions in Uganda (Acholi, Bukedi, Kampala, North Central and Ankole) and five regions in Guinea (Boké, Conakry, N’Zérékoré, Mamou and Kankan). The regions were selected non-randomly with in-country stakeholders (including EPI and government representatives) to include a range of cultural groups and vaccination coverage rates. As the study did not aim to detect an effect of a specific size, the sample size of 1000 per country was chosen in consultation with stakeholders to ensure they had sufficient confidence in the results at a regional or state level.

A multi-stage, stratified sampling methodology was used in each of the regions to select households for interview. Details are given in the (S1 Text). The exact procedure varied by country, but was made as consistent as possible to avoid introduction of bias. In general, the sample was stratified by urban or rural setting within each region. Lower-level geographic areas were selected within each stratum and a starting point determined. Households were then selected following a random walk procedure, a household census was taken, and eligible respondents were selected (using a Kish grid if more than one was present). The sampling protocol was revised after ethical approval in Guinea because of safety concerns due to flooding in some regions (see (S1 Text)). Participants were eligible if they had primary responsibility for the care of a child between 1 and 3 years old. Both male and female participants were eligible for inclusion. Surveys were administered in private where possible to reduce social desirability bias.

The data were continuously checked for completeness and quality during the fieldwork period and follow-ups were initiated as needed. This meant that the final file was complete and without any missing data. Identifying information such as participant names and telephone numbers were removed before analysis.

### Analysis

Analyses were carried out in R v.4.0.2 using the psych and lavaan packages [34,35]. The data and analysis scripts are available in a Github repository [link: https://github.com/jamesbell1991/Vaccines_Structural_Equation_Modelling].

First, the demographics of the study sample were described. Monthly household income was divided into three bands. Low: Nigeria (Below 50,000 NGN), Uganda (Below 500,000 UGX), Guinea (Below 1,983,626 GNF); Middle: Nigeria (50,0001–500,000 NGN), Uganda (501,000–2,000,000 UGX), Guinea (1,983,627–4,999,999 GNF); High: Nigeria (Above 800,0001 NGN), Uganda (Above 2,000,000 UGX), Guinea (Above 5,000,000 GNF). These ranges were decided in consultation with local research teams based on survey norms and statistical reports in each country.

The structural equation modelling process broadly followed the protocol detailed by Schumacker and Lomax [36]. Firstly, a factor analysis was conducted on several Likert-scale questions in the survey. The approach was a combination of exploratory analysis (in that no definite factor structure was predetermined) and confirmatory analysis (in that variables were grouped together in themes in advance of the analysis) as described by Kang et al [37]. The final factors were determined through an examination of scree plots and factor loadings to produce six factors. Variables with a factor loading less than 0.3 were considered a poor fit.

The composition of these factors and the theoretical basis for including them was arrived at using the results of a literature review and previous qualitative research (Table 1).

**Table 1:** Factor structure and their theoretical justifications.

Factor	Component variables	Rationale for inclusion
Belief in religious protection	• My religious faith protects me and my family from harm • My religious faith heals me and my family from illnesses • God is the only protection needed against harm • My religious faith guides decisions in my life	The vaccine demand literature suggests that religious belief could play a part in reducing demand for childhood vaccination [38–40]. Our qualitative study, however, concludes that religious belief has little direct bearing on uptake of vaccination, but that the gender norms Christianity and Islam uphold may reduce a caregiver’s capability to seek vaccination in more circuitous ways [19].
Control of husband over decisions	• When a man makes a decision, no one in the family should question it • Disagreements between a husband and wife should not be talked about outside of the home • [Wording in Nigeria and Uganda] A man should watch over his wife to make sure she does the right things; [Wording in Guinea] A man should monitor his wife to make sure she does the right things • I am worried about being blamed if I make a decision for my baby/child and something goes wrong	Previous studies have concluded that the influence of a caregiver’s husband is important in encouraging or discouraging vaccination seeking [9,16]. Our qualitative study reinforced this finding [19].
Support for vaccination from others	• [Wording in Nigeria and Uganda] My husband/ partner helped/ ensured that may child was vaccinated; [Wording in Guinea: My spouse/ partner helped/ ensured that may child was vaccinated] • My mother/ mother-in-law helped ensured that my child was vaccinated • It is normal in this community to vaccinate your children • Religious leaders are supportive of vaccination • I trust that the government knows what is right for children	Building norms around vaccination is understood to be important, as are the support of family members and religious leaders and trust in government and public institutions [7,8,10,12,41–43]. Our qualitative study found that family, friends and neighbours were important in setting vaccination norms, and that low trust in institutions contributed to suspicion of vaccines [19].
Belief that vaccinations are not important/ necessary	• I travel a lot so it’s hard to take my children to get vaccinated • I am too busy to go to the clinic for vaccinations • There are no benefits to vaccination • Children who have not had vaccinations are usually healthy • There are other ways I can protect my child from disease	Lack of awareness and understanding of immunization and the disease they prevent is understood as a foundational barrier to increasing demand for vaccinations [9]. Parents may not always view vaccines as necessary if they do not perceive vaccine-preventable diseases as a threat [44]. Parents may have conflicting priorities, which reduce the likelihood that a child will be vaccinated [9,19,43,45,46]. Communities may also have other ways to protect children which are more culturally embedded [9,19,47].
Poor service delivery experience	• The staff in the hospital are rude to me • The clinic or hospital is dirty • The queues are too long at the clinic/hospital where the vaccination takes place	Literature on vaccination demand, including our qualitative study, has consistently shown that poor experiences of the healthcare system contribute to low vaccination uptake [7–10,12,14,19,41,48].
Belief that vaccines are harmful	• Having many vaccinations at once is hard for children to bear • It is difficult for me to manage the side effects (fever, rash, pain) of vaccination • Vaccines are a way for global/western countries/ organisations to control us	Side-effects are a commonly cited concern about vaccinations among caregivers [7]. Vaccination rumours have also been shown to contribute to low uptake [7,9,15,19,42,45,46,49–51]. There is also some evidence that caregivers may believe too many vaccines are administered at once [7].

Using these six factors, a structural equation model structure was developed with children’s vaccination status as the dependent/outcome variable. Vaccination status was determined using an adapted version of the protocol used by DHS [52]. If available, the vaccines a child had received were determined using the child’s vaccination card. If not available, status was determined by parental reporting, which was not otherwise verified (e.g., through clinic records). For the analysis a dichotomous variable was created to compare children who have completed the full schedule (taking a value of 2) or who have had no doses or some doses but not enough to complete the full schedule (taking a value of 1).

**Fig 1:**
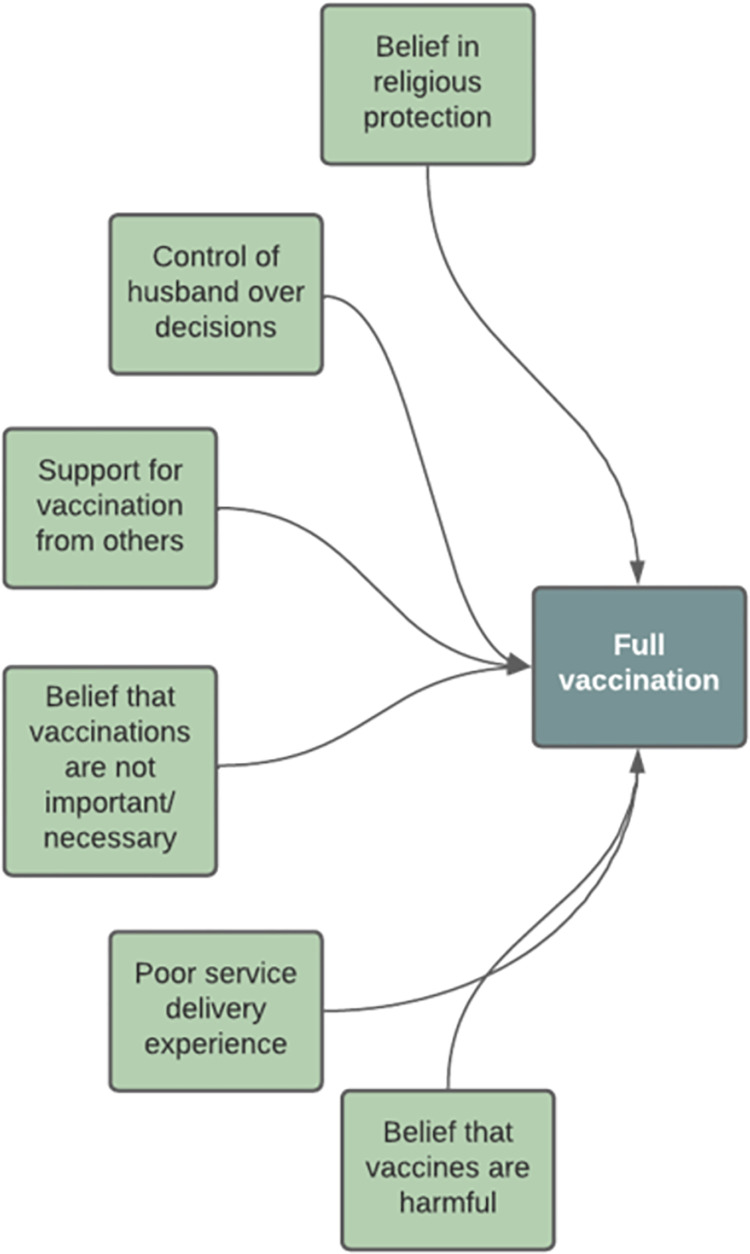
Proposed model structure.

As shown in Fig 1, it was hypothesized that each factor had a direct relationship with the outcome. Existing literature and our previous qualitative study do not support any hypothesized relationships between the factors.

The model used a probit link function, to account for the dichotomous outcome variable. "Don’t know” or “refused” responses were recoded to the neutral point of the Likert scale, and Likert scales were considered continuous in the factor analysis. Modification indices were examined and additions or deletions to the model were considered. Several goodness of fit indices were examined. No definitive cut-off points were adopted, but the guidance that RMSEA <0.08, TLI >0.90, CFI >0.90 and SRMR <0.08 indicate acceptable fit was used [53]. A Χ^2^ test was not included due to its sensitivity to sample size [36]. Finally, as the countries involved in the study may be heterogenous, models were run for each country separately, the results of which are given in the (S3 Text).

All tables and figures presented contain sample statistics and have not been weighted to population data. Sample clustering was not taken into account in the structural equation models.

## Results

### Description of study participants

A total of 3,318 interviews were completed. These took place in Nigeria and Uganda between November and December 2020 and in Guinea between July and August 2021 (later due to resource constraints which prevented the three surveys from running concurrently). Just under a third of interviews were conducted in rural areas (Table 2). Most participants (78.8%) were under the age of 35. Education levels varied by country: in Nigeria, 56.5% of participants had secondary or higher education, whereas in Uganda most participants had primary education (55.1%). In Guinea, 52.2% of participants had no formal education, the highest of the three countries. In all countries most participants were in the low-income band. 96.0% of participants were the child’s biological mother. Vaccination status of the sample varied by country, with Uganda reporting 60.4% of children fully vaccinated, and lower percentages in Nigeria and Guinea (36.1% and 40.0%, respectively).

**Table 2:** Description of study sample.

	Nigeria (N = 1264)	Uganda (N = 1054)	Guinea (N = 1000)	Total (N = 3318)
**Setting**				
Urban	489 (38.7%)	406 (38.5%)	363 (36.3%)	1258 (37.9%)
Rural	775 (61.3%)	648 (61.5%)	637 (63.7%)	2060 (62.1%)
**Age**				
18–24	261 (20.6%)	358 (34.0%)	262 (26.2%)	881 (26.6%)
25–29	406 (32.1%)	312 (29.6%)	340 (34.0%)	1058 (31.9%)
30–34	268 (21.2%)	191 (18.1%)	214 (21.4%)	673 (20.3%)
35–39	216 (17.1%)	124 (11.8%)	131 (13.1%)	471 (14.2%)
40–44	83 (6.6%)	48 (4.6%)	33 (3.3%)	164 (4.9%)
45–49	26 (2.1%)	15 (1.4%)	16 (1.6%)	57 (1.7%)
50–56	4 (0.3%)	6 (0.6%)	4 (0.4%)	14 (0.4%)
**Education**				
No formal education	387 (30.6%)	74 (7.0%)	522 (52.2%)	983 (29.6%)
Primary	163 (12.9%)	581 (55.1%)	185 (18.5%)	929 (28.0%)
Secondary	530 (41.9%)	319 (30.3%)	188 (18.8%)	1037 (31.3%)
Higher education	184 (14.6%)	79 (7.5%)	77 (7.7%)	340 (10.2%)
Prefer not to answer	0 (0%)	1 (0.1%)	28 (2.8%)	29 (0.9%)
**Income level**				
Low	730 (57.8%)	759 (72.0%)	543 (54.3%)	2032 (61.2%)
Middle	306 (24.2%)	228 (21.6%)	115 (11.5%)	649 (19.6%)
High	131 (10.4%)	24 (2.3%)	2 (0.2%)	157 (4.7%)
Prefer not to say	97 (7.7%)	43 (4.1%)	340 (34.0%)	480 (14.5%)
**Number of children**				
Mean (SD)	2.92 (1.89)	3.35 (2.29)	3.69 (2.18)	3.29 (2.13)
Median [Min, Max]	3.00 [1.00, 20.0]	3.00 [1.00, 20.0]	3.00 [1.00, 16.0]	3.00 [1.00, 20.0]
**Relationship to child**				
Biological mother	1195 (94.5%)	1038 (98.5%)	953 (95.3%)	3186 (96.0%)
Stepmother	18 (1.4%)	4 (0.4%)	3 (0.3%)	25 (0.8%)
Aunt	40 (3.2%)	7 (0.7%)	16 (1.6%)	63 (1.9%)
Grandmother	3 (0.2%)	4 (0.4%)	0 (0%)	7 (0.2%)
Biological father	7 (0.6%)	1 (0.1%)	4 (0.4%)	12 (0.4%)
Stepfather	1 (0.1%)	0 (0%)	2 (0.2%)	3 (0.1%)
Other	0 (0%)	0 (0%)	22 (2.2%)	22 (0.7%)
**Child’s vaccination status**				
Not vaccinated	143 (11.3%)	87 (8.3%)	106 (10.6%)	336 (10.1%)
Partially vaccinated	665 (52.6%)	330 (31.3%)	494 (49.4%)	1489 (44.9%)
Fully vaccinated	456 (36.1%)	637 (60.4%)	400 (40.0%)	1493 (45.0%)

### Measurement model

The measurement model corresponding to the six latent factors in Table 1 fitted the data reasonably well (RMSEA = 0.04, TLI = 0.88, CFI = 0.89, SRMR = 0.04). To improve the fit further, we allowed some residual terms within the same construct to covary (My spouse/ partner helped/ ensured that my child was vaccinated with My mother/ mother-in-law helped/ ensured that my child was vaccinated and It is normal in this community to vaccinate your children with Religious leaders are supportive of vaccination), resulting in the final measurement model (Table 3). One variable (Disagreements between a husband and wife are private and should not be talked about outside the home) had a standardised factor loading of 0.294 but was retained in the model as its removal did not appreciably improve the fit statistics.

**Table 3:** Measurement model factor loadings and fit statistics.

Factor	Variable	Factor loading	Standard error	P-value	Standardised factor loading
**Belief in religious protection**	My religious faith protects me and my family from harm	1.000			0.755
	My religious faith heals me and my family from illnesses	1.196	0.037	<0.001	0.708
	God is the only protection needed against harm	0.617	0.023	<0.001	0.553
	My religious faith guides decisions in my life	0.710	0.024	<0.001	0.612
**Control of husband over decisions**	When a man makes a decision, no one in the family should question it	1.000			0.498
	[Wording in Nigeria and Uganda] A man should watch over his wife to make sure she does the right things; [Wording in Guinea] A man should monitor his wife to make sure she does the right things	0.827	0.059	<0.001	0.522
	Disagreements between a husband and wife are private and should not be talked about outside the home	0.474	0.045	<0.001	0.294
	I am worried about being blamed if I make a decision for my baby/ child and something goes wrong	0.542	0.050	<0.001	0.301
**Support for vaccination from others**	[Wording in Nigeria and Uganda] My husband/ partner helped/ ensured that may child was vaccinated; [Wording in Guinea: My spouse/ partner helped/ ensured that may child was vaccinated]	1.000			0.542
	My mother/ mother-in-law helped/ ensured that my child was vaccinated	1.045	0.045	<0.001	0.512
	It is normal in this community to vaccinate your children	0.778	0.037	<0.001	0.612
	Religious leaders are supportive of vaccination	0.747	0.040	<0.001	0.485
	I trust that the government knows what is right for children	0.891	0.045	<0.001	0.541
**Belief that vaccinations are not important/ necessary**	I travel a lot so it’s hard to take my child to get vaccinated	1.000			0.528
	I am too busy to go to the clinic or hospital for vaccinations	1.115	0.047	<0.001	0.528
	There are no benefits to vaccination	1.123	0.058	<0.001	0.561
	Children who have not had vaccinations are usually healthy	1.059	0.058	<0.001	0.497
	There are other ways I can protect my child from disease	0.732	0.052	<0.001	0.334
**Poor service delivery experience**	The staff in the hospital are rude to me	1.000			0.612
	The clinic or hospital is dirty	1.029	0.058	<0.001	0.664
	The queues are too long at the clinic/ hospital where the vaccination takes place	0.455	0.035	<0.001	0.309
**Belief that vaccines are harmful**	Having many vaccinations at once is hard for children to bear	1.00			0.356
	It is difficult for me to manage the side effects (fever, rash, pain) of vaccination	1.843	0.139	<0.001	0.586
	Vaccines are a way for global/western countries/organisations to control us	1.682	0.128	<0.001	0.530

RMSEA = 0.04, TLI = 0.90, CFI = 0.91, SRMR = 0.04.

### Structural model

The fit statistics for the model indicate acceptable model fit: RMSEA = 0.04, TLI = 0.91, CFI = 0.92, SRMR = 0.04. Modification indices were examined, but none were logical within the theoretical framework so none were adopted.

### Factors affecting uptake of childhood vaccination

Some factors are associated with a reduction in the probability that a child would be vaccinated, while others lead to an observed increase in the probability of vaccination, and others were unassociated with the outcome (Table 4).

**Table 4:** Unstandardised (B) and standardised (β) effects of factors affecting vaccination in the structural model.

Factor	B (95% CI)	β (95% CI)	P-value
Belief in religious protection	-0.07 (-0.17, 0.02)	-0.05 (-0.11, 0.01)	0.118
Control of husband over decisions	-0.29 (-0.43, -0.14)	-0.20 (-0.29, -0.11)	<0.001
Support for vaccination from others	0.33 (0.19, 0.46)	0.21 (0.13, 0.30)	<0.001
Belief that vaccinations are not important/ necessary	-0.37 (-0.51, -0.22)	-0.27 (-0.37, -0.17)	<0.001
Poor service delivery experience	0.09 (0.02, 0.16)	0.09 (0.03, 0.16)	0.007
Belief that vaccines are harmful	-0.12 (-0.37, 0.12)	-0.04 (-0.13, 0.04)	0.320

Lower probability of vaccination was observed for those who expressed higher levels of perceived control of the husband over decision-making (B -unstandardised effect = -0.29, β- standardised effect = -0.20, p<0.001). The unstandardised effect can be interpreted to mean that when this variable increases by one unit, the z-score for probability of being fully vaccinated decreases by 0.29 units. The standardised coefficient can be interpreted to mean that when this variable is increased by one standard deviation, the z-score score for probability of being fully vaccinated decreases by 0.20 standard deviations. Lower probability was also observed for those who expressed higher levels of belief that vaccinations are not important or necessary (B = -0.37, β = 0.27, p<0.001). Higher probabilities of vaccination were observed for participants who said that they had higher levels of support for vaccination from others around them (B = 0.33, β = 0.21, p<0.001) and among those who had worse service delivery experiences (B = 0.09, β = 0.09, p = 0.007). There was little evidence that belief in religious protection (B = -0.07, β = -0.05, p = 0.118) or belief that vaccines are harmful (B = -0.12, β = -0.04, p = 0.320) increased or decreased the probability of vaccination.

In a comparison of the standardised coefficients (β), the factor with the strongest positive observed impact on vaccination was having support from others to vaccinate. The strongest negative impacts were observed for those who expressed high degrees of control of decisions by the husband, and stronger beliefs that vaccinations were not important or necessary.

## Discussion

This study used structural equation modelling to examine factors associated with uptake of childhood vaccination among primary caregivers in Uganda, Guinea and Nigeria. The results suggest that vaccination uptake is informed by family and community relationships, service delivery experience and attitudes and beliefs towards vaccination. Elements of the findings were consistent with existing research on this topic. Higher levels of spousal control over decision-making were again linked to lower likelihood to vaccinate, the role of community norms in encouraging vaccination was reaffirmed, and the importance of belief in the necessity of vaccines in the context of other priorities was observed [9,10,12]. The determinants of vaccination acceptance we identified also overlapped with established frameworks such as the 5A and 5C models [20,21].

The study provides new contributions to our understanding of the determinants of vaccine demand in several ways. Thematically, the study gives alternative perspectives on the role of religious belief and healthcare service experience compared to what is prevalent in the literature. Conceptually, the work departs from standard methodologies employed in vaccine demand research by using analytical approaches that account for the complexity of the factors that inform vaccine uptake, and which are based on underlying data-driven theories of behaviour.

Given what is reported elsewhere in the literature, two of the study’s conclusions may appear surprising. Others have suggested that caregiver belief in religious protection may decrease likelihood of vaccine uptake [38–40]. Our findings do not support this hypothesis, which is in line with the results of our qualitative research on the same topic [19]. It is possible that religious protection and protection conferred by vaccines are seen as conceptually separate, and with different functions in child development. This means that interventions to increase demand for vaccination should be careful not to attempt to supplant belief in religious protection with a preference for vaccination. Interventions involving religious community leaders (such as have been attempted in Nigeria) could be fruitful avenues to ensure that different conceptions of child protection are viewed as complementary rather than adversarial [54–56].

It is well established that poor service delivery experiences may discourage caregivers from seeking vaccination [7–9,12–14,19]. Even though the effect size observed in our study was small, it is surprising that our results suggest that caregivers who experience worse service delivery experience are more likely to have fully vaccinated children. There are several possible explanations for this finding. In the country-level analysis (presented in the S3 Text) the association is driven by the data from Guinea, which suggests that the finding may be due to sampling or cognitive biases in questionnaire responses that are specific to that country. Informal conversations with the fieldwork teams revealed that participants were at times unwilling to give negative opinions about the government, which may have affected responses to the variables comprising this factor. Alternatively, it is theoretically plausible that those who had fully vaccinated children are more dissatisfied with the experience of vaccinating at the clinic, compared to those with un- or under-vaccinated children, who will have had fewer touchpoints with health services. Finally, the result could have been the result of uncontrolled confounding by variables that were not included in the model.

Our study’s results also support the idea that vaccination uptake is not determined solely by the attitudes and behaviours of the child’s primary caregiver, but by a range of intersecting familial, community and social influences. This suggests that ‘whole family’ or ‘whole community’ intervention approaches could be impactful in these contexts. Programmes based on principles of collectivism encourage families and communities to adopt a desired behaviour together, and have shown promise in other policy areas and geographies [57,58].

When the analysis is done separately by country, some differences by geography are noted. In Nigeria, support from others is observed to drive vaccination uptake, and bad service delivery impedes it. In Uganda, practical difficulties are the sole barrier to uptake, and in Guinea support from others, bad service delivery and belief in religious protection increase the probability of vaccination and belief that vaccinations are harmful decreases it. These differences mean that interventions should ensure that local contexts are taken into account when designing strategies to encourage adoption of vaccination.

This study moved beyond the standard approach in many explorations of predictors of childhood vaccination demand, which may rely on observed variables only as model inputs. Determinants of demand are often multifaceted in nature, necessitating the use of latent variables or constructs [23]. In this way, our study was able to engage with the complexity of the phenomenon more holistically in its analytical approach. In addition, our analysis was also explicitly based on themes identified through prior qualitative research. A research-based approach, and the choice of structural equation modelling as the analytical tool, ensured that the hypothesised relationships between the explanatory factors had an empirical basis and were stated explicitly rather than assumed. This may result in models that reflect more closely how decisions around vaccination play out in the real world, which may make resulting interventions more appropriate.

Further research on this topic could undertake more complex analysis than has been attempted here. This could include developing factors to describe other important constructs that may affect vaccination (such socio-economic status or belief in gender norms), proposing and testing more elaborate relational structures between factors, or the exploration of potential moderation or mediation between latent constructs.

### Limitations

Some important limitations should be considered when evaluating the research findings. All answers were self-reported and not verified using external sources, so the vaccination outcome data may have been over- or under-stated. Attitudinal questions may have been affected by social desirability or recall biases. The sampling methodology should have resulted in regionally representative samples, but the random-walk methodology could have introduced sampling bias [59]. The differences between the sampling protocols (as explained the Supplementary Materials) could also reduce comparability between countries.

The factors included in the model were partially determined by the availability of data, and therefore important constructs are likely absent from the analysis, rendering it an incomplete view of the determinants of vaccination uptake. Structural equation modelling makes stronger linearity assumptions compared to traditional regression analyses, and assumes that effect estimates are unconfounded, which complicates the interpretation and application of our findings [60]. In addition, some responses to the Likert scales that comprised the factors were skewed, which could affect the interpretation of the results.

Finally, the standardised factor loading scores are considered low by many measures, meaning that the cohesiveness of the latent constructs and the regressions based on them are open to critique [61]. The decision to combine data from three heterogeneous countries is also open to criticism as it may obscure country-level dynamics (but this is remedied by the inclusion of country-level models in the S3 Text).

## Conclusion

Research on vaccination uptake often relies on proxy variables to represent complex phenomena and may not be based on an underlying theory of how vaccination decisions are made. This article uses the results of a formative qualitative study to construct and test a model to help explain determinants of vaccination uptake. We conclude that uptake is informed by family and community relationships, service delivery experience and attitudes and beliefs towards vaccination. The work has implications for intervention design and suggests that approaches that include entire families and communities in interventions may be beneficial.

## Supporting information

S1 Checklist Inclusivity in global research. (DOCX)

S1 Text Sampling protocols. (DOCX)

S2 Text Questionnaires. (DOCX)

S3 Text Country analysis. (DOCX)
