# Supplementary material for: A structural equation modelling approach to understanding the determinants of childhood vaccination in Nigeria, Uganda and Guinea
Source: PLOS Glob Public Health. 2023 Mar 29;3(3):e0001289. doi: 10.1371/journal.pgph.0001289 (PMC10058155; doi:10.1371/journal.pgph.0001289)
Supplement: S1 Text — (DOCX) [file pgph.0001289.s002.docx]

**A Structural Equation Modelling Approach to Understanding the Determinants of Childhood Vaccination in Nigeria, Uganda and Guinea**

**Supplementary Material 1**

**Sampling Protocols**

[1. Nigeria Sampling Protocol 2](#_Toc111663095)

[2. Uganda Sampling Protocol 14](#_Toc111663096)

[3. Guinea Sampling Protocol 23](#_Toc111663097)

# Nigeria Sampling Protocol

## Sample Size

1,000 interviews with caregivers

## Sample Definition

- Must have at least one child
- Must reside in one of the study sites
- No restrictions in terms of age, income, level of education or ethnic background.
- Must have primary caring responsibilities for a child between the age of 1 and 3 years
  - This is to ensure that we only interview caregivers of children who have had the opportunity to have all recommended vaccines in the 0-12 months age range. We have restricted the age to maximum 3 years so that we do not rely on recall that is too distant.
  - If participants have multiple eligible children, the interview will focus on the youngest eligible child.
  - If the youngest are twins/ triplets etc., a child will be randomly selected using a Kish grid

## Sample allocation requirements

- The sample for all respondent categories will be equally distributed across the selected states (Sokoto, Kano, Nasarawa, Lagos, Rivers and Enugu)
- The rural/urban allocation will be as per the national statistics of each state
- Number of wards per sampled local government area (LGA) will be determined by the number of interviews allocated to the LGA. This will be done by dividing the total sample of interviews allocated to the LGA by the required number of interviews.
- The minimum number of interviews per sampled wards will be 10.

| **Location** | **Interviews** |
| --- | --- |
| Nasarawa | 166 |
| Kano | 166 |
| Sokoto | 167 |
| Enugu | 167 |
| Rivers | 167 |
| Lagos | 167 |
| Total | 1000 |

## Sampling Protocol

### Stage 1

We will randomly select LGAs from the list of the available LGAs within the state based on urban/rural split of the state, using a list from the National Bureau of Statistics. This first stage involves breaking down the target population by urban/rural split of the state to get the first-order sample distribution.

Below is the urbanization split by state and the resulting sampling allocations.

| **State** | **Urban%** | **Rural%** |
| --- | --- | --- |
| Lagos | 95% | 5% |
| Kano | 29% | 71% |
| Enugu | 19% | 81% |
| Nasarawa | 24% | 76% |
| Sokoto | 22% | 78% |
| Rivers | 48% | 52% |

| **Location** | **Caregivers Total** | **Urban** | **Rural** |
| --- | --- | --- | --- |
| Nasarawa | 166 | 40 | 126 |
| Kano | 166 | 50 | 116 |
| Sokoto | 167 | 37 | 130 |
| Enugu | 167 | 31 | 136 |
| Rivers | 167 | 80 | 87 |
| Lagos | 167 | 157 | 10 |
| Total | 1000 | 500 | 500 |

### Stage 2

This stage involves a random selection of the sectors/wards within the community based on the number of interviews to be conducted in that locality. The ward will form the Primary Sampling Units (PSUs). To determine the number of PSUs to be selected in each LGA, a ‘sample take’ value will be considered. The ‘sample take’ is the number of interviews to be done in each PSU – that is 10 interviews per ward.

By dividing the ‘sample take’ with the sample allocation at the LGA level, the number of required PSUs in the LGA is determined. From a list of all PSUs in each LGA, the PSUs will be selected with equal probability.

In the case of large ward they will be further segmented (and a random segment selected for sampling) to ensure that it is possible to cover the area with a random walk technique. If the number of the streets within the ward is more than 20, the wards is considered large. The streets within the Ward will be listed and 15 will be randomly selected to sample from

### Stage 3

This involves a random selection of starting point. We will list the available streets within the ward and will randomly select one street where the day’s work will start.

### Stage 4

This involves a random selection of dwelling structures based on the day’s code. Following the left-hand rule, the interviewer will proceed to the starting dwelling structure which is determined by the date (i.e. on 28th of the month the starting dwelling structure will be house number 10 [2+8] from the fixed starting point). After a successful interview in a dwelling structure, a sampling gap of 1-in-3 and 1-in-5 is observed within rural and urban sectors respectively.

### Stage 5

This involves a random selection of a household based on day’s code (if there is more than one household within a dwelling structure). Following the left-hand rule, the interviewer will proceed to the starting household which is determined by the date (i.e. on 28th of the month the starting household will be household number 10 [2+8]. The household is screened according to the screening criteria and appropriate quotas.

The interviewers at all times must keep a contact sheet where they record each and every household they call on and whether the interview was successful or not and reasons for non-successful interviews.

Definition of a household: A household is defined by all those who satisfy at least two of the following three conditions: (1) share the same food pot, (2) share the same roof or (3) have a common decision maker. Home-helps and other live-in employees of the household should be excluded from the Kish grid.

### Stage 6

This involves selection of an eligible respondent based on recruitment criteria. The individual respondent will form the Ultimate Sampling Units (USUs).

Only one interview will be conducted in the selected household.

A kish grid will be applied to randomly select the respondent to be interviewed in the selected household.

### Substitution Process

If no one is available at the household, the fieldwork team will continue to implement the selection procedure until the area’s quota is filled. Therefore, households were skipped if there was no answer.

### Modifications during fieldwork

No modifications were made during fieldwork. A sample of 1264 was achieved. This oversampling was due to simultaneous fieldwork in several regions, meaning totals were only produced at the end of each day. This results in small differences between the intended samplings for each group compared to what was achieved.

# Uganda Sampling Protocol

## Sample Size

1,000 interviews with caregivers

## Sample Definition

- Must have at least one child
- Must reside in one of the study sites
- No restrictions in terms of age, income, level of education or ethnic background.
- Must have primary caring responsibilities for a child between the age of 1 and 3 years
  - This is to ensure that we only interview caregivers of children who have had the opportunity to have all recommended vaccines in the 0-12 months age range. We have restricted the age to maximum 3 years so that we do not rely on recall that is too distant.
  - If participants have multiple eligible children, the interview will focus on the youngest eligible child.
  - If the youngest are twins/ triplets etc., a child will be randomly selected using a Kish grid

## Sample allocation requirements

- The sample will be equally distributed across the five sub-regions. The 2020 Housing Census Population Projection was used for regional population sizes.
- Rural/urban interviews will be allocated 78% and 22% of the sample respectively, as per national statistics.
- The number of enumeration areas (EAs) per sampled district will be determined by the number of interviews allocated to the district. This will be done by dividing the total sample of interviews allocated to the district by the required number of interviews.
- The minimum number of interviews per sampled EA is 10.

## Sampling Protocol

### Stage 1

The target population is broken down by the first order geographical boundaries (region) by setting (urban/rural) to get the first-order sample distribution. The sample will be equally allocated in the regions and according to population statistics within each setting as shown in the table below.

| **Sub-Region** | **Rural (0.78)** | **Urban (0.22)** |
| --- | --- | --- |
| Kampala | 0 | 200 |
| North Central | 160 | 40 |
| Bukedi | 160 | 40 |
| Acholi | 160 | 40 |
| Ankole | 160 | 40 |
| Total | 640 | 360 |

#### Stage 2

In order to achieve a representative distribution of districts, the districts will be ordered in a geographical serpentine order. In each of the regions, the selected districts will also be stratified by urban and rural setting and the sample will be allocated by population size drawn from 2020 Population Projections and 2014 Census Sampling frame. The sample of each district will be calculated based on its respective population.

Kampala being a capital city is predetermined to represent the Central I region because of its unique socio-economic and demographic profile. All interviews in Kampala will be considered urban.

Districts with a sample allocation of less than 10 interviews per EA will be dropped from the final selected list of districts and their sample re-allocated to the nearby districts within the sub-region.

The final sample allocation per selected district will be rounded off to the nearest 10 to allow for uniformity with 10 interviews per EA.

#### Stage 3

The ward/ enumeration area will form the Primary Sampling Units (PSUs). To determine the number of PSUs to be selected in each district, a ‘sample take’ value will be considered. The ‘sample take’ is the number of interviews to be done in each PSU – that is 10 interviews per enumeration area.

By dividing the ‘sample take’ with the sample allocation at the district level, the number of required PSUs in the districts is determined. From a list of all PSUs in each district, the PSUs are selected with equal probability, taking into consideration the urban and rural stratification within the district.

In the case of large wards/ enumeration areas they will be further segmented (and a random segment selected for sampling) to ensure that it is possible to cover the area with a random walk technique.

#### Stage 4

Households will form the Secondary Sampling Units (SSUs). Instead of starting from a local landmark (a technique which can be open to bias), pre-selected random coordinates in each of the wards/ EAs will be selected.

Following the left-hand rule, the interviewer will proceed to the starting household which is determined by the date (i.e. on 28th of the month the starting household will be house number 10 [2+8] from the fixed starting point). The household is screened according to the screening criteria and appropriate quotas.

If the household screens into the study, this household forms the first household at which an interview can be conducted. After completing an interview (using the left-hand rule) 4 households are skipped thereby calling on the 5th household. The interviewers at all times must keep a call sheet where they record each and every household they call on and whether the interview was successful or not and reasons for non-successful interviews.

Definition of a household: A household is defined by all those who satisfy at least two of the following three conditions: (1) share the same food pot, (2) share the same roof or (3) have a common decision maker. Home-helps and other live-in employees of the household should be excluded from the Kish grid.

#### Stage 5

The individual respondent will form the Ultimate Sampling Units (USUs).

Only one interview will be conducted in the selected household.

A kish grid will be applied to randomly select the respondent to be interviewed in the selected household.

### Substitution Process

Households will only be substituted after three unsuccessful call backs at the household. The supervisor must ascertain and log that all the call-backs were unsuccessful. The supervisor will ascertain the reasons why the interviewer was not able to interview the selected participant. If the participant is ill or incapacitated the interviewer will use the Kish grid again to select another participant. Substitution will also be applied when respondents explicitly refuse to be interviewed.

### Modifications during fieldwork

A sample of 1054 caregivers was achieved. This oversampling was due to simultaneous fieldwork in several regions, meaning totals were only produced at the end of each day. This results in small differences between the intended samplings for each group compared to what was achieved.

In addition, purposive sampling was used towards the end of the fieldwork to ensure that we achieved a high enough sample of unvaccinated children for analysis.

# Guinea Sampling Protocol

## Sample Size

1,000 interviews with caregivers

## Sample Definition

- Must have at least one child
- Must reside in one of the study sites
- No restrictions in terms of age, income, level of education or ethnic background.
- Must have primary caring responsibilities for a child between the age of 1 and 3 years
  - This is to ensure that we only interview caregivers of children who have had the opportunity to have all recommended vaccines in the 0-12 months age range. We have restricted the age to maximum 3 years so that we do not rely on recall that is too distant.
  - If participants have multiple eligible children, the interview will focus on the youngest eligible child.
  - If the youngest are twins/ triplets etc., a child will be randomly selected using a Kish grid

## Sample allocation requirements

- The sample for all respondent categories will be equally distributed across the five sub-regions
- The rural/urban split will be approximately 80%/20%, as per national statistics. Population figures were drawn from 2014 population census data.
- The list of localities and districts in the target regions was taken from the national census data portal and segmented by urban and rural locations

## Sampling Protocol

### Stage1

This first stage involves breaking down the target population by the first order geographical boundaries (sub-regions) by setting (urban/rural) to get the first-order sample distribution. The sample will be equally allocated across the 5 sub-regions and within each setting as shown in the table below.

|  | **Sample Distribution** | |
| --- | --- | --- |
| **Sub-Region** | **Rural (0.80)** | **Urban (0.20)** |
| Conakry | 0 | 200 |
| Boke | 160 | 40 |
| Mamou | 160 | 40 |
| Kankan | 160 | 40 |
| N’zérékoré | 160 | 40 |
| Total | 640 | 360 |

### Stage 2

In each sub-region, 10 study sites (at locality level in rural areas and at district level in urban areas) will be selected. 20 interviews will be allocated to each locality or district.

### Stage 3

The selection of Primary Sampling Units (PSUs , also known as enumeration areas or EAs) within the selected districts/localities will be selected.

Urban:

- Sub-regions, localities and districts will be pre-selected before fieldwork has begun
- In each district, 2 EAs should be identified during fieldwork. Ideally all areas will be listed and then 2 chosen randomly; otherwise as many as possible are listed and then chosen randomly
- A starting point is identified per each EA (e.g. a landmark or meeting place) and interviewers go in opposite directions

Rural:

- Only sub-region and locality are pre-determined in advance, due to lack of data availability at lower levels
- In each locality, 2 districts will be selected as randomly as possible. This is done by the fieldwork supervisor who will list all districts in the selected area and make a random selection
- In each district, one enumeration area will be selected by the supervisor (following the procedure for urban areas)
- In the enumeration area, a starting point will be selected, as in urban areas

### Stage 4

Households will form the Secondary Sampling Units (SSUs)

Following the left-hand rule, the interviewer will proceed to the starting household which is determined by the date (i.e. on 28th of the month the starting household will be house number 10 [2+8] from the fixed starting point). The household is screened according to the screening criteria and appropriate quotas.

If the household screens into the study, this household forms the first household at which an interview can be conducted. After completing an interview (using the left-hand rule) a number of households are skipped (one in every 3 households in rural areas and one in every 5 in urban areas). The interviewers at all times must keep a call sheet where they record each and every household they call on and whether the interview was successful or not and reasons for non-successful interviews.

Definition of a household: A household is defined by all those who satisfy at least two of the following three conditions: (1) share the same food pot, (2) share the same roof or (3) have a common decision maker. Home-helps and other live-in employees of the household should be excluded from the Kish grid.

### Stage 5

This involves selection of an eligible respondent based on recruitment criteria. The individual respondent will form the Ultimate Sampling Units (USUs).

Only one interview will be conducted in the selected household.

A kish grid will be applied to randomly select the respondent to be interviewed in the selected household.

### Substitution Process

Households at which no-one is present will be revisited once before a new selection is made according to the above protocol. In some instances, regional quotas were filled before a return visit could be made, and so the original household was not revisited.

### Modifications during fieldwork

Due to flooding in Mamou and Boké during the fieldwork period, two districts had to be replaced during fieldwork. This was done non-randomly to approximate the profile of the original selection as closely as possible, and to ensure safe access for the enumeration teams.

Due to simultaneous fieldwork in several regions, totals were only produced at the end of each day. This results in small differences between the intended samplings for each group compared to what was achieved.
