# Supplementary material for: A structural equation modelling approach to understanding the determinants of childhood vaccination in Nigeria, Uganda and Guinea
Source: PLOS Glob Public Health. 2023 Mar 29;3(3):e0001289. doi: 10.1371/journal.pgph.0001289 (PMC10058155; doi:10.1371/journal.pgph.0001289)
Supplement: S2 Text — (DOCX) [file pgph.0001289.s003.docx]

**A Structural Equation Modelling Approach to Understanding the Determinants of Childhood Vaccination in Nigeria, Uganda and Guinea**

**Supplementary Material 2**

**Questionnaires**

Nigeria and Uganda Page 2

Guinea Page 39Nigeria and Uganda

Ipsos Healthcare

3 Thomas More Square, London, E1W 1YW, UK

Tel: + 44 20 3059 5000 Fax: +44 20 3059 4998

Internal client use only

**Vaccines Demand
19-024660-01
Screener and Questionnaire**

**Sample quotas:**

|  | Nigeria | Uganda |
| --- | --- | --- |
| **Caregivers** | 1000 | 1000 |

**Methodology: Face to face questionnaire**

**Interview length: 40 minutes**

INTRODUCTION

I now need to read you a statement to let you know about your rights and how you will be protected during and after this discussion. It may be a little long but is important so please be patient with me

NIGERIA

I am an interviewer working for a research company called Ipsos doing research for a health organisation.

This research study aims to understand peoples’ experiences and thoughts on their community and health so that health services can be made better.

If you agree to take part, I, as a trained interviewer, will spend around 40 minutes with you and ask you questions about your opinions.

This research is completely voluntary and you are free to withdraw from the study at any time, including during the research or after it has taken place. If you choose to withdraw, nothing bad will happen.

To thank you for your time, participation and any inconvenience you will receive a small token of appreciation.

In addition to asking about what you think and do, we will also collect your name, telephone number and address so that we can contact you if needed. You may be recontacted up to 4 weeks after this interview.

All information about who you are and where you live will be kept on a secure computer which will not be available to others and will be kept confidential to the extent possible by law. The only people that will see your responses will be those doing the research or those checking that the research was done properly, and they are required to keep all details confidential.

Your answers will be stored securely on a computer and used for research purposes in ways that will not reveal who you are. All future use of the stored data will come after a Research Ethics Committee (a group who oversee if research is done properly) has approved its use.

The most significant risk would be that someone would find out that we spoke to you but we have put things in place to ensure that this does not happen, such as storing your details on a password protected computer and destroying them soon after this study.

We can do this interview wherever you want and you can refuse to answer any of the questions or stop the interview at any stage.

this study may be extremely helpful in that it will provide information to improve health services in the country.

If you would like to receive feedback on our study, we will record your phone number on a separate sheet of paper and can send you the results of the study when it is completed. This information will also be stored securely and only viewed by those who are required to see it.

This research has been approved by the National Health Research Ethics Committee. If you have any complaints about ethical aspects of the research or feel that you have been harmed in any way by participating in this study, please contact 234-9-523-8367

If you have any questions about the research, you may call the project leader Taiwo Sanni ((+234) 8 066 299 805)

| The research has been explained to me and I have been given a full explanation about the possible uses of any personally identifying information, either by reading this form or having it read to me | | YES □  NO □ **TERMINATE** |
| --- | --- | --- |
| I consent to participating in this research | | YES □  NO □ **TERMINATE** |
| Are you happy for Ipsos to re-contact you regarding this research? | | YES □ NO □ |
| Print full name |  | |
| Signature |  | |
| Date |  | |
| Telephone number |  | |
| Email address | **OPTIONAL** | |

**UGANDA**

I am an interviewer working for a research company called Ipsos doing research for a health organisation.

This research study aims to understand peoples’ experiences and thoughts on their community and health so that health services can be made better.

If you agree to take part, I, as a trained interviewer, will spend around 40 minutes with you and ask you questions about your opinions.

This research is completely voluntary and you are free to withdraw from the study at any time, including during the research or after it has taken place. If you choose to withdraw, nothing bad will happen.

To thank you for your time, participation and any inconvenience you will receive a small token of appreciation.

In addition to asking about what you think and do, we will also collect your name, telephone number and address so that we can contact you if needed. You may be recontacted up to 4 weeks after this interview.

All identifying information will be kept on a secure computer which will not be available to others and will be kept confidential to the extent possible by law. The only people that will see your responses will be those conducting the research or those checking that the research was done properly, and they are required to keep all details confidential.

Your answers will be stored securely on a computer and used for research purposes in ways that will not reveal who you are. All future use of the stored data will come after a Research Ethics Committee (a group who oversee if research is done properly) has approved its use.

The most significant risk would be that someone would find out that we spoke to you but we have put things in place to ensure that this does not happen such as storing your details on a password protected computer and destroying them soon after this study.

We can do this interview wherever you want and you can refuse to answer any of the questions or stop the interview at any stage.

this study may be extremely helpful in that it will provide information to improve health services in the country.

If you would like to receive feedback on our study, we will record your phone number on a separate sheet of paper and can send you the results of the study when it is completed. This information will also be stored securely and only viewed by those who are required to see it.

This research has been approved by Makerere School of Public Health. If you have any complaints about ethical aspects of the research or feel that you have been harmed in any way by participating in this study, please contact

The Chair

Makerere School of Public Health

Research Ethics Committee

Telephone: 0772 96 30 74

E-mail: [jssemps@musph.ac.ug](mailto:jssemps@musph.ac.ug)

And,

The Executive Secretary

The Uganda National Council of Science and Technology,

Kimera Road. Ntinda P. O. Box 6884 Kampala, Uganda

Telephone: (256) 414 705500

Fax: +256-414-234579

Email: [info@uncst.go.ug](mailto:info@uncst.go.ug)

If you have any questions about the research, you may call the project leader Emmanuel Odongo + 256 773 132 981 or James Ssenkungu +256 772 233272

Consent statement/question

| The research has been explained to me and I have been given a full explanation about the possible uses of any personally identifying information, either by reading this form or having it read to me | | YES □  NO □ **TERMINATE** |
| --- | --- | --- |
| I consent to participating in this research | | YES □  NO □ **TERMINATE** |
| I consent to the use of my audio recording as described above | | YES □  NO □ |
| Are you happy for Ipsos to re-contact you regarding this research? | | YES □ NO □ |
| Print full name |  | |
| Signature/ thumb print |  | |
| Name and witness signature (if thumb print used) |  | |
| Date |  | |
| Telephone number |  | |
| Email address | **OPTIONAL** | |

**SCREENER**

I will start with a short set of questions to determine if you meet the inclusion criteria for the survey.

ASK ALL

S1. INTERVIEWER RECORD: COUNTRY

SINGLE ANSWER

| **Code Number** | **Code Label** | **Scripting / Routing Instruction** |
| --- | --- | --- |
|  | Nigeria |  |
|  | Uganda |  |

ASK ALL

S2. Which language would you prefer for the interview?

SINGLE ANSWER

NIGERIA

| **Code Number** | **Code Label** | **Scripting / Routing Instruction** |
| --- | --- | --- |
|  | English | SHOW IF S1=1 |
|  | Hausa | SHOW IF S1=1 |
|  | Yoruba | SHOW IF S1=1 |
|  | Igbo | SHOW IF S1=1 |

UGANDA

| **Code Number** | **Code Label** | **Scripting / Routing Instruction** |
| --- | --- | --- |
|  | English | SHOW IF S1=2 |
|  | Luganda | SHOW IF S1=2 |
|  | Samia | SHOW IF S1=2 |
|  | Japadhola | SHOW IF S1=2 |
|  | Runyankole | SHOW IF S1=2 |
|  | Acholi | SHOW IF S1=2 |

ASK ALL

S3. [NIGERIA] INTERVIEWER RECORD STATE [UGANDA] INTERVIEWER RECORD DISTRICT

SINGLE ANSWER

NIGERIA

| **Code Number** | **Code Label** | **Scripting / Routing Instruction** |
| --- | --- | --- |
|  | Nasarawa | SHOW IF S1=1 |
|  | Kano | SHOW IF S1=1 |
|  | Sokoto | SHOW IF S1=1 |
|  | Enugu | SHOW IF S1=1 |
|  | Rivers | SHOW IF S1=1 |
|  | Lagos | SHOW IF S1=1 |
|  | Other | SHOW IF S1=1  TERMINATE |

UGANDA

| **Code Number** | **Code Label** | **Scripting / Routing Instruction** |
| --- | --- | --- |
|  | Kampala | SHOW IF S1=2 |
|  | North Central/ Central 1 | SHOW IF S1=2 |
|  | Bukedi (East) | SHOW IF S1=2 |
|  | Acholi (North) | SHOW IF S1=2 |
|  | South-west | SHOW IF S1=2 |
|  | Other | SHOW IF S1=2  TERMINATE |

ASK ALL

S4. INTERVIEWR: RECORD LOCATION

SINGLE ANSWER

| **Code Number** | **Code Label** | **Scripting / Routing Instruction**  **QUOTAS** |
| --- | --- | --- |
|  | Urban | ADD UP CODES FOR THE QUOTAS |
|  | Peri-urban |  |
|  | Rural |  |

ASK ALL

S5. How old were you at your last birthday?

SINGLE ANSWER ONLY

|  |  |
| --- | --- |

| **Code Number** | **Code Label** | **Scripting / Routing Instruction** |
| --- | --- | --- |
|  | Less than 18 | TERMINATE |
|  | 18-24 |  |
|  | 25-29 |  |
|  | 30-34 |  |
|  | 35-39 |  |
|  | 40-44 |  |
|  | 45-49 |  |
|  | 50-65 |  |
|  | 65+ |  |

IF PARTICIPANT DOESN’T WANT TO SHARE THIS INFORMATION, PLEASE TERMINATE. WE CANNOT INTERVIEW PEOPLE UNDER 18

ASK ALL

S6. How many children, of any age, do you have? This could be biological children, or children you have adopted/ look after permanently.

NUMERIC ENTRY

|  | *Child/ children* |
| --- | --- |

MIN – 0; MAX – 30

TO TERMINATE IF 0

ASK ALL

S6a. How many children do you have between ages 12 – 36months (1-3 years)?

NUMERIC ENTRY

|  | *Child/ children* |
| --- | --- |

MIN – 0; MAX – 30. SHOULD NOT BE GREATER THAN NUMBER AT S6

TO TERMINATE IF 0

ASK IF S6A >1

S6b. Of your children between 12-36 months (1-3 years), what are their names and ages?

| **Code Number** | **Code Label A)** | **B)** | **C) Name** | **Scripting / Routing Instruction** |
| --- | --- | --- | --- | --- |
|  | ____xx__years | OR __ xx ____months |  |  |

INSERT NUMBER OF ROWS = NUMBER AT S6a

SHOW IF S6b ASKED:

Today we will be talking about [INSERT NAME OF YOUNGEST CHILD AT S6B]. So please think about this child for all future question

PN: IF MORE THAN ONE CHILD ARE THE SAME AGE AND THE YOUNGEST BETWEEN 12 AND 36 MONTHS, PROGRAMME SHOULD RANDOMLY SELECT ONE

ASK ALL

S7. Are you the primary guardian or person responsible for providing day to day care to [INSERT NAME OF YOUNGEST CHILD AT S6B ] ?

SINGLE ANSWER

| **Code Number** | **Code Label** | **Scripting / Routing Instruction** |
| --- | --- | --- |
|  | Yes | TO CONTINUE |
|  | No | TO TERMINATE |

TO TERMINATE IF CODE 2 SELECTED

ASK ALL

S7b. What is your relation with [INSERT NAME OF YOUNGEST CHILD AT S6B]?

SINGLE ANSWER, DON’T READ OUT LIST

| **Code Number** | **Code Label** | **Scripting / Routing Instruction** |
| --- | --- | --- |
|  | Biological mother |  |
|  | Stepmother |  |
|  | Aunt |  |
|  | Grandmother |  |
|  | Biological father |  |
|  | Stepfather |  |
|  | Other | CLOSE |

S7c. What gender is [INSERT NAME OF YOUNGEST CHILD AT S6B]?

SINGLE ANSWER

| **Code Number** | **Code Label** | **Scripting / Routing Instruction**  **QUOTAS** |
| --- | --- | --- |
|  | Male | CONTINUE |
|  | Female | CONTINUE |
|  | Prefer not to say | CONTINUE |

ASK ALL

S8. Thinking about [INSERT NAME OF YOUNGEST CHILD AT S6B ], did he/she receive any vaccines before the age of 1? By vaccine I mean something that is given to people, when they are not ill, to try to stop them from getting certain diseases. They are usually given as an injection, but sometimes given by mouth

SINGLE ANSWER

| **Code Number** | **Code Label** | **Scripting / Routing Instruction** |
| --- | --- | --- |
|  | Yes | TO CONTINUE |
|  | No | TO CONTINUE |
|  | Don’t know/ Don’t remember | TO CONTINUE |

ASK IF S8= 1 0R 3

S9. Do you have a card/book for [INSERT NAME OF YOUNGEST CHILD AT S6B ] that records which immunisations he/she has had?

SINGLE ANSWER

| **Code Number** | **Code Label** | **Scripting / Routing Instruction** |
| --- | --- | --- |
|  | Yes | TO CONTINUE |
|  | No | TO TERMINATE IF ANSWERED DON’T KNOW AT QS8 |

ASK IF [S8=1] OR [S8=3 AND S9=1] (VACCINATED)

S10. Please think about the times when [INSERT NAME OF YOUNGEST CHILD AT S6B ] was given vaccines. At any of these times…

SINGLE ANSWER PER QUESTION

PLEASE ADD A YES/NO OPTION FOR EACH ROW

| **Code Number** | **Code Label** | **Scripting / Routing Instruction/**  **Quota** |
| --- | --- | --- |
|  | …Was there a reminder from the health facility that this was the time to get that vaccination? | YES: Routine |
|  | …Did someone come to your area specifically to do the vaccinations? | YES: Outreach/ Campaign |
|  | Did the vaccination take place in a clinic/ hospital/ outreach post? | YES Routine/ campaign |
|  | Did the vaccination take place in a public place (in a tent/ public hall?) | YES Outreach/ campaign |

ASK IF [S8=1] OR [S8=3 AND S9=1] (VACCINATED)

S11. IF VACCINATION CARD AVALABLE, REFER TO VACCINATION CARD AND RECORD WHICH VACCINATIONS HAVE BEEN ADMINISTERED- ONLY IN THE FIRST 12 MONTHS (I.E. DO NOT INCLUDE VACCINES WHICH WERE GIVEN AFTER 1 YEAR OLD). IF CARD NOT AVAILABLE, READ OUT THE LIST

MULTICODE

Which vaccinations has your child had?

|  | **At birth** | |
| --- | --- | --- |
| **1** | **BCG** (birth) (usually given in the left upper arm, or shoulder, which usually causes a scar) |  |
| **2** | **Hepatitis B** paediatric dose (birth )  (injection in their thigh at birth or soon after birth) | DO NOT SHOW IN UGANDA |
| **3** | **Oral polio**  (birth) (drops in the mouth) |  |
|  | **At 6 weeks:** | |
| **4** | **Oral polio**  (drops in the mouth) |  |
| **5** | **Pentavalent** (diphtheria, tetanus, pertussis, Haemophilus influenzae B and Hepatitis B (6 weeks) (injection in their left upper thigh) |  |
| **6** | **Pneumococcal** conjugate vaccine (injection on the right upper thigh) |  |
| **7** | **Rotavirus**  (drops in mouth) | SHOW IN UGANDA IF CHILD IS 1 YEAR OLD . DO NOT SHOW IN NIGERIA |
|  | **At 10 weeks:** | |
| **8** | **Oral polio**  (drops in the mouth) |  |
| **9** | **Pentavalent** (diphtheria, tetanus, pertussis, Haemophilus influenzae B and Hepatitis B (6 weeks) (injection in their left upper thigh) |  |
| **10** | **Pneumococcal** conjugate vaccine (injection on the right upper thigh) |  |
| **11** | **Rotavirus**  (drops in mouth) | SHOW IN UGANDA IF CHILD IS 1 YEAR OLD. DO NOT SHOW IN NIGERIA |
|  | **At 14 weeks:** | |
| **12** | **Oral polio**  (drops in the mouth) |  |
| **13** | **Pentavalent** (diphtheria, tetanus, pertussis, Haemophilus influenzae B and Hepatitis B (6 weeks) (injection in their left upper thigh) |  |
| **14** | **Pneumococcal** conjugate vaccine (injection on the right upper thigh) |  |
| 15 | IPV (**inactivated polio** vaccine) (14 weeks)  (injection usually given on right thigh) |  |
|  | **At 9 months:** | |
| **16** | **Measles** (9 months) (injection in left upper arm) |  |

VACCINATION STATUS VARIABLE// QUOTA

NIGERIA

| NOT VACCINATED | *S11 –ALL CODES ARE 0*  OR  S8=2 |
| --- | --- |
| PARTIALLY VACCINATED | S11 – NUMBER OF CODES SELECTED RANGE FROM 1-13 (NIGERIA) |
| FULLY VACCINATED | S11– ALL CODES SELECTED 14 (NIGERIA) |

UGANDA

| NOT VACCINATED | *S11 –ALL CODES ARE 0*  OR  S8=2 |
| --- | --- |
| PARTIALLY VACCINATED | S11 – NUMBER OF CODES SELECTED RANGE FROM 1-14 (UGANDA) IF CHILD 1 YEAR  S11 – NUMBER OF CODES SELECTED RANGE FROM 1-12 (UGANDA) IF CHILD OLDER THAN 1 YEAR |
| FULLY VACCINATED | S11– ALL CODES SELECTED 14 (UGANDA) IF CHILD 1 YEAR  S11– ALL CODES SELECTED 12 (UGANDA) IF CHILD OLDER THAN 1 YEAR |

ASK IF [S8=1] OR [S8=3 AND S9=1] (VACCINATED)

S12. Did your child go to private health facilities, public facilities, or a mixture of both to get vaccinated? SINGLE ANSWER

| **Code Number** | **Code Label** | **Scripting / Routing Instruction** |
| --- | --- | --- |
|  | All vaccines in private clinic/hospital | TO TERMINATE |
|  | Some in private clinic and some in public clinic/hospital/health centre | TO CONTINUE |
|  | All vaccines in public clinic/hospital/health centre or outreach session | TO CONTINUE |
|  | Don’t know/ prefer not to answer | CLOSE |

ASK ALL

S13. What is the highest level of formal education you have completed?

SINGLE ANSWER

| **Code Number** | **Code Label** | **Scripting / Routing Instruction** |
| --- | --- | --- |
|  | No formal education |  |
|  | Some primary (not finished) |  |
|  | Primary completed |  |
|  | Some secondary (not finished) |  |
|  | Secondary completed |  |
|  | Technical College completed |  |
|  | University (Bachelors) degree completed |  |
|  | Master’s degree completed |  |
|  | PhD |  |
|  | Prefer not to answer |  |

ASK ALL

S14. What is the typical monthly income for your household?

INTERVIEWER: WE ASSUME HERE GROSS HH INCOME IN CASE YOU NEED TO SPECIFY IT TO THE PARTICIPANT

NIGERIA

SINGLE ANSWER

| **Code Number** | **Code Label** | **Scripting / Routing Instruction** |
| --- | --- | --- |
|  | 9,999 naira or below | **LOW- CHECK QUOTAS** |
|  | 10,000 to 50,000 naira | **LOW- CHECK QUOTAS** |
|  | 50,001 to 200,000 naira | **MIDDLE- CHECK QUOTAS** |
|  | 200,001 to 500,000 naira | **MIDDLE- CHECK QUOTAS** |
|  | 500,001 to 800,000 naira | **MIDDLE- CHECK QUOTAS** |
|  | 800,001 to 900,000 naira | **HIGH- CHECK QUOTAS** |
|  | 900,001 to 1 million naira | **HIGH- CHECK QUOTAS** |
|  | More than 1 million naira | **HIGH- CHECK QUOTAS** |
|  | Prefer not to answer |  |

UGANDA

SINGLE ANSWER

| **Code Number** | **Code Label** | **Scripting / Routing Instruction**  **QUOTAS** |
| --- | --- | --- |
|  | Below 2,000,000 ugx | **LOW- CHECK QUOTAS** |
|  | 2,000,000 - 3,000,000 ugx | **MIDDLE- CHECK QUOTAS** |
|  | 3,000,001 - 4,000,000 ugx | **MIDDLE- CHECK QUOTAS** |
|  | 4,000,001 - 5,000,000 ugx | **MIDDLE- CHECK QUOTAS** |
|  | 5,000,001 - 5,500,000 ugx | **HIGH- CHECK QUOTAS** |
|  | 5,500,001 - 6,000,000 ugx | **HIGH- CHECK QUOTAS** |
|  | Over 6,000,000 ugx | **HIGH- CHECK QUOTAS** |
|  | Prefer not to answer |  |

**SECTION A: DEMOGRAPHICS**


Thank you for the information you have provided to us first of all, your answers show that you are able to take part in the research. To start our interview, I’m going to ask you about a few questions about yourself, your family and your household

ASK ALL

A1. What is your relationship status?

SINGLE ANSWER

CODES 1, 3 AND 4 ARE UNIQUES. ONLY CODES 2 ,5 AND 6 COULD BE SELECTED SIMULTANEOUSLY

| **Code Number** | **Code Label** | **Scripting / Routing Instruction** |
| --- | --- | --- |
|  | Single | SINGLE CODE |
|  | In a relationship (not married) | MULTICODE |
|  | Married, I’m the only wife | SINGLE CODE |
|  | Married, I’m NOT the only wife | SINGLE CODE |
|  | Separated/ Divorced | MULTICODE |
|  | Widowed | MULTICODE |

ASK IF A1= 3,4,5 OR 6

A2. At what age did you get married?

NUMERIC ENTRY

|  | *Years old* |
| --- | --- |

MIN – 0; MAX – 50

ASK ALL

A3. How many people live in your household, including yourself, all children, all family members and house help?

NUMERIC ENTRY

|  | *people* |
| --- | --- |

MIN – 2; MAX – 50

ASK IF A3=2 OR MORE

A4. Who do you live with in your household?

MULTIPLE ANSWER. DO NOT READ OUT LIST

| **Code Number** | **Code Label** | **Scripting / Routing Instruction** |
| --- | --- | --- |
|  | My spouse/ partner |  |
|  | My mother |  |
|  | My father |  |
|  | My mother in law |  |
|  | My father in law |  |
|  | My husband’s other wives | ONLY SHOW IF A1=4 |
|  | My brothers in law |  |
|  | My sisters in law |  |
|  | My brothers |  |
|  | My sisters |  |
|  | My grandparents in law |  |
|  | My grandmother |  |
|  | My grandfather |  |
|  | Cousins |  |
|  | Uncles |  |
|  | Aunties |  |
|  | Family friends |  |
|  | House help |  |
|  | Someone else’s children |  |
|  | My child(ren) |  |
|  | Others (specify) |  |

**ASK ALL**

A5. Which of the following best describes your employment status?

MULTICODE ANSWER

| **Code Number** | **Code Label** | **Scripting / Routing Instruction** |
| --- | --- | --- |
|  | Employed by someone else / a company (part-time or full time) |  |
|  | Self-employed (part-time or full time) |  |
|  | Unemployed |  |
|  | Student |  |
| 98 | Other (specify) | Anchor at bottom |

ASK IF A5=1 OR 2

A6. What is your occupation?

MULTI ANSWER, DO NOT READ OUT LIST

| **Code Number** | **Code Label** | **Scripting / Routing Instruction** |
| --- | --- | --- |
|  | Farmer (including crops and animals, hunting and fishing) |  |
|  | Mining and quarrying |  |
|  | Work in a factory |  |
|  | Construction (e.g. builder, engineer) |  |
|  | Trade (e.g. shopkeeper, works on market stall) |  |
|  | Accommodation and food services (e.g. works in hotel, restaurant, café) |  |
|  | Transport (e.g. driver) |  |
|  | Information and communication (e.g. works in television or film industry, computer programmer) |  |
|  | Finance and insurance (e.g. bank worker) |  |
|  | A profession (e.g. lawyer, accountant, architect, scientific researcher, advertising and marketing) |  |
|  | Administrator (e.g. office administrator) |  |
|  | Government employee (e.g. civil servant) |  |
|  | Education (e.g. teacher) |  |
|  | Healthcare worker (e.g. doctor, nurse, social worker) |  |
| 98 | Other (specify) | Anchor at bottom |

ASK IF A5= 1 0R 2

A7. How many hours of paid work did you do last week?

|  | *Hours* |
| --- | --- |

NUMERICAL INPUT

RANGE (0-120)

ASK ALL

A8. Which mode of transport would you most likely take to each of these places?

SINGLE ANSWER PER COLUMN

| **The nearest public clinic / health centre/ health post** | **The private clinic / health centre/ health post** | **The nearest hospital** | **The nearest pharmacy** |
| --- | --- | --- | --- |
| 1 | 2 | 3 | 4 |

DOWN SIDE OF GRID

| **Code Number** | **Code Label** | **Scripting / Routing Instruction** |
| --- | --- | --- |
|  | Walking |  |
|  | Bus |  |
|  | Boda boda/ motorcycle taxi |  |
|  | Taxi (Car) |  |
|  | Car (mine/family/friend) |  |
|  | Keke napep (yellow scooter vehicles) | [SHOW IN NIGERIA ONLY] |
|  | Bicycle |  |
|  | Other (specify) |  |

ASK ALL

A8a. And how long would it take to reach that place by that mode of transport?

SINGLE ANSWER PER COLUMN

| **The nearest public clinic / health centre/ health post** | **The nearest private clinic / health centre/ health post** | **The nearest hospital** | **The nearest pharmacy** |
| --- | --- | --- | --- |
| 1 | 2 | 3 | 4 |

DOWN SIDE OF GRID

| **Code Number** | **Code Label** | **Scripting / Routing Instruction** |
| --- | --- | --- |
|  | 0-15 minutes |  |
|  | 16-30 minutes |  |
|  | 31-60 minutes |  |
|  | 61-120 minutes |  |
|  | More than 120 minutes |  |
|  | Don’t know |  |

ASK ALL

A9. Since the COVID-19 pandemic started at the beginning of 2020, how, if at all, has your family’s life changed?

MULTI ANSWER, DO NOT READ OUT LIST

INTERVIEWER: PROBE IF THEY SAY, ‘NO CHANGES’ BEFORE SELECTING OPTION 18.

ASK THEM WHETHER, THEIR INCOME HAS STAYED THE SAME, THEIR LIVING ARRANGEMENT IS EXACTLY THE SAME, THEIR CHILDREN HAVE BEEN GOING TO SCHOOL/NURSERY AS EXAMPLES

| **Code Number** | **Code Label** | **Scripting / Routing Instruction** |
| --- | --- | --- |
|  | Less income |  |
|  | Fewer opportunities for employment |  |
|  | Working hours have increased |  |
|  | Working hours have decreased |  |
|  | Someone in the family was ill with COVID-19 |  |
|  | Someone in the family died of COVID-19 |  |
|  | Spending more time together as a family |  |
|  | Increased conflict in the household |  |
|  | More restricted movement |  |
|  | Children have stayed home from school/nursery |  |
|  | Harder to find food |  |
|  | Harder to find/ buy medicines |  |
|  | Prices in shops have increased |  |
|  | Fewer visits to medical facilities |  |
|  | I spend less social time with people outside of my family |  |
|  | Increase in fear/concern generally |  |
|  | Concerned with strangers interacting with family |  |
|  | No changes | Anchor at bottom |
| 98 | Other (specify) | Anchor at bottom |

**SECTION B: INFLUENCERS AND DECISIONS**

ASK ALL

B1. We often need to ask other people for advice, but the people we ask could be different depending on the circumstance.

I am going to ask you who, if anyone, you asked for advice the last time you experienced different situations. Please tell me all the people you asked for advice in each case. Please also say if you didn’t ask anyone for advice.

1. A problem with money- for example you did not have enough money to pay for bills
2. When you had a disagreement with your spouse/partner
3. When your baby had a fever

MULTIPLE ANSWER, DO NOT READ LIST

CODES FOR EACH SCENARIO 1-3:

| **Code Number** | **Code Label** | **Scripting / Routing Instruction** |
| --- | --- | --- |
|  | My spouse/ partner |  |
|  | My mother |  |
|  | My father |  |
|  | My daughter |  |
|  | My son |  |
|  | My mother in law |  |
|  | My father in law |  |
|  | My husband’s other wives | ONLY SHOW IF A1=4 |
|  | My brothers in law |  |
|  | My sisters in law |  |
|  | My brothers |  |
|  | My sisters |  |
|  | My grandparents in law |  |
|  | My grandmother |  |
|  | My grandfather |  |
|  | Cousins |  |
|  | Uncles |  |
|  | Aunties |  |
|  | Family friends |  |
|  | Neighbours |  |
|  | Religious leaders |  |
|  | Colleagues from work |  |
|  | Older people in the community/ elders |  |
|  | Traditional healers |  |
|  | A doctor or a nurse |  |
|  | Other healthcare workers (Community health workers, Chemists, Patent Medicine Vendors, Pharmacists) |  |
|  | Others (specify) |  |
|  | Did not ask anyone for advice | EXCLUSIVE CODE |

ASK ALL

B2. Sometimes we are able to make decisions for ourselves, and at other times we must consult others or ask for their permission before making a decision.

I am going to ask you about different situations- the last time you did these things, did you: make the decision by yourself, discuss with someone else , ask for permission or did someone else decide

SINGLE ANSWER

FOR EACH ROW SHOW:

1. I made the decision myself
2. I discussed with someone else
3. I asked for permission
4. Someone else decided/ I don’t make that decision

| **Code Number** | **Code Label** | **Scripting / Routing Instruction** |
| --- | --- | --- |
|  | Deciding what food to buy |  |
|  | Deciding to leave the house/ compound |  |
|  | Deciding what clothes to buy for myself |  |
|  | Deciding to take my child to the clinic when they were not feeling well |  |
|  | Deciding to take my child for a vaccination |  |

RANDOMISE STATEMENTS

ASK IF 3 SELECTED FOR ANY CODES AT B2

B3. Who do you usually ask permission to make these decisions?

MULTIPLE ANSWER

COLUMNS: PIPE IN CODES SELECTED AS “3” (“I MUST ASK PERMISSION”)

ROWS:

DO NOT READ LIST

| **Code Number** | **Code Label** | **Scripting / Routing Instruction** |
| --- | --- | --- |
|  | My spouse/ partner |  |
|  | My mother |  |
|  | My father |  |
|  | My mother in law |  |
|  | My father in law |  |
|  | My husband’s other wives | ONLY SHOW IF A1=4 |
|  | My brothers in law |  |
|  | My sisters in law |  |
|  | My brothers |  |
|  | My sisters |  |
|  | My grandparents in law |  |
|  | My grandmother |  |
|  | My grandfather |  |
|  | Cousins |  |
|  | Uncles |  |
|  | Aunties |  |
|  | Family friends |  |
|  | Neighbours |  |
|  | Religious leaders |  |
|  | Others (specify) |  |

ASK ALL

B4. Some tasks within a household are seen as the responsibility of different people.

I will read to you some tasks- please tell me if you think it is the man’s responsibility, the woman’s responsibility, or if the responsibiity is shared.

SINGLE ANSWER

COLUMNS:

1. The woman’s responsibility
2. The man’s responsibility
3. It is shared

ROWS:

| **Code Number** | **Code Label** | **Scripting / Routing Instruction** |
| --- | --- | --- |
|  | Planning what food to buy for the household |  |
|  | Taking children to the clinic if they are unwell |  |
|  | Making money for the household |  |
|  | Taking children for vaccinations |  |

RANDOMISE STATEMENTS

ASK ALL

B5. I am going to read you a series of statements **about families in your community**. Please indicate how much you agree or disagree with each statement, by saying a number from 1 to 5, where 1 means ‘Strongly disagree’, 2 means ‘Slightly disagree’, 3 means ‘Neither agree nor disagree’, 4 means ‘Slightly agree’ and 5 means ‘Strongly agree’

SINGLE ANSWER PER ROW//ADD ‘DON’T KNOW/ NOT APPLICABLE’ OPTION FOR EACH ROW

| **Strongly Disagree** | **Slightly disagree** | **Neither Agree**  **Nor Disagree** | **Slightly agree** | **Strongly agree** |
| --- | --- | --- | --- | --- |
| 1 | 2 | 3 | 4 | 5 |

DOWN SIDE OF GRID

| **Code Number** | **Code Label** | **Scripting / Routing Instruction** |
| --- | --- | --- |
|  | When a man makes a decision, no one in the family should question it |  |
|  | A woman can make decisions about her children without asking her husband |  |
|  | I am worried about being blamed if I make a decision for my baby/ child and something goes wrong |  |
|  | I listen to my mother-in-law’s advice on family matters |  |
|  | I listen to my mother’s advice on family matters |  |
|  | Disagreements between a husband and wife are private and should not be talked about outside the home |  |
|  | I listen to religious leaders’ advice on family matters |  |
|  | Women should earn money to contribute to household income |  |
|  | A man should watch over his wife to make sure she does the right things |  |

RANDOMISE STATEMENTS

**SECTION C: BELIEFS, COMMUNITY, TRADITIONS AND RELIGION**

I would like to continue with a few questions about your beliefs, community, your traditions and your religion

ASK ALL

C1.

Please tell me how much you agree or disagree with each statement by saying a number from 1 to 5, where 1 means ‘Strongly Disagree’, 2 means slightly disagree, 3 means neither agree nor disagree, 4 means slightly agree and 5 means strongly agree

SINGLE ANSWER PER ROW

| **Strongly Disagree** | **Slightly disagree** | **Neither Agree**  **Nor Disagree** | **Slightly agree** | **Strongly agree** |
| --- | --- | --- | --- | --- |
| 1 | 2 | 3 | 4 | 5 |

DOWN SIDE OF GRID //ADD ‘DON’T KNOW/ NOT APPLICABLE’ OPTION FOR EACH ROW

| **Code Number** | **Code Label** | **Theme** |
| --- | --- | --- |
|  | Giving their child a good education should be the main priority for a parent | EDUCATION |
|  | Traditional/ herbal medicine is more effective than Western medicine | TRADITION |
|  | Traditional practices are outdated | TRADITION |
|  | I trust the government to act in my best interests | GOVERNMENT/ AUTHORITY |
|  | It is usually easy to move around my community | TRANSPORT BARRIERS |
|  | Travelling abroad is a sign of success | SUCCESS |
|  | It is good to show how successful you are in the community | SUCCESS |
|  | We have enough money to meet my family’s basic needs | FINANCES |
|  | To be respected in the community you need to have a lot of money | FINANCES/ RESPECT |
|  | To be respected in the community you need to be married | RESPECT/ MARRIAGE |

RANDOMISE STATEMENTS

ASK ALL

C2. Please tell me how much you agree or disagree with each statement regarding **the people in** **your community**, by saying a number from 1 to 5, where 1 means ‘Strongly Disagree’, 2 means slightly disagree, 3 means neither agree nor disagree, 4 means slightly agree and 5 means strongly agree

SINGLE ANSWER PER ROW

| **Strongly Disagree** | **Slightly disagree** | **Neither Agree**  **Nor Disagree** | **Slightly agree** | **Strongly agree** |
| --- | --- | --- | --- | --- |
| 1 | 2 | 3 | 4 | 5 |

DOWN SIDE OF GRID //ADD ‘DON’T KNOW/ NOT APPLICABLE’ OPTION FOR EACH ROW

| **Code Number** | **Code Label** | **Scripting / Routing Instruction** |
| --- | --- | --- |
|  | I trust the people in my community |  |
|  | My children are safe growing up in this community |  |
|  | I know that my neighbours will look out for me and protect me |  |
|  | I gather frequently with my neighbours/ people from the community |  |
|  | It's important what others in my community think of the decisions I make |  |
|  | People gossip about others in this community |  |
|  | I don’t want to be seen as different from my community |  |
|  | I like to wait until others have tried something before I do it |  |

RANDOMISE STATEMENTS

C3.a. I am interested in understanding how much you trust different groups of people in your community.

SINGLE ANSWER PER ROW

| **Don’t trust at all** | **Trust a little** | **Trust a lot** | **Don’t know/ not applicable** |
| --- | --- | --- | --- |
| 1 | 2 | 3 |  |

DOWN SIDE OF GRID. ADD ‘DON’T KNOW/ NOT APPLICABLE’ OPTION FOR EACH ROW

| **Code Number** | **Code Label** | **Scripting / Routing Instruction** |
| --- | --- | --- |
|  | Older people/ community elders |  |
|  | Religious leaders |  |
|  | Doctors and nurses (Healthcare workers) |  |
|  | Political leaders (e.g. Ward councillor) |  |
|  | Traditional leaders (e.g. chiefs) |  |
|  | Pharmacists |  |
|  | Your neighbours |  |
|  | Your friends |  |

RANDOMISE LIST

ASK ALL

C4. What is your religion?

SINGLE ANSWER

| **Code Number** | **Code Label** | **Scripting / Routing Instruction** |
| --- | --- | --- |
|  | Muslim |  |
|  | Christian |  |
|  | Traditional beliefs |  |
|  | Other religion |  |
|  | No religion |  |
|  | Prefer not to say |  |

ASK ALL

C5. Please indicate how much you agree or disagree with each statement about **religion**, by saying a number from 1 to 5where 1 means ‘Strongly Disagree’, 2 means slightly disagree, 3 means neither agree nor disagree, 4 means slightly agree and 5 means strongly agree

SINGLE ANSWER PER ROW// ADD ‘DON’T KNOW/ NOT APPLICABLE’ OPTION FOR EACH ROW

| **Strongly Disagree** | **Slightly disagree** | **Neither Agree**  **Nor Disagree** | **Slightly agree** | **Strongly agree** |
| --- | --- | --- | --- | --- |
| 1 | 2 | 3 | 4 | 5 |

DOWN SIDE OF GRID

| **Code Number** | **Code Label** | **Scripting / Routing Instruction** |
| --- | --- | --- |
|  | My religious faith protects me and my family from harm |  |
|  | My religious faith heals me and my family from illnesses |  |
|  | God is the only protection needed against harm |  |
|  | My religious faith guides decisions in my life |  |

RANDOMISE STATEMENTS

**SECTION D: PROTECTION AND HEALTH SEEKING**

We would like to talk now about health and protection

ASK ALL

D1. Which of the following types of health facilities have you visited in the last 2 years?

MULTIPLE ANSWER

NIGERIA:

| **Code Number** | **Code Label** | **Scripting / Routing Instruction** |
| --- | --- | --- |
|  | Government hospitals |  |
|  | Private hospitals |  |
|  | A public clinic / health centre/ health post |  |
|  | A private clinic/ health centre |  |
|  | Pharmacies |  |
|  | Chemists/PMVs |  |
|  | Other (specify) |  |
|  | None | UNIQUE CODE |

UGANDA:

| **Code Number** | **Code Label** | **Scripting / Routing Instruction** |
| --- | --- | --- |
|  | Health Centre II |  |
|  | Health Centre III |  |
|  | Health Centre IV |  |
|  | Private clinic/ hospital |  |
|  | Public hospital |  |
|  | Pharmacies |  |
|  | Drug stores |  |
|  | Other (specify) |  |
|  | None | UNIQUE CODE |

ASK ALL, EXCEPT D1=8 NIGERIA OR D1=9 UGANDA

D2. And for what purposes do you visit these health facilities?

MULTIPLE ANSWER, DO NOT READ LIST

SHOW THOSE OPTIONS SELECTED AT D1

| **Code Number** | **Code Label** | **Scripting / Routing Instruction** |
| --- | --- | --- |
|  | For ante-natal visits |  |
|  | To give birth |  |
|  | For vaccinations for my child/ children |  |
|  | For surgery/ operations |  |
|  | Treatment when I am feeling unwell |  |
|  | Treatment when my child/ children are feeling unwell |  |
|  | Treatment when someone else in my family is feeling unwell (e.g. husband, mother) |  |
|  | Routine visits to manage an ongoing condition (for you/ family member) |  |
|  | To monitor my child’s/children’s growth |  |
|  | To get a test taken (blood, urine, nasal) |  |
|  | To pick up/buy medication |  |
|  | Family planning |  |
|  | Other (specify) |  |

ASK IF S7B=1

D3. During your last pregnancy, did you meet with a health professional about your pregnancy (apart from when giving birth)? SINGLE ANSWER

| **Code Number** | **Code Label** | **Scripting / Routing Instruction** |
| --- | --- | --- |
|  | Yes |  |
|  | No |  |

ASK ALL

D4. Please tell me how much you agree or disagree with each statement about the **healthcare** **system** **and facilities** by saying a number from 1 to 5, where 1 means ‘Strongly Disagree’, 2 means slightly disagree, 3 means neither agree nor disagree, 4 means slightly agree and 5 means strongly agree

SINGLE ANSWER PER ROW

| **Strongly Disagree** | **Slightly disagree** | **Neither Agree**  **Nor Disagree** | **Slightly agree** | **Strongly agree** |
| --- | --- | --- | --- | --- |
| 1 | 2 | 3 | 4 | 5 |

DOWN SIDE OF GRID. ADD ‘DON’T KNOW/ NOT APPLICABLE’ OPTION FOR EACH ROW

| **Code Number** | **Code Label** | **Scripting / Routing Instruction** |
| --- | --- | --- |
|  | The healthcare facilities/ outreach post are too far away |  |
|  | The healthcare facilities are not clean |  |
|  | The health workers don’t treat me well |  |
|  | The waiting time is too long |  |
|  | It's too risky to visit a healthcare facility right now |  |
|  | Outreach sessions (when people come to the area to give health services) are unreliable |  |
|  | The healthcare facility is too expensive |  |

RANDOMISE STATEMENTS

ASK ALL

D5. What do you think children need to be protected from?

MULTICODE. DO NOT READ OUT LIST

DOWN SIDE OF GRID

| **Code Number** | **Code Label** | **Scripting / Routing Instruction** |
| --- | --- | --- |
|  | Diseases and infections |  |
|  | People in the community who might physically hurt them |  |
|  | Kidnappers |  |
|  | Germs |  |
|  | Bad influences on their behaviour |  |
|  | Sexual abuse |  |
|  | Sharp objects |  |
|  | Cold weather |  |
|  | Toxic/ harmful products (e.g. bleach) |  |
|  | Harmful/evil spirits |  |
|  | Dangerous animals |  |
|  | Pollution in the air |  |
|  | Illiteracy |  |
|  | Others (specify) |  |

ASK ALL

D6. How do you protect your children from these things?

MULTICODE, DO NOT READ LIST

| **Code Number** | **Code Label** | **Scripting / Routing Instruction** |
| --- | --- | --- |
|  | I take them to school |  |
|  | I bathe them frequently |  |
|  | I pray for them regularly |  |
|  | I watch the company they keep and who they play with |  |
|  | I vaccinate them |  |
|  | I take them to clinics and hospitals |  |
|  | I clean the house |  |
|  | I use herbs or traditional medicine |  |
|  | I give them nutritious food |  |
|  | I keep dangerous substances or objects or animals away from them |  |
|  | I breastfeed them |  |
|  | I keep them out of the sun |  |
|  | I dress them appropriately |  |
|  | I keep them away from sick people |  |
|  | I massage or moisturise them |  |
|  | I make them sleep under mosquito nets |  |
|  | I pierce their ears |  |
|  | Others (specify) |  |

**SECTION E: VACCINE EXPERIENCE, ATTITUDE TOWARDS VACCINES AND NORMATIVE BELIEFS**

ASK ALL

E1. Have you received any vaccinations?

MULTICODE ANSWER

| **Code Number** | **Code Label** | **Scripting / Routing Instruction** |
| --- | --- | --- |
|  | Yes, as a child |  |
|  | Yes, as an adult |  |
|  | No |  |
|  | Not sure / Don’t know |  |

ASK ALL

E2. In the last 2 years, who, if anyone, have you talked to about vaccinations?

MULTIPLE ANSWER, DO NOT READ OUT LIST

| **Code Number** | **Code Label** | **Scripting / Routing Instruction** |
| --- | --- | --- |
|  | My spouse/ partner |  |
|  | My mother |  |
|  | My father |  |
|  | My mother in law |  |
|  | My father in law |  |
|  | My husband’s other wives | ONLY SHOW IF A1=4 |
|  | My brothers in law |  |
|  | My sisters in law |  |
|  | My brothers |  |
|  | My sisters |  |
|  | My grandparents in law |  |
|  | My grandmother |  |
|  | My grandfather |  |
|  | Cousins |  |
|  | Uncles |  |
|  | Aunties |  |
|  | Friends |  |
|  | Neighbours |  |
|  | Religious leaders |  |
|  | Colleagues from work |  |
|  | Older people in the community/ elders |  |
|  | Traditional healers |  |
|  | A doctor or a nurse (health workers) |  |
|  | Others (specify) |  |
|  | Haven’t spoken to anyone | SINGLE CODE. ANCHOR AT BOTTOM |

ASK ALL

E4. Have you ever received information about vaccination through these different channels?

| **Newspaper** | **TV** | **Radio** | **Social media (E.g. Facebook, WhatsApp, Twitter )** |
| --- | --- | --- | --- |
| 1 | 2 | 3 | 4 |

SINGLE ANSWER

| **Code Number** | **Code Label** | **Scripting / Routing Instruction** |
| --- | --- | --- |
|  | Yes |  |
|  | No |  |
|  | Don’t remember |  |

ASK ALL

E5.

In your experience, for what reasons might people not fully vaccinate their child (meaning give their child all the vaccinations that are recommended by the time they are 1 years old)?

MULTICODE, DO NOT READ OUT LIST

| **Code Number** | **Code Label** | **Scripting / Routing Instruction** |
| --- | --- | --- |
|  | Concerns about the safety of vaccines and their side effects |  |
|  | Concerns about getting to the clinic or paying for the visit |  |
|  | Concerns about the clinic/ hospital or the people who work there |  |
|  | Friends, family members or community members are against vaccination |  |
|  | Because the benefits of vaccination are unclear |  |
|  | Because other things get in the way or are higher priority than vaccines |  |
|  | Because there are better alternatives to vaccination |  |
|  | Because their religion forbids vaccination |  |
|  | Concerns about how long it takes/how long they have to wait |  |
|  | Don’t know/can’t think of any reasons |  |
|  | Other (specify) |  |

ASK ALL

E6.

How much do you agree or disagree with the following statements about vaccines?

Please tell me how much you agree or disagree with each statement by saying a number from 1 to 5, where 1 means ‘Strongly Disagree’, 2 means slightly disagree, 3 means neither agree nor disagree, 4 means slightly agree and 5 means strongly agree

SINGLE ANSWER PER ROW

| **Strongly Disagree** | **Slightly disagree** | **Neither Agree**  **Nor Disagree** | **Slightly agree** | **Strongly agree** |
| --- | --- | --- | --- | --- |
| 1 | 2 | 3 | 4 | 5 |

ADD ‘DON’T KNOW/ NOT APPLICABLE’ OPTION FOR EACH ROW

| **Code Number** | **Code Label** | **Scripting / Routing Instruction** |
| --- | --- | --- |
|  | Vaccinations are too painful for children |  |
|  | FOR PARTIAL AND FULL-VACCINATORS (S11): It is not difficult for me to watch my child being vaccinated  FOR NON-VACCINATORS (S11): It would not be difficult for me to watch my child being vaccinated |  |
|  | Having many vaccinations at once is hard for children to bear |  |
|  | Vaccinations can make children infertile |  |
|  | Vaccinations cannot permanently disable a child |  |
|  | The side effects (fever, rash, pain) of vaccination are not harmful |  |
|  | It is difficult for me to manage the side effects (fever, rash, pain) of vaccination |  |
|  | I never worry that the vaccines are expired |  |
|  | Vaccines are a way for global/western countries/organisations to control us |  |
|  | I cannot vaccinate my child when they are unwell |  |

RANDOMISE STATEMENTS

ASK ALL

E7.

How much do you agree or disagree with the following statements **about the vaccination process**?

Please tell me how much you agree or disagree with each statement by saying a number from 1 to 5, where 1 means ‘Strongly Disagree’, 2 means slightly disagree, 3 means neither agree nor disagree, 4 means slightly agree and 5 means strongly agree

SINGLE ANSWER PER ROW

| **Strongly Disagree** | **Slightly disagree** | **Neither Agree**  **Nor Disagree** | **Slightly agree** | **Strongly agree** |
| --- | --- | --- | --- | --- |
| 1 | 2 | 3 | 4 | 5 |

ADD ‘DON’T KNOW/ NOT APPLICABLE’ OPTION FOR EACH ROW

| **Code Number** | **Code Label** | **Scripting / Routing Instruction** |
| --- | --- | --- |
|  | The clinic or hospital or outreach site where the vaccination takes place is far away from where I live |  |
|  | It is not expensive to travel to where the vaccination takes place |  |
|  | I don’t worry about losing income if I go to the clinic or hospital for vaccination |  |
|  | The clinics or hospitals always have vaccines available |  |
|  | It is not safe to travel to the clinic or hospital where the vaccination takes place |  |
|  | I travel a lot so it’s hard to take my child to get vaccinated |  |
|  | I’m concerned about going to the clinic and getting infected with Covid 19 |  |
|  | Vaccinations are not given if you miss the scheduled date |  |
|  | Vaccinations are available any day of the week |  |
|  | I am too busy to go to the clinic or hospital for vaccinations |  |

RANDOMISE STATEMENTS

ASK ALL

E8. Thinking about the clinic or hospital, or the people who work there. How much do you agree or disagree which the following statements?

Please tell me how much you agree or disagree with each statement by saying a number from 1 to 5, where 1 means ‘Strongly Disagree’, 2 means slightly disagree, 3 means neither agree nor disagree, 4 means slightly agree and 5 means strongly agree

SINGLE ANSWER PER ROW

| **Strongly Disagree** | **Slightly disagree** | **Neither Agree**  **Nor Disagree** | **Slightly agree** | **Strongly agree** |
| --- | --- | --- | --- | --- |
| 1 | 2 | 3 | 4 | 5 |

ADD ‘DON’T KNOW/ NOT APPLICABLE’ OPTION FOR EACH ROW

| **Code Number** | **Code Label** | **Scripting / Routing Instruction** |
| --- | --- | --- |
|  | I trust the nurses who give the vaccination |  |
|  | The staff in the hospital are rude to me |  |
|  | The clinic or hospital is dirty |  |
|  | The queues are too long at the clinic/ hospital where the vaccination takes place |  |

RANDOMISE STATEMENTS

ASK ALL

E9. Thinking about the need for vaccinations…

To what extent do you agree or disagree with the following statements? Please tell me how much you agree or disagree with each statement by saying a number from 1 to 5, where 1 means ‘Strongly Disagree’, 2 means slightly disagree, 3 means neither agree nor disagree, 4 means slightly agree and 5 means strongly agree

SINGLE ANSWER PER ROW

| **Strongly Disagree** | **Slightly disagree** | **Neither Agree**  **Nor Disagree** | **Slightly agree** | **Strongly agree** |
| --- | --- | --- | --- | --- |
| 1 | 2 | 3 | 4 | 5 |

ADD ‘DON’T KNOW/ NOT APPLICABLE’ OPTION FOR EACH ROW

| **Code Number** | **Code Label** | **Scripting / Routing Instruction** |
| --- | --- | --- |
|  | There are no benefits to vaccination |  |
|  | I understand what vaccines do |  |
|  | I believe that vaccines are effective |  |
|  | Children who have not had vaccinations are usually healthy |  |
|  | There are other ways I can protect my child from disease |  |
|  | I trust those who come into the community to do campaign vaccination |  |

RANDOMISE STATEMENTS

ASK ALL

E10. Looking at the following list of things done for children, please put these things in order of importance from highest to lowest; where 1 is the most important and 5 the least important.

READ OUT ALL OPTIONS TO RESPONDENT BEFORE ASKING THEM TO RANK. WRITE IN NUMBER 1 -5 BASED ON RESPONDENT’S ANSWER. USE SHOW CARD IF NEEDED/APPROPRIATE

| **Code Number** | **Code Label** | **Scripting / Routing Instruction** |
| --- | --- | --- |
|  | Always breastfeeding a baby |  |
|  | Paying school fees on time |  |
|  | Taking children for all vaccinations |  |
|  | Making sure a child always sleeps under a mosquito net |  |
|  | Being watchful of who a child plays with |  |

ONLY NUMBER 1,2,3,4,5 TO BE ACCEPTED. MUST BE DIFFERENT NUMBER PER OPTION

ASK ALL WHO SELECT 4 OR 5 AT CODE 5 AT E9

E11. Which of the following, if any, do you believe are better alternatives to vaccination?

MULTICODE

| **Code Number** | **Code Label** | **Scripting / Routing Instruction** |
| --- | --- | --- |
|  | God’s protection/ prayer |  |
|  | Traditional/ herbal remedies |  |
|  | Maintaining a clean house |  |
|  | Bathing the child regularly |  |
|  | Breastfeeding |  |
|  | Keeping the child warm |  |
|  | None of the above- there are no better alternatives |  |
|  | Other (specify) |  |

ASK ALL

E12. What are the reasons why people in your community/ people you know of **DO** want to take their children for vaccination?

MULTICODE, DO NOT READ LIST

| **Code Number** | **Code Label** | **Scripting / Routing Instruction** |
| --- | --- | --- |
|  | Vaccines prevent disease |  |
|  | Vaccines reduce the severity of disease |  |
|  | Vaccination is convenient/ easy to do |  |
|  | Family supports vaccination |  |
|  | Everyone in the community vaccinates their children |  |
|  | Being vaccinated may open up opportunities later in life (e.g. education, foreign travel) |  |
|  | If they see how children can be protected by vaccination |  |
|  | Religious leaders are supportive of vaccination |  |
|  | Trust that the government knows what is right for children |  |
|  | The service provided by clinics and hospitals is good |  |
|  | Afraid of punishment if children are not vaccinated |  |
|  | So their children can go to school |  |
|  | It’s part of being a good parent |  |
|  | None/ no reasons |  |
|  | Don’t know |  |
|  | Other (specify) |  |

ASK ALL

E13. What, if anything, would make vaccination easier for you?

MULTICODE, DO NOT READ OUT LIST

| **Code Number** | **Code Label** | **Scripting / Routing Instruction** |
| --- | --- | --- |
|  | Providing vaccinations at a community meeting place |  |
|  | Providing vaccinations in your home |  |
|  | Providing vaccinations in your child’s school/nursery |  |
|  | Providing vaccinations with other health services (e.g. with family planning services, ante natal care) |  |
|  | Health workers more friendly/informed |  |
|  | Less disruption from side effects after vaccination |  |
|  | Fewer injections during a single visit |  |
|  | Don’t know |  |
|  | Other (specify) |  |

ASK ALL

E14. How much do you agree with the following statements about vaccination?

Please tell me how much you agree or disagree with each statement by saying a number from 1 to 5, where 1 means ‘Strongly Disagree’, 2 means slightly disagree, 3 means neither agree nor disagree, 4 means slightly agree and 5 means strongly agree

SINGLE ANSWER PER ROW

| **Strongly Disagree** | **Slightly disagree** | **Neither Agree**  **Nor Disagree** | **Slightly agree** | **Strongly agree** |
| --- | --- | --- | --- | --- |
| 1 | 2 | 3 | 4 | 5 |

DOWN SIDE OF GRID // ADD ‘DON’T KNOW/ NOT APPLICABLE’ OPTION FOR EACH ROW

| **Code Number** | **Code Label** | **Scripting / Routing Instruction** |
| --- | --- | --- |
|  | Vaccination stops children from getting some illnesses |  |
|  | Vaccination is convenient/ easy to do |  |
|  | My husband/ partner helped/ ensured that my child was vaccinated |  |
|  | My mother/ mother-in-law  helped/ ensured that my child was vaccinated |  |
|  | If a vaccinated child gets sick, it will be less serious than if a non-vaccinated child gets sick |  |
|  | It is normal in this community to vaccinate your children |  |
|  | Religious leaders are supportive of vaccination |  |
|  | I trust that the government knows what is right for children |  |
|  | I am afraid of punishment if my child is not vaccinated |  |

RANDOMISE

ASK PARTIALLY VACCINATED (S11 – NUMBER OF CODES SELECTED RANGE FROM 1-14 **NIGERIA**); S11 – NUMBER OF CODES SELECTED RANGE FROM 1-15 (**UGANDA**) IF CHILD 1 YEAR

S11 – NUMBER OF CODES SELECTED RANGE FROM 1-13 (**UGANDA**) IF CHILD OLDER THAN 1 YEAR

AND NOT VACCINATED (S11 –ALL CODES ARE 0) OR (S8=2) **NIGERIA ;** (S11 –ALL CODES ARE 0) OR (S8=2) **UGANDA**

E15.

IF PARTIALLY VACCINATED: What, if anything, would have allowed you to ensure that your child finished the vaccination course?

IF NOT VACCINATED: What, if anything, would have encouraged you to take your child for vaccination?

MULTICODE- DO NOT READ OUT LIST

| **Code Number** | **Code Label** | **Scripting / Routing Instruction** |
| --- | --- | --- |
|  | More information about vaccines and what they do | PERSUASION THAT VACCINES ARE WORTH IT |
|  | More reassurance on the safety of vaccines | PERSUASION THAT VACCINES ARE WORTH IT |
|  | More advice on how to deal with side effects after vaccination | PERSUASION THAT VACCINES ARE WORTH IT |
|  | Help with getting to the clinic or outreach session | ACCESS AND OPPORTUNITY |
|  | Friendly and skilled staff (e.g. nurses) | ACCESS AND OPPORTUNITY |
|  | No long queues/waiting | ACCESS AND OPPORTUNITY |
|  | More support from my husband | SUPPORT FROM NETWORK |
|  | More support from other members of my family | SUPPORT FROM NETWORK |
|  | Help with basic necessities to ensure I can make time for vaccinations | SUPPORT FROM NETWORK |
|  | Better reminders about when to return for next vaccinations |  |
|  | More convenient services (when and where provided) |  |
|  | Others |  |
|  | Nothing |  |

ASK ALL

E16.

Who or where would you go to if you wanted information about vaccines?

MULTICODE, DO NOT READ OUT LIST

| **Code Number** | **Code Label** | **Scripting / Routing Instruction** |
| --- | --- | --- |
|  | Your spouse/ partner |  |
|  | Your mother |  |
|  | Your mother-in-law |  |
|  | Elders in the community |  |
|  | Religious leaders |  |
|  | Traditional leaders |  |
|  | Traditional medical practitioners |  |
|  | Doctors or nurses (Health workers) |  |
|  | Representatives of the government |  |
|  | NGO representatives |  |
|  | Teachers |  |
|  | Pharmacists / PMVs/Chemists |  |
|  | Friends |  |
|  | Singers/ musicians/ athletes |  |
|  | Social media (e.g. Instagram, Facebook) |  |
|  | Websites online |  |
|  | Radio/ TV/ newspapers |  |
|  | Other (specify) |  |
|  | No-one |  |

That is the end of our questions, thank you very much for your time and for telling us your opinions

FOR MODERATOR

E17.

Do you feel that this respondent was a non-vaccinator?

SINGLE CODE

| **Code Number** | **Code Label** | **Scripting / Routing Instruction** |
| --- | --- | --- |
|  | Yes (please specify- write comments) |  |
|  | No |  |

**[END OF SURVEY]**

# Guinea

Ipsos Healthcare

3 Thomas More Square, London, E1W 1YW, UK

Tel: + 44 20 3059 5000 Fax: +44 20 3059 4998

Internal client use only

**Vaccines Demand
19-024660-01
Screener and Questionnaire**

**Sample quotas:**

|  | Guinea |
| --- | --- |
| **Caregivers** | 1000 |

**Methodology: Face to face questionnaire**

**Interview length: 40 minutes**

INTRODUCTION

I now need to read you a statement to let you know about your rights and how you will be protected during and after this discussion. It may be a little long but is important so please be patient with me

GUINEA

I am an interviewer working for a research company called Ciblage doing research for a health organisation.

This research study aims to understand peoples’ experiences and thoughts on their community and health so that health services can be made better.

If you agree to take part, I, as a trained interviewer, will spend around 40 minutes with you and ask you questions about your opinions.

This research is completely voluntary and you are free to withdraw from the study at any time, including during the research or after it has taken place. If you choose to withdraw, nothing bad will happen.

To thank you for your time, participation and any inconvenience you will receive a small token of appreciation.

In addition to asking about what you think and do, we will also collect your name, telephone number and address so that we can contact you if needed. You may be recontacted up to 4 weeks after this interview.

All information about who you are and where you live will be kept on a secure computer which will not be available to others and will be kept confidential to the extent possible by law. The only people that will see your responses will be those doing the research or those checking that the research was done properly, and they are required to keep all details confidential.

Your answers will be stored securely on a computer and used for research purposes in ways that will not reveal who you are. All future use of the stored data will come after a Research Ethics Committee (a group who oversee if research is done properly) has approved its use.

The most significant risk would be that someone would find out that we spoke to you but we have put things in place to ensure that this does not happen, such as storing your details on a password protected computer and destroying them soon after this study.

We can do this interview wherever you want and you can refuse to answer any of the questions or stop the interview at any stage.

This study may be extremely helpful in that it will provide information to improve health services in the country.

If you would like to receive feedback on our study, we will record your phone number on a separate sheet of paper and can send you the results of the study when it is completed. This information will also be stored securely and only viewed by those who are required to see it.

This research has been approved by the Comite d'éthique recherche en santé. If you have any complaints about ethical aspects of the research or feel that you have been harmed in any way by participating in this study, please contact +224 622 25 31 27

If you have any questions about the research, you may call the project leader Mamadi Cissé ((+224) 620 53 99 53)

| The research has been explained to me and I have been given a full explanation about the possible uses of any personally identifying information, either by reading this form or having it read to me | | YES □  NO □ **TERMINATE** |
| --- | --- | --- |
| I consent to participating in this research | | YES □  NO □ **TERMINATE** |
| Are you happy for Ipsos to re-contact you regarding this research? | | YES □ NO □ |
| Print full name |  | |
| Signature |  | |
| Date |  | |
| Telephone number |  | |
| Email address | **OPTIONAL** | |

**SCREENER**

I will start with a short set of questions to make sure that you’re right for this survey.

ASK ALL

S1. INTERVIEWER RECORD: COUNTRY

SINGLE ANSWER

| **Code Number** | **Code Label** | **Scripting / Routing Instruction** |
| --- | --- | --- |
|  | Guinea |  |
|  |  |  |

S2a.Which ethnic group do you belong to- ethnicity or group do you identify most with?

| **Code Number** | **Code Label** | **Scripting / Routing Instruction** |
| --- | --- | --- |
|  | Malinka | SHOW IF S1=1 |
|  | Soussou | SHOW IF S1=1 |
|  | Poulard | SHOW IF S1=1 |
|  | Kissien | SHOW IF S1=1 |
|  | Guerze | SHOW IF S1=1 |
|  | Toma | SHOW IF S1=1 |
|  | Other (Specify) | SHOW IF S1=1 |

ALL

S2. Which language would you prefer for the interview?

SINGLE ANSWER

o

| **Code Number** | **Code Label** | **Scripting / Routing Instruction** |
| --- | --- | --- |
|  | French |  |
|  | Malinka |  |
|  | Soussou |  |
|  | Poulard |  |
|  | Kissien |  |
|  | Guerze |  |
|  | Toma |  |
|  | Other (Specify) |  |

ASK ALL

S3. INTERVIEWER RECORD REGION

SINGLE ANSWER

| **Code Number** | **Code Label** | **Scripting / Routing Instruction** |
| --- | --- | --- |
|  | Mamou |  |
|  | Conakry |  |
|  | Kankan |  |
|  | N’Zérékoré |  |
|  | Boké |  |
|  |  |  |
|  | Other | TERMINATE |

ASK ALL

S3a. INTERVIEWER RECORD DISTRICT

SINGLE ANSWER

| **Code Number** | **Code Label** | **Scripting / Routing Instruction** |
| --- | --- | --- |
|  | INSERT FROM APPENDIX A: DROP DOWN MENU BASED ON REGION SELECTED AT S3 |  |
|  |  |  |
|  |  |  |
|  |  |  |
|  |  |  |
|  |  |  |
|  | Other | TERMINATE |

ASK ALL

S3b. INTERVIEWER RECORD ENUMERATION AREA

SINGLE ANSWER

| **Code Number** | **Code Label** | **Scripting / Routing Instruction** |
| --- | --- | --- |
|  | INSERT FROM APPENDIX A: DROP DOWN MENU BASED ON DISTRICT SELECTED AT S3a |  |
|  |  |  |
|  |  |  |
|  |  |  |
|  |  |  |
|  |  |  |
|  | Other | TERMINATE |

ASK ALL

S4. INTERVIEWR: RECORD LOCATION

SINGLE ANSWER

| **Code Number** | **Code Label** | **Scripting / Routing Instruction**  **QUOTAS** |
| --- | --- | --- |
|  | Urban |  |
|  |  |  |
|  | Rural |  |

ASK ALL

S5. How old were you at your last birthday?

SINGLE ANSWER ONLY

| **Code Number** | **Code Label** | **Scripting / Routing Instruction** |
| --- | --- | --- |
|  | Less than 18 | TERMINATE |
|  | 18-24 |  |
|  | 25-29 |  |
|  | 30-34 |  |
|  | 35-39 |  |
|  | 40-44 |  |
|  | 45-49 |  |
|  | 50-65 |  |
|  | 65+ |  |

IF PARTICIPANT DOESN’T WANT TO SHARE THIS INFORMATION, PLEASE TERMINATE. WE CANNOT INTERVIEW PEOPLE UNDER 18

ASK ALL

S6. How many children, of any age, do you have? This could be biological children, or children you have adopted/ look after permanently.

NUMERIC ENTRY

|  | *Child/ children* |
| --- | --- |

MIN – 0; MAX – 30

TO TERMINATE IF 0

ASK ALL

S6a. How many children do you have between ages 12 – 36months (1-3 years)?

NUMERIC ENTRY

|  | *Child/ children* |
| --- | --- |

MIN – 0; MAX – 30. SHOULD NOT BE GREATER THAN NUMBER AT S6

TO TERMINATE IF 0

ASK IF S6A >1

S6b. Of your children between 12-36 months (1-3 years), what are their names and ages?

| **Code Number** | **Code Label A)** | **B)** | **C) Name** | **Scripting / Routing Instruction** |
| --- | --- | --- | --- | --- |
|  | ____xx__years | OR __ xx ____months |  |  |

INSERT NUMBER OF ROWS = NUMBER AT S6a

SHOW IF S6b ASKED:

Today we will be talking about [INSERT NAME OF YOUNGEST CHILD AT S6B]. So please think about this child for all future questions

PN: IF MORE THAN ONE CHILD ARE THE SAME AGE AND THE YOUNGEST BETWEEN 12 AND 36 MONTHS, PROGRAMME SHOULD RANDOMLY SELECT ONE

ASK ALL

S7. Are you the primary guardian or person responsible for providing day to day care to [INSERT NAME OF YOUNGEST CHILD AT S6B ] ?

SINGLE ANSWER

| **Code Number** | **Code Label** | **Scripting / Routing Instruction** |
| --- | --- | --- |
|  | Yes | TO CONTINUE |
|  | No | TO TERMINATE |

TO TERMINATE IF CODE 2 SELECTED

ASK ALL

S7b. What is your relation with [INSERT NAME OF YOUNGEST CHILD AT S6B]?

SINGLE ANSWER, DON’T READ OUT LIST

| **Code Number** | **Code Label** | **Scripting / Routing Instruction** |
| --- | --- | --- |
|  | Biological mother |  |
|  | Stepmother |  |
|  | Aunt |  |
|  | Grandmother |  |
|  | Biological father |  |
|  | Stepfather |  |
|  | Other specify |  |

S7c. What gender is [INSERT NAME OF YOUNGEST CHILD AT S6B]?

SINGLE ANSWER

| **Code Number** | **Code Label** | **Scripting / Routing Instruction**  **QUOTAS** |
| --- | --- | --- |
|  | Male | CONTINUE |
|  | Female | CONTINUE |
|  | Prefer not to say | CONTINUE |

ASK ALL

S8. Thinking about [INSERT NAME OF YOUNGEST CHILD AT S6B ], did he/she receive any vaccines before the age of 1? By vaccine I mean something that is given to people, when they are not ill, to try to stop them from getting certain diseases. They are usually given as an injection, but sometimes given by mouth

SINGLE ANSWER

| **Code Number** | **Code Label** | **Scripting / Routing Instruction** |
| --- | --- | --- |
|  | Yes | TO CONTINUE |
|  | No | TO CONTINUE |
|  | Don’t know/ Don’t remember | TO CONTINUE |

ASK IF S8= 1 0R 3

S9. Do you have a card/book for [INSERT NAME OF YOUNGEST CHILD AT S6B ] that records which immunisations he/she has had?

SINGLE ANSWER

| **Code Number** | **Code Label** | **Scripting / Routing Instruction** |
| --- | --- | --- |
|  | Yes | TO CONTINUE |
|  | No | TO TERMINATE IF ANSWERED DON’T KNOW AT QS8 |

ASK IF [S8=1] OR [S8=3 AND S9=1] (VACCINATED)

S10. Please think about the times when [INSERT NAME OF YOUNGEST CHILD AT S6B ] was given vaccines. At any of these times…

SINGLE ANSWER PER QUESTION

PLEASE ADD A YES/NO OPTION FOR EACH ROW

| **Code Number** | **Code Label** | **Scripting / Routing Instruction/**  **Quota** |
| --- | --- | --- |
|  | …Was there a reminder from the health facility that this was the time to get that vaccination? | YES: Routine |
|  | …Did someone come to your area specifically to do the vaccinations? | YES: Outreach/ Campaign |
|  | Did the vaccination take place in a clinic/ hospital/ outreach post? | YES Routine/ campaign |
|  | Did the vaccination take place in a public place (in a tent/ public hall?) | YES Outreach/ campaign |

ASK IF [S8=1] OR [S8=3 AND S9=1] (VACCINATED)

S11. IF VACCINATION CARD AVALABLE, REFER TO VACCINATION CARD AND RECORD WHICH VACCINATIONS HAVE BEEN ADMINISTERED- ONLY IN THE FIRST 12 MONTHS (I.E. DO NOT INCLUDE VACCINES WHICH WERE GIVEN AFTER 1 YEAR OLD). IF CARD NOT AVAILABLE, READ OUT THE LIST

MULTICODE

Which vaccinations has your child had?

|  | **At birth** | |
| --- | --- | --- |
| **1** | **BCG** (birth) (usually given in the left upper arm, which usually causes a scar) |  |
|  |  |  |
| **2** | **Oral polio**  (birth) (drops in the mouth) |  |
|  | **At 6 weeks:** | |
| **3** | **Oral polio**  (drops in the mouth) |  |
| **4** | **Pentavalent** (diphtheria, tetanus, pertussis, Haemophilus influenzae B and Hepatitis B (6 weeks) (injection in their thigh) |  |
|  |  |  |
|  |  |  |
|  | **At 10 weeks:** | |
| **5** | **Oral polio**  (drops in the mouth) |  |
| **6** | **Pentavalent** (diphtheria, tetanus, pertussis, Haemophilus influenzae B and Hepatitis B (10 weeks) (injection in their thigh) |  |
|  |  |  |
|  |  |  |
|  | **At 14 weeks:** | |
| **7** | **Oral polio**  (drops in the mouth) |  |
| **8** | **Pentavalent** (diphtheria, tetanus, pertussis, Haemophilus influenzae B and Hepatitis B (14 weeks) (injection in their thigh) |  |
|  |  |  |
| 9 | IPV (**inactivated polio** vaccine) (14 weeks)  (injection usually given on thigh) |  |
|  | **At 9 months:** | |
| **10** | **Measles** (9 months) (injection in left upper arm) |  |
| **11** | **Yellow Fever** (9 months) (injection in right upper arm) |  |

VACCINATION STATUS VARIABLE// QUOTA

GUINEA

FOR THIS VARIABLE EXCLUDE CODE 11 YELLOW FEVER

| NOT VACCINATED | *S11 –ALL CODES ARE 0*  OR  S8=2 |
| --- | --- |
| PARTIALLY VACCINATED | S11 – **NUMBER** OF CODES SELECTED RANGE FROM 1-9 |
| FULLY VACCINATED | S11– ALL 10 CODES SELECTED - EXCLUDING YELLOW FEVER |

ASK IF [S8=1] OR [S8=3 AND S9=1] (VACCINATED)

S12. Did your child go to private health facilities, public facilities, or a mixture of both to get vaccinated? SINGLE ANSWER

| **Code Number** | **Code Label** | **Scripting / Routing Instruction** |
| --- | --- | --- |
|  | All vaccines in private clinic/hospital | TO TERMINATE |
|  | Some in private clinic and some in public clinic/hospital/health centre | TO CONTINUE |
|  | All vaccines in public clinic/hospital/health centre or outreach session | TO CONTINUE |
|  | Don’t know/ prefer not to answer | CLOSE |

ASK ALL

S13. What is the highest level of formal education you have completed?

SINGLE ANSWER

| **Code Number** | **Code Label** | **Scripting / Routing Instruction** |
| --- | --- | --- |
|  | No formal education |  |
|  | Islamic education |  |
|  | Some primary (not finished) |  |
|  | Primary completed |  |
|  | Some secondary (not finished) |  |
|  | Secondary completed |  |
|  | Technical College completed |  |
|  | University (Bachelors) degree completed |  |
|  | Master’s degree completed |  |
|  | PhD |  |
|  | Prefer not to answer |  |

CREATE A NEW VARIABLE TO MONITOR ‘EDUCATION LEVEL’ SOFT QUOTA

| NO FORMAL EDUCATION | *S13 –CODES 1 OR 3* |
| --- | --- |
| PRIMARY COMPLETED | S13 – CODES 4 OR 5 |
| SECONDARY COMPLETED | S13– CODE 6 |
| TERTIARY COMPLETED | S13 – CODES 7 OR 8 OR 9 OR 10 |
| PREFER NOT TO ANSWER | S13 – CODE 11 |

ASK ALL

S14. What is the typical monthly income for your household?

INTERVIEWER: WE ASSUME HERE GROSS (BEFORE TAX) HH INCOME IN CASE YOU NEED TO SPECIFY IT TO THE PARTICIPANT

GUINEA

SINGLE ANSWER

| **Code Number** | **Code Label** | **Scripting / Routing Instruction** |
| --- | --- | --- |
|  | 250,000 FG or below | **LOW- CHECK QUOTAS** |
|  | 250,001 to 1,983,626 FG | **LOW- CHECK QUOTAS** |
|  | 1,983,627 to 3,000,000 FG | **MIDDLE- CHECK QUOTAS** |
|  | 3,000,001 to 4,999,999 FG | **MIDDLE- CHECK QUOTAS** |
|  | More than 5,000,000 FG | **HIGH- CHECK QUOTAS** |
|  | Prefer not to answer |  |

**SECTION A: DEMOGRAPHICS**


Thank you for the information you have provided to us first of all, your answers show that you are able to take part in the research. To start our interview, I’m going to ask you a few questions about yourself, your family and your household

ASK ALL

A1. What is your relationship status?

SINGLE ANSWER

CODES 1, 3 AND 4 ARE UNIQUES. ONLY CODES 2 ,5 AND 6 COULD BE SELECTED SIMULTANEOUSLY

| **Code Number** | **Code Label** | **Scripting / Routing Instruction** |
| --- | --- | --- |
|  | Single | SINGLE CODE |
|  | In a relationship (not married) | MULTICODE |
|  | Married, I’m the only wife/husband | SINGLE CODE |
|  | Married, I’m NOT the only wife/husband | SHOW ONLY TO THOSE ANSWERING CODES 1-4 OR 7 AT S7b  SINGLE CODE |
|  | Separated/ Divorced | MULTICODE |
|  | Widowed | MULTICODE |

ASK IF A1= 3,4,5 OR 6

A2. At what age did you get married?

NUMERIC ENTRY

|  | *Years old* |
| --- | --- |

MIN – 0; MAX – 50

ASK ALL

A3. How many people live in your household, including yourself, all children, all family members and house help?

NUMERIC ENTRY

|  | *people* |
| --- | --- |

MIN – 2; MAX – 50

ASK IF A3=2 OR MORE

A4. Who do you live with in your household?

MULTIPLE ANSWER. DO NOT READ OUT LIST

| **Code Number** | **Code Label** | **Scripting / Routing Instruction** |
| --- | --- | --- |
|  | My spouse/ partner |  |
|  | My mother |  |
|  | My father |  |
|  | My mother in law |  |
|  | My father in law |  |
|  | My husband’s other wives | ONLY SHOW IF A1=4 |
|  | My brothers in law |  |
|  | My sisters in law |  |
|  | My brothers |  |
|  | My sisters |  |
|  | My grandparents in law |  |
|  | My grandmother |  |
|  | My grandfather |  |
|  | Cousins |  |
|  | Uncles |  |
|  | Aunties |  |
|  | Family friends |  |
|  | House help |  |
|  | Someone else’s children |  |
|  | My child(ren) |  |
|  | Lodger/ someone who rents a room |  |
|  | Others (specify) |  |

**ASK ALL**

A5. Which of the following best describes your employment status?

MULTICODE ANSWER

| **Code Number** | **Code Label** | **Scripting / Routing Instruction** |
| --- | --- | --- |
|  | Employed by someone else / a company (part-time or full time) |  |
|  | Self-employed (part-time or full time) |  |
|  | Unemployed |  |
|  | Student |  |
| 98 | Other (specify) | Anchor at bottom |

ASK IF A5=1 OR 2

A6. What is your occupation?

MULTI ANSWER, DO NOT READ OUT LIST

| **Code Number** | **Code Label** | **Scripting / Routing Instruction** |
| --- | --- | --- |
|  | Farmer (including crops and animals, hunting and fishing) |  |
|  | Mining and quarrying |  |
|  | Work in a factory |  |
|  | Construction (e.g. builder, engineer) |  |
|  | Trade (e.g. shopkeeper, works on market stall) |  |
|  | Accommodation and food services (e.g. works in hotel, restaurant, café) |  |
|  | Transport (e.g. driver) |  |
|  | Information and communication (e.g. works in television or film industry, computer programmer) |  |
|  | Finance and insurance (e.g. bank worker) |  |
|  | A profession (e.g. lawyer, accountant, architect, scientific researcher, advertising and marketing) |  |
|  | Administrator (e.g. office administrator) |  |
|  | Government employee (e.g. civil servant) |  |
|  | Education (e.g. teacher) |  |
|  | Healthcare worker (e.g. doctor, nurse, social worker) |  |
|  | Casual worker |  |
| 98 | Other (specify) | Anchor at bottom |

ASK IF A5= 1 0R 2

A7. How many hours of paid work did you do last week?

|  | *Hours* |
| --- | --- |

NUMERICAL INPUT

RANGE (0-120)

ASK ALL

A8. Which mode of transport would you most likely take to each of these places?

SINGLE ANSWER PER COLUMN

| **The nearest public clinic / health centre/ health post** |  | **The nearest hospital** |  |
| --- | --- | --- | --- |
| 1 | 2 | 3 | 4 |

DOWN SIDE OF GRID

| **Code Number** | **Code Label** | **Scripting / Routing Instruction** |
| --- | --- | --- |
|  | Walking |  |
|  | Bus |  |
|  | Moto taxi |  |
|  | Taxi (Car) |  |
|  | Car (mine/family/friend) |  |
|  | Bicycle |  |
|  | Mini bus (magbana) |  |
|  | Motor tricycle (vahidei or bonbonna) |  |
|  | Personal Motorcycle |  |
|  | Other (specify) |  |

ASK ALL

A8a. And how long would it take to reach that place by that mode of transport?

SINGLE ANSWER PER COLUMN

| **The nearest public clinic / health centre/ health post** |  | **The nearest hospital** |  |
| --- | --- | --- | --- |
| 1 | 2 | 3 | 4 |

DOWN SIDE OF GRID

| **Code Number** | **Code Label** | **Scripting / Routing Instruction** |
| --- | --- | --- |
|  | 0-15 minutes |  |
|  | 16-30 minutes |  |
|  | 31-60 minutes |  |
|  | 61-120 minutes |  |
|  | More than 120 minutes |  |
|  | Don’t know |  |

ASK ALL

A9. Since the COVID-19 pandemic started at the beginning of 2020, how, if at all, has your family’s life changed?

MULTI ANSWER, DO NOT READ OUT LIST

INTERVIEWER: PROBE IF THEY SAY, ‘NO CHANGES’ BEFORE SELECTING OPTION 18.

ASK THEM WHETHER, THEIR INCOME HAS STAYED THE SAME, THEIR LIVING ARRANGEMENT IS EXACTLY THE SAME, THEIR CHILDREN HAVE BEEN GOING TO SCHOOL/NURSERY AS EXAMPLES

| **Code Number** | **Code Label** | **Scripting / Routing Instruction** |
| --- | --- | --- |
|  | Less income |  |
|  | Fewer opportunities for employment |  |
|  | Working hours have increased |  |
|  | Working hours have decreased |  |
|  | Someone in the family was ill with COVID-19 |  |
|  | Someone in the family died of COVID-19 |  |
|  | Spending more time together as a family |  |
|  | Increased conflict in the household |  |
|  | More restricted movement |  |
|  | Children have stayed home from school/nursery |  |
|  | Harder to find food |  |
|  | Harder to find/ buy medicines |  |
|  | Prices in shops have increased |  |
|  | Fewer visits to medical facilities |  |
|  | I spend less social time with people outside of my family |  |
|  | Increase in fear/concern generally |  |
|  | Concerned with strangers interacting with family |  |
|  | Increased transport costs |  |
|  | No changes | Anchor at bottom |
| 98 | Other (specify) | Anchor at bottom |

**ASK ALL**

**A10. How comfortable would you be about getting the COVID-19 vaccine for yourself?**

| **Not comfortable at all** | **A little comfortable** | **Extremely comfortable** |
| --- | --- | --- |
| 1 | 2 | 3 |

**SECTION B: INFLUENCERS AND DECISIONS**

ASK ALL

B1. We often need to ask other people for advice, but the people we ask could be different depending on the circumstance.

I am going to ask you who, if anyone, you asked for advice the last time you experienced different situations. Please tell me all the people you asked for advice in each case. Please also say if you didn’t ask anyone for advice.

1. A problem with money- for example you did not have enough money to pay for bills
2. When you had a disagreement with your spouse/partner
3. When your baby had a fever

MULTIPLE ANSWER, DO NOT READ LIST

CODES FOR EACH SCENARIO 1-3:

| **Code Number** | **Code Label** | **Scripting / Routing Instruction** |
| --- | --- | --- |
|  | My spouse/ partner |  |
|  | My mother |  |
|  | My father |  |
|  | My daughter |  |
|  | My son |  |
|  | My mother in law |  |
|  | My father in law |  |
|  | My husband’s other wives | ONLY SHOW IF A1=4 |
|  | My brothers in law |  |
|  | My sisters in law |  |
|  | My brothers |  |
|  | My sisters |  |
|  | My grandparents in law |  |
|  | My grandmother |  |
|  | My grandfather |  |
|  | Cousins |  |
|  | Uncles |  |
|  | Aunties |  |
|  | Family friends |  |
|  | Neighbours |  |
|  | Religious leaders |  |
|  | Colleagues from work |  |
|  | Older people in the community/ elders |  |
|  | Traditional healers |  |
|  | A doctor or a nurse |  |
|  | Other healthcare workers (Community health workers, Chemists, Patent Medicine Vendors, Pharmacists) |  |
|  | Others (specify) |  |
|  | Did not ask anyone for advice | EXCLUSIVE CODE |

ASK ALL

B2. Sometimes we are able to make decisions for ourselves, and at other times we must consult others or ask for their permission before making a decision.

I am going to ask you about different situations- the last time you did these things, did you: make the decision by yourself, discuss with someone else , ask for permission or did someone else decide

SINGLE ANSWER

FOR EACH ROW SHOW:

1. I made the decision myself
2. I discussed with someone else
3. I asked for permission
4. Someone else decided/ I don’t make that decision

| **Code Number** | **Code Label** | **Scripting / Routing Instruction** |
| --- | --- | --- |
|  | Deciding what food to buy |  |
|  | Deciding to leave the house/ compound |  |
|  |  |  |
|  | Deciding to take my child to the clinic when they were not feeling well |  |
|  | Deciding to take my child for a vaccination |  |

RANDOMISE STATEMENTS

ASK IF 3 SELECTED FOR ANY CODES AT B2

B3. Who do you usually ask permission to make these decisions?

MULTIPLE ANSWER

COLUMNS: PIPE IN CODES SELECTED AS “3” (“I MUST ASK PERMISSION”)

ROWS:

DO NOT READ LIST

| **Code Number** | **Code Label** | **Scripting / Routing Instruction** |
| --- | --- | --- |
|  | My spouse/ partner |  |
|  | My mother |  |
|  | My father |  |
|  | My mother in law |  |
|  | My father in law |  |
|  | My husband’s other wives | ONLY SHOW IF A1=4 |
|  | My brothers in law |  |
|  | My sisters in law |  |
|  | My brothers |  |
|  | My sisters |  |
|  | My grandparents in law |  |
|  | My grandmother |  |
|  | My grandfather |  |
|  | Cousins |  |
|  | Uncles |  |
|  | Aunties |  |
|  | Family friends |  |
|  | Neighbours |  |
|  | Religious leaders |  |
|  | Others (specify) |  |

ASK ALL

B4. Some tasks within a household are seen as the responsibility of different people.

I will read to you some tasks- please tell me if you think it is the man’s responsibility, the woman’s responsibility, or if the responsibiity is shared.

SINGLE ANSWER

COLUMNS:

1. The woman’s responsibility
2. The man’s responsibility
3. It is shared

ROWS:

| **Code Number** | **Code Label** | **Scripting / Routing Instruction** |
| --- | --- | --- |
|  | Planning what food to buy for the household |  |
|  | Taking children to the clinic if they are unwell |  |
|  | Making money for the household |  |
|  | Taking children for vaccinations |  |

RANDOMISE STATEMENTS

ASK ALL

B5. I am going to read you a series of statements **about families in your community**. Please indicate how much you agree or disagree with each statement, by saying a number from 1 to 5, where 1 means ‘Strongly disagree’, 2 means ‘Slightly disagree’, 3 means ‘Neither agree nor disagree’, 4 means ‘Slightly agree’ and 5 means ‘Strongly agree’

SINGLE ANSWER PER ROW//ADD ‘DON’T KNOW/ NOT APPLICABLE’ OPTION FOR EACH ROW

| **Strongly Disagree** | **Slightly disagree** | **Neither Agree**  **Nor Disagree** | **Slightly agree** | **Strongly agree** |
| --- | --- | --- | --- | --- |
| 1 | 2 | 3 | 4 | 5 |

DOWN SIDE OF GRID

| **Code Number** | **Code Label** | **Scripting / Routing Instruction** |
| --- | --- | --- |
|  | When a man makes a decision, no one in the family should question it |  |
|  | A woman can make decisions about her children without asking her husband |  |
|  | I am worried about being blamed if I make a decision for my baby/ child and something goes wrong |  |
|  | I listen to my mother-in-law’s advice on family matters |  |
|  | I listen to my mother’s advice on family matters |  |
|  | Disagreements between a husband and wife are private and should not be talked about outside the home |  |
|  | I listen to religious leaders’ advice on family matters |  |
|  | Women should earn money to contribute to household income |  |
|  | A man should monitor his wife to make sure she does the right things |  |

RANDOMISE STATEMENTS

**SECTION C: BELIEFS, COMMUNITY, TRADITIONS AND RELIGION**

I would like to continue with a few questions about your beliefs, community, your traditions and your religion

ASK ALL

C1.

Please tell me how much you agree or disagree with each statement by saying a number from 1 to 5, where 1 means ‘Strongly Disagree’, 2 means slightly disagree, 3 means neither agree nor disagree, 4 means slightly agree and 5 means strongly agree

SINGLE ANSWER PER ROW

| **Strongly Disagree** | **Slightly disagree** | **Neither Agree**  **Nor Disagree** | **Slightly agree** | **Strongly agree** |
| --- | --- | --- | --- | --- |
| 1 | 2 | 3 | 4 | 5 |

DOWN SIDE OF GRID //ADD ‘DON’T KNOW/ NOT APPLICABLE’ OPTION FOR EACH ROW

| **Code Number** | **Code Label** | **Theme** |
| --- | --- | --- |
|  | Giving their child a good education should be the main priority for a parent | EDUCATION |
|  | Traditional/ herbal medicine is more effective than Western medicine | TRADITION |
|  |  | TRADITION |
|  |  | GOVERNMENT/ AUTHORITY |
|  | It is usually easy to move around my community | TRANSPORT BARRIERS |
|  | Travelling abroad is a sign of success | SUCCESS |
|  | It is good to show how successful you are in the community | SUCCESS |
|  | We have enough money to meet my family’s basic needs | FINANCES |
|  | To be respected in the community you need to have a lot of money | FINANCES/ RESPECT |
|  | To be respected in the community you need to be married | RESPECT/ MARRIAGE |

RANDOMISE STATEMENTS

ASK ALL

C2. Please tell me how much you agree or disagree with each statement regarding **the people in** **your community**, by saying a number from 1 to 5, where 1 means ‘Strongly Disagree’, 2 means slightly disagree, 3 means neither agree nor disagree, 4 means slightly agree and 5 means strongly agree

SINGLE ANSWER PER ROW

| **Strongly Disagree** | **Slightly disagree** | **Neither Agree**  **Nor Disagree** | **Slightly agree** | **Strongly agree** |
| --- | --- | --- | --- | --- |
| 1 | 2 | 3 | 4 | 5 |

DOWN SIDE OF GRID //ADD ‘DON’T KNOW/ NOT APPLICABLE’ OPTION FOR EACH ROW

| **Code Number** | **Code Label** | **Scripting / Routing Instruction** |
| --- | --- | --- |
|  | I trust the people in my community |  |
|  | My children are safe growing up in this community |  |
|  | I know that my neighbours will look out for me and protect me |  |
|  | I gather frequently with my neighbours/ people from the community |  |
|  | It's important what others in my community think of the decisions I make |  |
|  | People gossip about others in this community |  |
|  | I don’t want to be seen as different from my community |  |
|  | I like to wait until others have tried something before I do it |  |

RANDOMISE STATEMENTS

ASK ALL

C3.a. I am interested in understanding how much you trust different groups of people in your community.

SINGLE ANSWER PER ROW

| **Don’t trust at all** | **Trust a little** | **Trust a lot** | **Don’t know/ not applicable** |
| --- | --- | --- | --- |
| 1 | 2 | 3 |  |

DOWN SIDE OF GRID. ADD ‘DON’T KNOW/ NOT APPLICABLE’ OPTION FOR EACH ROW

| **Code Number** | **Code Label** | **Scripting / Routing Instruction** |
| --- | --- | --- |
|  | Older people/ community elders |  |
|  | Religious leaders |  |
|  | Doctors and nurses (Healthcare workers) |  |
|  | Political leaders (e.g. Ward councillor) |  |
|  | Traditional leaders (e.g. chiefs) |  |
|  | Pharmacists |  |
|  | Your neighbours |  |
|  | Your friends |  |

RANDOMISE LIST

ASK ALL

C4. What is your religion?

SINGLE ANSWER

| **Code Number** | **Code Label** | **Scripting / Routing Instruction** |
| --- | --- | --- |
|  | Muslim |  |
|  | Christian |  |
|  | Traditional beliefs |  |
|  | Other religion |  |
|  | No religion |  |
|  | Prefer not to say |  |

ASK ALL

C5. Please indicate how much you agree or disagree with each statement about **religion**, by saying a number from 1 to 5 where 1 means ‘Strongly Disagree’, 2 means slightly disagree, 3 means neither agree nor disagree, 4 means slightly agree and 5 means strongly agree

SINGLE ANSWER PER ROW// ADD ‘DON’T KNOW/ NOT APPLICABLE’ OPTION FOR EACH ROW

| **Strongly Disagree** | **Slightly disagree** | **Neither Agree**  **Nor Disagree** | **Slightly agree** | **Strongly agree** |
| --- | --- | --- | --- | --- |
| 1 | 2 | 3 | 4 | 5 |

DOWN SIDE OF GRID

| **Code Number** | **Code Label** | **Scripting / Routing Instruction** |
| --- | --- | --- |
|  | My religious faith protects me and my family from harm |  |
|  | My religious faith heals me and my family from illnesses |  |
|  | God is the only protection needed against harm |  |
|  | My religious faith guides decisions in my life |  |

RANDOMISE STATEMENTS

**SECTION D: PROTECTION AND HEALTH SEEKING**

We would like to talk now about health and protection

ASK ALL

D7. To what extent, if at all, are you concerned about your children getting the following diseases?

SINGLE ANSWER PER ROW// ADD ‘DON’T KNOW/ NOT APPLICABLE’ OPTION FOR EACH ROW

| **Not at all concerned** | **A little concerned** | **Very concerned** |  |
| --- | --- | --- | --- |
| 1 | 2 | 4 |  |

DOWN SIDE OF GRID

| **Code Number** | **Code Label** | **Scripting / Routing Instruction** |
| --- | --- | --- |
|  | Measles |  |
|  | Malaria |  |
|  | Ebola |  |
|  | Polio |  |
|  | Covid-19 |  |
|  |  |  |
|  |  |  |

RANDOMISE STATEMENTS

ASK IF S7B=1

D3. During your last pregnancy (or your partner’s last pregnancy), did you/they meet with a health professional about your pregnancy (apart from when giving birth)? SINGLE ANSWER

| **Code Number** | **Code Label** | **Scripting / Routing Instruction** |
| --- | --- | --- |
|  | Yes |  |
|  | No |  |

| **Code Number** | **Code Label** | **Scripting / Routing Instruction** |
| --- | --- | --- |

ASK ALL

D5. What do you think children need to be protected from?

MULTICODE. DO NOT READ OUT LIST

DOWN SIDE OF GRID

| **Code Number** | **Code Label** | **Scripting / Routing Instruction** |
| --- | --- | --- |
|  | Diseases and infections |  |
|  | People in the community who might physically hurt them |  |
|  | Kidnappers |  |
|  | Germs |  |
|  | Bad influences on their behaviour |  |
|  | Sexual abuse |  |
|  | Sharp objects |  |
|  | Cold weather |  |
|  | Toxic/ harmful products (e.g. bleach) |  |
|  | Harmful/evil spirits |  |
|  | Dangerous animals |  |
|  | Pollution in the air |  |
|  | Illiteracy |  |
|  | Covid-19 |  |
|  | Hunger/lack of food |  |
|  | Fire |  |
|  | People using witchcraft against my child |  |
|  | Others (specify) |  |

ASK ALL

D6. How do you protect your children from these things?

MULTICODE, DO NOT READ LIST

| **Code Number** | **Code Label** | **Scripting / Routing Instruction** |
| --- | --- | --- |
|  | I take them to school |  |
|  | I bathe them frequently |  |
|  | I pray for them regularly |  |
|  | I watch the company they keep and who they play with |  |
|  | I vaccinate them |  |
|  | I take them to clinics and hospitals |  |
|  | I clean the house |  |
|  | I use herbs or traditional medicine |  |
|  | I give them nutritious food |  |
|  | I keep dangerous substances or objects or animals away from them |  |
|  | I breastfeed them |  |
|  | I keep them out of the sun |  |
|  | I dress them appropriately |  |
|  | I keep them away from sick people |  |
|  | I massage or moisturise them |  |
|  | I make them sleep under mosquito nets |  |
|  | I pierce their ears |  |
|  | I advise/educate them on how to be safe |  |
|  | I boil their water before they drink it |  |
|  | Others (specify) |  |

**SECTION E: VACCINES**

ASK ALL

E1. Have you received any vaccinations? By vaccination, I mean an injection or oral drop to prevent illness, NOT an injection you received when you were ill as treatment

MULTICODE ANSWER

| **Code Number** | **Code Label** | **Scripting / Routing Instruction** |
| --- | --- | --- |
|  | Yes, as a child |  |
|  | Yes, as an adult |  |
|  | No |  |
|  | Not sure / Don’t know |  |

ASK ALL

E2. In the last 2 years, who, if anyone, have you talked to about vaccinations?

MULTIPLE ANSWER, DO NOT READ OUT LIST

| **Code Number** | **Code Label** | **Scripting / Routing Instruction** |
| --- | --- | --- |
|  | My spouse/ partner |  |
|  | My mother |  |
|  | My father |  |
|  | My mother in law |  |
|  | My father in law |  |
|  | My husband’s other wives | ONLY SHOW IF A1=4 |
|  | My brothers in law |  |
|  | My sisters in law |  |
|  | My brothers |  |
|  | My sisters |  |
|  | My grandparents in law |  |
|  | My grandmother |  |
|  | My grandfather |  |
|  | Cousins |  |
|  | Uncles |  |
|  | Aunties |  |
|  | Friends |  |
|  | Neighbours |  |
|  | Religious leaders |  |
|  | Colleagues from work |  |
|  | Older people in the community/ elders |  |
|  | Traditional healers |  |
|  | A doctor or a nurse (health workers) |  |
|  | Others (specify) |  |
|  | Haven’t spoken to anyone | SINGLE CODE. ANCHOR AT BOTTOM |

ASK ALL

E4a. How many times did you do each of the following things in the last week?

| **Reading Newspapers** | **Watching TV** | **Listening to the Radio** | **On Social media (E.g. Facebook, WhatsApp, Twitter)** |
| --- | --- | --- | --- |
| 1 | 2 | 3 | 4 |

SINGLE ANSWER

| **Code Number** | **Code Label** | **Scripting / Routing Instruction** |
| --- | --- | --- |
|  | More than once a day |  |
|  | Once a day |  |
|  | Four times or more during the week |  |
|  | Two or three times during the week |  |
|  | Once during the week |  |
|  | Did not do |  |

ASK ALL

E4 Have you ever received information about vaccination through these different channels?

| **Newspaper** | **TV** | **Radio** | **Social media (E.g. Facebook, WhatsApp, Twitter)** |
| --- | --- | --- | --- |
| 1 | 2 | 3 | 4 |

SINGLE ANSWER

| **Code Number** | **Code Label** | **Scripting / Routing Instruction** |
| --- | --- | --- |
|  | Yes |  |
|  | No |  |
|  | Don’t remember |  |

ASK ALL

E5.

In your experience, for what reasons might people not fully vaccinate their child (meaning give their child all the vaccinations that are recommended by the time they are 1 years old)?

MULTICODE, DO NOT READ OUT LIST

| **Code Number** | **Code Label** | **Scripting / Routing Instruction** |
| --- | --- | --- |
|  | Concerns about the safety of vaccines and their side effects |  |
|  | Concerns about getting to the clinic or paying for the visit |  |
|  | Concerns about the clinic/ hospital or the people who work there |  |
|  | Friends, family members or community members are against vaccination |  |
|  | Because the benefits of vaccination are unclear |  |
|  | Because other things get in the way or are higher priority than vaccines |  |
|  | Because there are better alternatives to vaccination |  |
|  | Because their religion forbids vaccination |  |
|  | Concerns about how long it takes/how long they have to wait |  |
|  | Concerns about catching an illness while at the clinic/ hospital |  |
|  | Because there are no vaccines available at the facility |  |
|  | Because the vaccines are expired |  |
|  | Don’t know/can’t think of any reasons |  |
|  | Other (specify) |  |

ASK ALL

E6.

How much do you agree or disagree with the following statements about vaccines?

Please tell me how much you agree or disagree with each statement by saying a number from 1 to 5, where 1 means ‘Strongly Disagree’, 2 means slightly disagree, 3 means neither agree nor disagree, 4 means slightly agree and 5 means strongly agree

SINGLE ANSWER PER ROW

| **Strongly Disagree** | **Slightly disagree** | **Neither Agree**  **Nor Disagree** | **Slightly agree** | **Strongly agree** |
| --- | --- | --- | --- | --- |
| 1 | 2 | 3 | 4 | 5 |

ADD ‘DON’T KNOW/ NOT APPLICABLE’ OPTION FOR EACH ROW

| **Code Number** | **Code Label** | **Scripting / Routing Instruction** |
| --- | --- | --- |
|  | Vaccinations are too painful for children |  |
|  | FOR PARTIAL AND FULL-VACCINATORS (S11): It is difficult for me to watch my child being vaccinated  FOR NON-VACCINATORS (S11): It would be difficult for me to watch my child being vaccinated |  |
|  | Having many vaccinations at once is hard for children to bear |  |
|  | Vaccinations can permanently disable a child |  |
|  | The side effects (fever, rash, pain) of vaccination are harmful |  |
|  | It is difficult for me to manage the side effects (fever, rash, pain) of vaccination |  |
|  | I worry that the vaccines are expired |  |
|  | Vaccines are a way for global/western countries/organisations to control us |  |
|  |  |  |
|  | I don’t know about the vaccination calendar, so cannot organize my visits to the clinic |  |

RANDOMISE STATEMENTS

ASK ALL

E7.

How much do you agree or disagree with the following statements **about the vaccination process**?

Please tell me how much you agree or disagree with each statement by saying a number from 1 to 5, where 1 means ‘Strongly Disagree’, 2 means slightly disagree, 3 means neither agree nor disagree, 4 means slightly agree and 5 means strongly agree

SINGLE ANSWER PER ROW

| **Strongly Disagree** | **Slightly disagree** | **Neither Agree**  **Nor Disagree** | **Slightly agree** | **Strongly agree** |
| --- | --- | --- | --- | --- |
| 1 | 2 | 3 | 4 | 5 |

ADD ‘DON’T KNOW/ NOT APPLICABLE’ OPTION FOR EACH ROW

| **Code Number** | **Code Label** | **Scripting / Routing Instruction** |
| --- | --- | --- |
|  | The clinic or hospital or outreach site where the vaccination takes place is far away from where I live |  |
|  | It is expensive to travel to where the vaccination takes place |  |
|  | I worry about losing income if I go to the clinic or hospital for vaccination |  |
|  | The clinics or hospitals always have vaccines available |  |
|  | It is not safe to travel to the clinic or hospital where the vaccination takes place |  |
|  | I travel a lot so it’s hard to take my child to get vaccinated |  |
|  | I’m concerned about going to the clinic and getting infected with Covid 19 |  |
|  | Vaccinations are not given if you miss the scheduled date |  |
|  | Vaccinations are available any day of the week |  |
|  | I am too busy to go to the clinic or hospital for vaccinations |  |
|  | I have to pay for vaccinations at the clinic |  |
|  | Vaccination is convenient/ easy to do |  |

RANDOMISE STATEMENTS

ASK ALL

E8. Thinking about the clinic or hospital, or the people who work there. How much do you agree or disagree which the following statements?

Please tell me how much you agree or disagree with each statement by saying a number from 1 to 5, where 1 means ‘Strongly Disagree’, 2 means slightly disagree, 3 means neither agree nor disagree, 4 means slightly agree and 5 means strongly agree

SINGLE ANSWER PER ROW

| **Strongly Disagree** | **Slightly disagree** | **Neither Agree**  **Nor Disagree** | **Slightly agree** | **Strongly agree** |
| --- | --- | --- | --- | --- |
| 1 | 2 | 3 | 4 | 5 |

ADD ‘DON’T KNOW/ NOT APPLICABLE’ OPTION FOR EACH ROW

| **Code Number** | **Code Label** | **Scripting / Routing Instruction** |
| --- | --- | --- |
|  | I trust the nurses who give the vaccination |  |
|  | The staff in the hospital are rude to me |  |
|  | The clinic or hospital is dirty |  |
|  | The queues are too long at the clinic/ hospital where the vaccination takes place |  |
|  | The healthcare facility is too expensive |  |
|  | I am concerned about catching an illness while at the clinic/ hospital |  |

RANDOMISE STATEMENTS

ASK ALL

E9. Thinking about the need for vaccinations…

To what extent do you agree or disagree with the following statements? Please tell me how much you agree or disagree with each statement by saying a number from 1 to 5, where 1 means ‘Strongly Disagree’, 2 means slightly disagree, 3 means neither agree nor disagree, 4 means slightly agree and 5 means strongly agree

SINGLE ANSWER PER ROW

| **Strongly Disagree** | **Slightly disagree** | **Neither Agree**  **Nor Disagree** | **Slightly agree** | **Strongly agree** |
| --- | --- | --- | --- | --- |
| 1 | 2 | 3 | 4 | 5 |

ADD ‘DON’T KNOW/ NOT APPLICABLE’ OPTION FOR EACH ROW

| **Code Number** | **Code Label** | **Scripting / Routing Instruction** |
| --- | --- | --- |
|  | There are no benefits to vaccination |  |
|  | I believe that vaccines are effective |  |
|  | Children who have not had vaccinations are usually healthy |  |
|  | There are other ways I can protect my child from disease |  |

RANDOMISE STATEMENTS

ASK ALL

E18. I will now read you a series of statements about vaccinations. Please tell me if you think they are true or false:

SINGLE ANSWER PER ROW

| **False** | **True** |
| --- | --- |

ADD ‘DON’T KNOW/ NOT APPLICABLE’ OPTION FOR EACH ROW

| **Code Number** | **Code Label** | **Scripting / Routing Instruction** |
| --- | --- | --- |
|  | Vaccines can be given to children when they are ill to make them feel better |  |
|  | Vaccination stops children from getting some illnesses |  |
|  | If a vaccinated child gets sick, it will be less serious than if a non-vaccinated child gets sick |  |
|  | There are vaccines for all childhood diseases |  |
|  | Vaccinations can make children infertile |  |

RANDOMISE CODES

ASK ALL

E19. As far as you know, is there a vaccine available in this country for the following diseases?

SINGLE ANSWER PER ROW

| **Code Number** | **Code Label** | **Scripting / Routing Instruction** |
| --- | --- | --- |
|  | Yes |  |
|  | No |  |

ADD ‘DON’T KNOW/ NOT APPLICABLE’ OPTION FOR EACH ROW

| **Code Number** | **Code Label** | **Scripting / Routing Instruction** |
| --- | --- | --- |
|  | Measles |  |
|  | Malaria |  |
|  | Polio |  |
|  | Headache |  |

RANDOMISE CODES

ASK ALL

E20. Has your child ever been vaccinated during a campaign? This is when, after a mass communication, someone comes to your community specifically to vaccinate a lot of children over a few days.

SINGLE ANSWER

| **Code Number** | **Code Label** | **Scripting / Routing Instruction** |
| --- | --- | --- |
|  | Yes |  |
|  | No |  |
|  | Don’t remember/ not sure |  |

ASK IF E20=1

E21. What other services were offered to you and your children during the vaccination campaign?

MULTICODE. DON’T READ OUT THE LIST

| **Code Number** | **Code Label** | **Scripting / Routing Instruction** |
| --- | --- | --- |
|  | Mosquito net |  |
|  | Vitamin A supplementation |  |
|  | Deworming |  |
|  | Food supplies |  |
|  | Other |  |
|  | Nothing |  |
|  | Don’t remember/ not sure |  |

ASK ALL

E22. How much do you agree or disagree with the following statements about campaign vaccination?

Please tell me how much you agree or disagree with each statement by saying a number from 1 to 5, where 1 means ‘Strongly Disagree’, 2 means slightly disagree, 3 means neither agree nor disagree, 4 means slightly agree and 5 means strongly agree

SINGLE ANSWER PER ROW

| **Strongly Disagree** | **Slightly disagree** | **Neither Agree**  **Nor Disagree** | **Slightly agree** | **Strongly agree** |
| --- | --- | --- | --- | --- |
| 1 | 2 | 3 | 4 | 5 |

ADD ‘DON’T KNOW/ NOT APPLICABLE’ OPTION FOR EACH ROW

| **Code Number** | **Code Label** | **Scripting / Routing Instruction** |
| --- | --- | --- |
|  | I trust campaign workers to vaccinate my children |  |
|  | There are too many vaccine campaigns in this area |  |
|  | I prefer campaigns for vaccination, compared to visiting a clinic |  |
|  | Vaccines that are not offered during campaigns are less important |  |
|  | Outreach sessions (when people come to the area to give health services) are unreliable |  |
|  | All recommended vaccinations are available in a campaign |  |

RANDOMISE STATEMENTS

ASK ALL

E10.

Looking at the following list of things done for children, please choose the 3 most important and put them in order of importance from highest to lowest; where 1 is the most important, 2 is the second most important and 3 is the third most important.

READ OUT ALL OPTIONS TO RESPONDENT BEFORE ASKING THEM TO RANK THE 3 MOST IMPORTANT. WRITE IN NUMBER 1 -3 BASED ON RESPONDENT’S ANSWER. USE SHOW CARD IF NEEDED/APPROPRIATE

| **Code Number** | **Code Label** | **Scripting / Routing Instruction** |
| --- | --- | --- |
|  |  |  |
|  | Paying school fees on time |  |
|  | Taking children for all vaccinations |  |
|  | Making sure a child always sleeps under a mosquito net |  |
|  | Being watchful of who a child plays with |  |
|  | Regularly giving them deworming medication |  |

ONLY NUMBER 1,2,3 TO BE ACCEPTED. MUST BE DIFFERENT NUMBER PER OPTION

ASK ALL WHO SELECT 4 OR 5 AT CODE 4 (There are other ways I can protect my child from disease) AT E9

E11. Which of the following, if any, do you believe are better alternatives to vaccination?

MULTICODE

| **Code Number** | **Code Label** | **Scripting / Routing Instruction** |
| --- | --- | --- |
|  | God’s protection/ prayer |  |
|  | Traditional/ herbal remedies |  |
|  | Maintaining a clean house |  |
|  | Bathing the child regularly |  |
|  | Breastfeeding |  |
|  | Keeping the child warm |  |
|  | None of the above- there are no better alternatives |  |
|  | Other (specify) |  |

ASK ALL

E12. What are the reasons why people in your community/ people you know of **DO** want to take their children for vaccination?

MULTICODE, DO NOT READ LIST

| **Code Number** | **Code Label** | **Scripting / Routing Instruction** |
| --- | --- | --- |
|  | Vaccines prevent disease |  |
|  | Vaccines prevent children from becoming disabled |  |
|  | Vaccines reduce the severity of disease |  |
|  | Vaccination is convenient/ easy to do |  |
|  | Family supports vaccination |  |
|  | Everyone in the community vaccinates their children |  |
|  | Being vaccinated may open up opportunities later in life (e.g. education, foreign travel) |  |
|  | If they see how children can be protected by vaccination |  |
|  | Religious leaders are supportive of vaccination |  |
|  | Trust that the government knows what is right for children |  |
|  | The service provided by clinics and hospitals is good |  |
|  | Afraid of punishment if children are not vaccinated |  |
|  | So their children can go to school |  |
|  | It’s part of being a good parent |  |
|  | None/ no reasons |  |
|  | Don’t know |  |
|  | Other (specify) |  |

ASK ALL

E13. What, if anything, would make vaccination easier for you?

MULTICODE, DO NOT READ OUT LIST

| **Code Number** | **Code Label** | **Scripting / Routing Instruction** |
| --- | --- | --- |
|  | Providing vaccinations at a community meeting place |  |
|  | Providing vaccinations in your home |  |
|  | Providing vaccinations in your child’s school/nursery |  |
|  | Providing vaccinations with other health services (e.g. with family planning services, ante natal care) |  |
|  | Health workers more friendly/informed |  |
|  | Less disruption from side effects after vaccination |  |
|  | Fewer injections during a single visit |  |
|  | Making sure vaccines are always available at the healthcare facility |  |
|  | Providing more information about vaccines |  |
|  | Having more healthcare workers at the clinic |  |
|  | A clean clinic/ hospital |  |
|  | Reducing the waiting time, no queues |  |
|  | Don’t know |  |
|  | Other (specify) |  |

ASK ALL

E14. How much do you agree with the following statements about vaccination?

Please tell me how much you agree or disagree with each statement by saying a number from 1 to 5, where 1 means ‘Strongly Disagree’, 2 means slightly disagree, 3 means neither agree nor disagree, 4 means slightly agree and 5 means strongly agree

SINGLE ANSWER PER ROW

| **Strongly Disagree** | **Slightly disagree** | **Neither Agree**  **Nor Disagree** | **Slightly agree** | **Strongly agree** |
| --- | --- | --- | --- | --- |
| 1 | 2 | 3 | 4 | 5 |

DOWN SIDE OF GRID // ADD ‘DON’T KNOW/ NOT APPLICABLE’ OPTION FOR EACH ROW

| **Code Number** | **Code Label** | **Scripting / Routing Instruction** |
| --- | --- | --- |
|  |  |  |
|  |  |  |
|  | My spouse / partner helped/ ensured that my child was vaccinated |  |
|  | My mother/ mother-in-law  helped/ ensured that my child was vaccinated |  |
|  |  |  |
|  | It is normal in this community to vaccinate your children |  |
|  | Religious leaders are supportive of vaccination |  |
|  | I trust that the government knows what is right for children |  |
|  | I am afraid of punishment if my child is not vaccinated |  |
|  | It is part of my role to ensure that my child is vaccinated |  |

RANDOMISE

ASK PARTIALLY VACCINATED (FROM S11)

AND NOT VACCINATED (FROM S11) –

E15.

IF PARTIALLY VACCINATED: What, if anything, would have allowed you to ensure that your child finished the vaccination course?

IF NOT VACCINATED: What, if anything, would have encouraged you to take your child for vaccination?

MULTICODE- DO NOT READ OUT LIST

| **Code Number** | **Code Label** | **Scripting / Routing Instruction** |
| --- | --- | --- |
|  | More information about vaccines and what they do | PERSUASION THAT VACCINES ARE WORTH IT |
|  | More reassurance on the safety of vaccines | PERSUASION THAT VACCINES ARE WORTH IT |
|  | More advice on how to deal with side effects after vaccination | PERSUASION THAT VACCINES ARE WORTH IT |
|  | Help with getting to the clinic or outreach session | ACCESS AND OPPORTUNITY |
|  | Friendly and skilled staff (e.g. nurses) | ACCESS AND OPPORTUNITY |
|  | No long queues/waiting | ACCESS AND OPPORTUNITY |
|  | More support from my husband | SUPPORT FROM NETWORK |
|  | More support from other members of my family | SUPPORT FROM NETWORK |
|  | Help with basic necessities to ensure I can make time for vaccinations | SUPPORT FROM NETWORK |
|  | Better reminders about when to return for next vaccinations |  |
|  | More convenient services (when and where provided) |  |
|  | Others |  |
|  | Nothing |  |

ASK ALL

E16.

Who or where would you go to if you wanted information about vaccines?

MULTICODE, DO NOT READ OUT LIST

| **Code Number** | **Code Label** | **Scripting / Routing Instruction** |
| --- | --- | --- |
|  | Your spouse/ partner |  |
|  | Your mother |  |
|  | Your mother-in-law |  |
|  | Elders in the community |  |
|  | Religious leaders |  |
|  | Traditional leaders |  |
|  | Traditional medical practitioners |  |
|  | Doctors or nurses (Health workers) |  |
|  | Representatives of the government |  |
|  | NGO representatives |  |
|  | Teachers |  |
|  | Pharmacists / PMVs/Chemists |  |
|  | Friends |  |
|  | Singers/ musicians/ athletes |  |
|  | Social media (e.g. Instagram, Facebook) |  |
|  | Websites online |  |
|  | Radio/ TV/ newspapers |  |
|  | Street doctors |  |
|  | Other (specify) |  |
|  | No-one |  |

That is the end of our questions, thank you very much for your time and for telling us your opinions

FOR MODERATOR

E17.

Do you feel that this respondent was a non-vaccinator?

SINGLE CODE

| **Code Number** | **Code Label** | **Scripting / Routing Instruction** |
| --- | --- | --- |
|  | Yes (please specify- write comments) |  |
|  | No |  |

**[END OF SURVEY]**
