# Supplementary material for: A structural equation modelling approach to understanding the determinants of childhood vaccination in Nigeria, Uganda and Guinea
Source: PLOS Glob Public Health. 2023 Mar 29;3(3):e0001289. doi: 10.1371/journal.pgph.0001289 (PMC10058155; doi:10.1371/journal.pgph.0001289)
Supplement: S3 Text — (DOCX) [file pgph.0001289.s004.docx]

Vaccines SEM: Country Analysis

Table of Contents

[Introduction 2](#_heading=h.30j0zll)

[Nigeria 3](#_heading=h.3znysh7)

[Factor analysis and Measurement Model 3](#_heading=h.tyjcwt)

[Structural Model 6](#_heading=h.1t3h5sf)

[Interpretation 9](#_heading=h.2s8eyo1)

[Uganda 10](#_heading=h.3rdcrjn)

[Factor analysis and Measurement Model 10](#_heading=h.lnxbz9)

[Structural Model 13](#_heading=h.1ksv4uv)

[Interpretation 16](#_heading=h.2jxsxqh)

[Guinea 17](#_heading=h.3j2qqm3)

[Factor analysis and Measurement Model 17](#_heading=h.4i7ojhp)

[Structural Model 20](#_heading=h.1ci93xb)

[Interpretation 23](#_heading=h.2bn6wsx)

# Introduction

This supplementary document shows the results of the factor analysis and structural equation modelling for Nigeria, Uganda and Guinea separately.

For each country, the factor analysis was repeated, which may have resulted in different factors from the main paper. Then, the measurement and structural models were run.

# Nigeria

## Factor analysis and Measurement Model

The factor analysis for Nigeria produced 4 factors:

| Factor | Variables |
| --- | --- |
| Support for vaccination from others (A) | My spouse / partner helped/ ensured that my child was vaccinated (E14_3_E14), My mother/ mother-in-law helped/ ensured that my child was vaccinated (E14_4_E14), It is normal in this community to vaccinate your children (E14_6_E14), Religious leaders are supportive of vaccination (E14_7_E14), I trust that the government knows what is right for children (E14_8_E14) |
| Bad service delivery experience (B) | The clinic or hospital or outreach site where the vaccination takes place is far away from where I live (E7_1_E7), It is not safe to travel to the clinic or hospital where the vaccination takes place (E7_5_E7), I am too busy to go to the clinic or hospital for vaccinations (E7_10_E7), The clinic or hospital is dirty (E8_3_E8) |
| Belief that vaccines are harmful (C) | Having many vaccinations at once is hard for children to bear, (E6_3_E6), It is difficult for me to manage the side effects (fever, rash, pain) of vaccination (E6_7_E6), Vaccines are a way for global/western countries/organisations to control us (E6_9_E6) |
| Belief in religious protection (D) | My religious faith protects me and my family from harm (C5_1_C5), My religious faith heals me and my family from illnesses (C5_2_C5), God is the only protection needed against harm (C5_3_C5), My religious faith guides decisions in my life (C5_4_C5) |

The measurement model for these factors is given below.

## lavaan 0.6-10 ended normally after 56 iterations
##
## Estimator ML
## Optimization method NLMINB
## Number of model parameters 38
##
## Number of observations 1264
##
## Model Test User Model:
##
## Test statistic 338.429
## Degrees of freedom 98
## P-value (Chi-square) 0.000
##
## Model Test Baseline Model:
##
## Test statistic 4280.657
## Degrees of freedom 120
## P-value 0.000
##
## User Model versus Baseline Model:
##
## Comparative Fit Index (CFI) 0.942
## Tucker-Lewis Index (TLI) 0.929
##
## Loglikelihood and Information Criteria:
##
## Loglikelihood user model (H0) -27289.501
## Loglikelihood unrestricted model (H1) -27120.286
##
## Akaike (AIC) 54655.002
## Bayesian (BIC) 54850.399
## Sample-size adjusted Bayesian (BIC) 54729.694
##
## Root Mean Square Error of Approximation:
##
## RMSEA 0.044
## 90 Percent confidence interval - lower 0.039
## 90 Percent confidence interval - upper 0.049
## P-value RMSEA <= 0.05 0.971
##
## Standardized Root Mean Square Residual:
##
## SRMR 0.043
##
## Parameter Estimates:
##
## Standard errors Standard
## Information Expected
## Information saturated (h1) model Structured
##
## Latent Variables:
## Estimate Std.Err z-value P(>|z|) Std.lv Std.all
## A =~
## E14_3_E14 1.000 0.778 0.743
## E14_4_E14 1.023 0.048 21.104 0.000 0.796 0.711
## E14_6_E14 0.690 0.035 19.485 0.000 0.537 0.640
## E14_7_E14 0.642 0.036 18.056 0.000 0.500 0.587
## E14_8_E14 0.688 0.043 16.000 0.000 0.536 0.515
## B =~
## E7_1_E7 1.000 0.714 0.483
## E7_5_E7 1.274 0.100 12.745 0.000 0.910 0.639
## E7_10_E7 1.196 0.096 12.467 0.000 0.854 0.600
## E8_3_E8 1.010 0.083 12.214 0.000 0.721 0.572
## C =~
## E6_3_E6 1.000 0.400 0.318
## E6_7_E6 2.206 0.279 7.917 0.000 0.881 0.619
## E6_9_E6 2.134 0.271 7.872 0.000 0.853 0.561
## D =~
## C5_1_C5 1.000 0.437 0.721
## C5_2_C5 1.034 0.059 17.663 0.000 0.452 0.609
## C5_3_C5 0.866 0.048 17.952 0.000 0.379 0.622
## C5_4_C5 1.042 0.053 19.559 0.000 0.455 0.715
##
## Covariances:
## Estimate Std.Err z-value P(>|z|) Std.lv Std.all
## A ~~
## B -0.169 0.024 -6.892 0.000 -0.304 -0.304
## C -0.069 0.015 -4.463 0.000 -0.222 -0.222
## D 0.097 0.013 7.377 0.000 0.284 0.284
## B ~~
## C 0.185 0.027 6.923 0.000 0.647 0.647
## D -0.077 0.013 -5.745 0.000 -0.246 -0.246
## C ~~
## D -0.002 0.008 -0.253 0.801 -0.011 -0.011
##
## Variances:
## Estimate Std.Err z-value P(>|z|) Std.lv Std.all
## .E14_3_E14 0.492 0.029 17.005 0.000 0.492 0.448
## .E14_4_E14 0.621 0.034 18.371 0.000 0.621 0.495
## .E14_6_E14 0.416 0.020 20.591 0.000 0.416 0.590
## .E14_7_E14 0.475 0.022 21.725 0.000 0.475 0.656
## .E14_8_E14 0.795 0.035 22.811 0.000 0.795 0.735
## .E7_1_E7 1.675 0.076 21.983 0.000 1.675 0.767
## .E7_5_E7 1.201 0.067 17.875 0.000 1.201 0.592
## .E7_10_E7 1.294 0.067 19.203 0.000 1.294 0.640
## .E8_3_E8 1.069 0.053 20.031 0.000 1.069 0.673
## .E6_3_E6 1.417 0.061 23.358 0.000 1.417 0.899
## .E6_7_E6 1.250 0.085 14.716 0.000 1.250 0.617
## .E6_9_E6 1.585 0.091 17.382 0.000 1.585 0.685
## .C5_1_C5 0.177 0.011 16.812 0.000 0.177 0.481
## .C5_2_C5 0.347 0.017 20.687 0.000 0.347 0.629
## .C5_3_C5 0.227 0.011 20.364 0.000 0.227 0.613
## .C5_4_C5 0.198 0.012 17.085 0.000 0.198 0.489
## A 0.606 0.044 13.618 0.000 1.000 1.000
## B 0.510 0.067 7.615 0.000 1.000 1.000
## C 0.160 0.035 4.500 0.000 1.000 1.000
## D 0.191 0.015 12.716 0.000 1.000 1.000

Summary:

| **Factor** | **Variable** | **Factor loading** | **Standard error** | **P-value** | **Standardised factor loading** |
| --- | --- | --- | --- | --- | --- |
| Support for vaccination from others (A) | My spouse / partner helped/ ensured that my child was vaccinated | 1.00 |  |  | 0.74 |
|  | My mother/ mother-in-law helped/ ensured that my child was vaccinated | 1.02 | 0.05 | <0.001 | 0.71 |
|  | It is normal in this community to vaccinate your children | 0.69 | 0.04 | <0.001 | 0.64 |
|  | Religious leaders are supportive of vaccination | 0.64 | 0.04 | <0.001 | 0.59 |
|  | I trust that the government knows what is right for children | 0.69 | 0.04 | <0.001 | 0.52 |
| Bad service delivery experience (B) | The clinic or hospital or outreach site where the vaccination takes place is far away from where I live | 1.00 |  |  | 0.48 |
|  | It is not safe to travel to the clinic or hospital where the vaccination takes place | 1.27 | 0.10 | <0.001 | 0.64 |
|  | I am too busy to go to the clinic or hospital for vaccinations | 1.20 | 0.10 | <0.001 | 0.60 |
|  | The clinic or hospital is dirty | 1.01 | 0.08 | <0.001 | 0.57 |
| Belief that vaccines are harmful (C) | Having many vaccinations at once is hard for children to bear | 1.00 |  |  | 0.32 |
|  | It is difficult for me to manage the side effects (fever, rash, pain) of vaccination | 2.21 | 0.28 | <0.001 | 0.62 |
|  | Vaccines are a way for global/western countries/organisations to control us | 2.13 | 0.27 | <0.001 | 0.56 |
| Belief in religious protection (D) | My religious faith protects me and my family from harm | 1.00 |  |  | 0.72 |
|  | My religious faith heals me and my family from illnesses | 1.03 | 0.06 | <0.001 | 0.61 |
|  | God is the only protection needed against harm | 0.87 | 0.05 | <0.001 | 0.52 |
|  | My religious faith guides decisions in my life | 1.04 | 0.05 | <0.001 | 0.72 |

The measurement model showed an acceptable fit to the data: CFI = 0.94, TLI = 0.93, RMSEA = 0.04, SRMR = 0.04

## Structural Model

The structural model for Nigeria is given below.

## lavaan 0.6-10 ended normally after 99 iterations
##
## Estimator DWLS
## Optimization method NLMINB
## Number of model parameters 59
##
## Number of observations 1264
##
## Model Test User Model:
## Standard Robust
## Test Statistic 273.342 378.818
## Degrees of freedom 110 110
## P-value (Chi-square) 0.000 0.000
## Scaling correction factor 0.779
## Shift parameter 28.143
## simple second-order correction
##
## Model Test Baseline Model:
##
## Test statistic 7223.680 3655.642
## Degrees of freedom 136 136
## P-value 0.000 0.000
## Scaling correction factor 2.014
##
## User Model versus Baseline Model:
##
## Comparative Fit Index (CFI) 0.977 0.924
## Tucker-Lewis Index (TLI) 0.972 0.906
##
## Robust Comparative Fit Index (CFI) NA
## Robust Tucker-Lewis Index (TLI) NA
##
## Root Mean Square Error of Approximation:
##
## RMSEA 0.034 0.044
## 90 Percent confidence interval - lower 0.029 0.039
## 90 Percent confidence interval - upper 0.039 0.049
## P-value RMSEA <= 0.05 1.000 0.979
##
## Robust RMSEA NA
## 90 Percent confidence interval - lower NA
## 90 Percent confidence interval - upper NA
##
## Standardized Root Mean Square Residual:
##
## SRMR 0.041 0.041
##
## Parameter Estimates:
##
## Standard errors Robust.sem
## Information Expected
## Information saturated (h1) model Unstructured
##
## Latent Variables:
## Estimate Std.Err z-value P(>|z|) Std.lv Std.all
## A =~
## E14_3_E14 1.000 0.715 0.682
## E14_4_E14 1.024 0.065 15.882 0.000 0.732 0.654
## E14_6_E14 0.830 0.052 16.072 0.000 0.593 0.706
## E14_7_E14 0.732 0.046 16.067 0.000 0.523 0.614
## E14_8_E14 0.731 0.053 13.658 0.000 0.522 0.502
## B =~
## E7_1_E7 1.000 0.579 0.392
## E7_5_E7 1.354 0.166 8.133 0.000 0.784 0.551
## E7_10_E7 1.703 0.216 7.891 0.000 0.987 0.694
## E8_3_E8 1.325 0.169 7.843 0.000 0.768 0.609
## C =~
## E6_3_E6 1.000 0.279 0.222
## E6_7_E6 3.513 0.674 5.215 0.000 0.979 0.687
## E6_9_E6 3.158 0.610 5.178 0.000 0.880 0.579
## D =~
## C5_1_C5 1.000 0.444 0.732
## C5_2_C5 1.022 0.045 22.708 0.000 0.453 0.611
## C5_3_C5 0.840 0.036 23.170 0.000 0.373 0.612
## C5_4_C5 1.020 0.040 25.623 0.000 0.452 0.710
##
## Regressions:
## Estimate Std.Err z-value P(>|z|) Std.lv Std.all
## outcome ~
## A 0.514 0.062 8.291 0.000 0.368 0.368
## B -0.346 0.126 -2.745 0.006 -0.200 -0.200
## C 0.046 0.246 0.187 0.852 0.013 0.013
## D -0.074 0.095 -0.786 0.432 -0.033 -0.033
##
## Covariances:
## Estimate Std.Err z-value P(>|z|) Std.lv Std.all
## A ~~
## B -0.139 0.022 -6.232 0.000 -0.335 -0.335
## C -0.042 0.012 -3.591 0.000 -0.212 -0.212
## D 0.098 0.011 9.316 0.000 0.310 0.310
## B ~~
## C 0.097 0.022 4.360 0.000 0.603 0.603
## D -0.064 0.012 -5.281 0.000 -0.248 -0.248
## C ~~
## D -0.001 0.006 -0.149 0.882 -0.007 -0.007
##
## Intercepts:
## Estimate Std.Err z-value P(>|z|) Std.lv Std.all
## .E14_3_E14 4.294 0.050 85.172 0.000 4.294 4.097
## .E14_4_E14 4.161 0.051 81.977 0.000 4.161 3.716
## .E14_6_E14 4.450 0.038 117.858 0.000 4.450 5.301
## .E14_7_E14 4.449 0.038 116.947 0.000 4.449 5.224
## .E14_8_E14 4.194 0.045 93.030 0.000 4.194 4.032
## .E7_1_E7 2.695 0.044 61.132 0.000 2.695 1.823
## .E7_5_E7 2.408 0.051 47.333 0.000 2.408 1.691
## .E7_10_E7 2.281 0.056 40.742 0.000 2.281 1.604
## .E8_3_E8 2.045 0.055 37.118 0.000 2.045 1.622
## .E6_3_E6 3.775 0.047 79.696 0.000 3.775 3.006
## .E6_7_E6 3.032 0.040 75.205 0.000 3.032 2.129
## .E6_9_E6 2.710 0.045 60.215 0.000 2.710 1.782
## .C5_1_C5 4.685 0.027 173.176 0.000 4.685 7.727
## .C5_2_C5 4.596 0.036 127.163 0.000 4.596 6.191
## .C5_3_C5 4.728 0.031 152.403 0.000 4.728 7.767
## .C5_4_C5 4.672 0.029 161.060 0.000 4.672 7.335
## .outcome 0.000 0.000 0.000
## A 0.000 0.000 0.000
## B 0.000 0.000 0.000
## C 0.000 0.000 0.000
## D 0.000 0.000 0.000
##
## Thresholds:
## Estimate Std.Err z-value P(>|z|) Std.lv Std.all
## outcome|t1 0.356 0.036 9.875 0.000 0.356 0.356
##
## Variances:
## Estimate Std.Err z-value P(>|z|) Std.lv Std.all
## .E14_3_E14 0.587 0.026 22.844 0.000 0.587 0.535
## .E14_4_E14 0.718 0.035 20.450 0.000 0.718 0.572
## .E14_6_E14 0.353 0.015 23.322 0.000 0.353 0.501
## .E14_7_E14 0.451 0.017 27.292 0.000 0.451 0.622
## .E14_8_E14 0.809 0.033 24.439 0.000 0.809 0.748
## .E7_1_E7 1.849 0.129 14.379 0.000 1.849 0.846
## .E7_5_E7 1.413 0.088 16.059 0.000 1.413 0.697
## .E7_10_E7 1.050 0.079 13.348 0.000 1.050 0.519
## .E8_3_E8 1.000 0.060 16.631 0.000 1.000 0.629
## .E6_3_E6 1.499 0.088 17.072 0.000 1.499 0.951
## .E6_7_E6 1.070 0.106 10.080 0.000 1.070 0.528
## .E6_9_E6 1.538 0.118 13.074 0.000 1.538 0.665
## .C5_1_C5 0.171 0.007 23.873 0.000 0.171 0.464
## .C5_2_C5 0.345 0.011 30.509 0.000 0.345 0.627
## .C5_3_C5 0.232 0.007 32.689 0.000 0.232 0.625
## .C5_4_C5 0.201 0.007 26.827 0.000 0.201 0.495
## .outcome 0.790 0.790 0.790
## A 0.511 0.050 10.193 0.000 1.000 1.000
## B 0.336 0.075 4.489 0.000 1.000 1.000
## C 0.078 0.027 2.839 0.005 1.000 1.000
## D 0.197 0.012 16.502 0.000 1.000 1.000
##
## Scales y*:
## Estimate Std.Err z-value P(>|z|) Std.lv Std.all
## outcome 1.000 1.000 1.000

## Summary:

| **Factor** | **B (95% CI)** | **β (95% CI)** | **P-value** |
| --- | --- | --- | --- |
| Support for vaccination from others (A) | 0.51 (0.39, 0.64) | 0.37 (0.28. 0.45) | <0.001 |
| Bad service delivery experience (B) | -0.35 (-0.59, -0.10) | -0.20 (-0.33, -0.07) | 0.006 |
| Belief that vaccines are harmful (C) | 0.05 (0.44, 0.53) | 0.01 (-0.12. 0.15) | 0.85 |
| Belief in religious protection (D) | -0.07 (-0.26. 0.11) | -0.03 (-0.12, 0.05) | 0.43 |

## Interpretation

The structural model is shown to be a good fit to the data (CFI = 0.98, TLI = 0.97, RMSEA = 0.03, SRMR = 0.04).

In the regression analysis, Support for vaccination from others (Factor A) was observed to lead to an increase in probability of vaccination (B- unstandardised coefficient = 0.51, $\beta$- standardised coefficient = 0.37, p<0.001). Bad service delivery experience (Factor B) was observed to lead to a decrease in probability of vaccination (B = -0.35, $\beta$ = -0.20, p=0.006). Neither Belief that vaccines are harmful (Factor C) nor Belief in religious protection (Factor D) appear to have a relationship with the vaccination outcome. (C: B = -0.046, $\beta$ = -0.013, p=0.85; D: B = -0.07, $\beta$ = -0.03, p=0.43)

# Uganda

## Factor analysis and Measurement Model

The factor analysis for Uganda produced 4 factors:

| Factor | Variables |
| --- | --- |
| Belief in religious protection (A) | My religious faith protects me and my family from harm (C5_1_C5), My religious faith heals me and my family from illnesses (C5_2_C5), God is the only protection needed against harm (C5_3_C5), My religious faith guides decisions in my life (C5_4_C5) |
| Vaccination is a norm and worth doing (B) | I believe that vaccines are effective (E9_3_E9), It is normal in this community to vaccinate your children (E14_6_E14), Religious leaders are supportive of vaccination (E14_7_E14), I trust that the government knows what is right for children (E14_8_E14) |
| Vaccination is practically difficult (C) | I travel a lot so it’s hard to take my child to get vaccinated (E7_6_E7), It is not safe to travel to the clinic or hospital where the vaccination takes place (E7_5_E7), Vaccinations are not given if you miss the scheduled date (E7_8_E7), I am too busy to go to the clinic or hospital for vaccinations (E7_10_E7) |
| Poor service delivery experience (D) | The staff in the hospital are rude to me (E8_2_E8), The clinic or hospital is dirty (E8_3_E8), The queues are too long at the clinic/ hospital where the vaccination takes place (E8_4_E8) |

The measurement model for these factors is given below.

## lavaan 0.6-10 ended normally after 49 iterations
##
## Estimator ML
## Optimization method NLMINB
## Number of model parameters 36
##
## Number of observations 1054
##
## Model Test User Model:
##
## Test statistic 167.654
## Degrees of freedom 84
## P-value (Chi-square) 0.000
##
## Model Test Baseline Model:
##
## Test statistic 1951.553
## Degrees of freedom 105
## P-value 0.000
##
## User Model versus Baseline Model:
##
## Comparative Fit Index (CFI) 0.955
## Tucker-Lewis Index (TLI) 0.943
##
## Loglikelihood and Information Criteria:
##
## Loglikelihood user model (H0) -24206.325
## Loglikelihood unrestricted model (H1) -24122.497
##
## Akaike (AIC) 48484.649
## Bayesian (BIC) 48663.222
## Sample-size adjusted Bayesian (BIC) 48548.880
##
## Root Mean Square Error of Approximation:
##
## RMSEA 0.031
## 90 Percent confidence interval - lower 0.024
## 90 Percent confidence interval - upper 0.037
## P-value RMSEA <= 0.05 1.000
##
## Standardized Root Mean Square Residual:
##
## SRMR 0.039
##
## Parameter Estimates:
##
## Standard errors Standard
## Information Expected
## Information saturated (h1) model Structured
##
## Latent Variables:
## Estimate Std.Err z-value P(>|z|) Std.lv Std.all
## A =~
## C5_1_C5 1.000 0.830 0.734
## C5_2_C5 1.107 0.081 13.601 0.000 0.918 0.653
## C5_3_C5 0.539 0.047 11.485 0.000 0.447 0.466
## C5_4_C5 0.561 0.047 11.885 0.000 0.466 0.489
## B =~
## E9_3_E9 1.000 0.514 0.588
## E14_6_E14 1.016 0.092 11.063 0.000 0.522 0.586
## E14_7_E14 0.756 0.089 8.485 0.000 0.388 0.369
## E14_8_E14 0.968 0.097 10.000 0.000 0.498 0.469
## C =~
## E7_6_E7 1.000 0.819 0.618
## E7_5_E7 0.648 0.074 8.783 0.000 0.531 0.368
## E7_8_E7 0.757 0.075 10.113 0.000 0.620 0.444
## E7_10_E7 0.890 0.076 11.772 0.000 0.728 0.579
## D =~
## E8_2_E8 1.000 0.902 0.586
## E8_3_E8 1.005 0.122 8.232 0.000 0.906 0.611
## E8_4_E8 0.339 0.063 5.388 0.000 0.306 0.236
##
## Covariances:
## Estimate Std.Err z-value P(>|z|) Std.lv Std.all
## A ~~
## B 0.091 0.021 4.346 0.000 0.214 0.214
## C -0.018 0.032 -0.575 0.565 -0.027 -0.027
## D 0.003 0.037 0.075 0.940 0.004 0.004
## B ~~
## C -0.256 0.029 -8.960 0.000 -0.608 -0.608
## D -0.147 0.028 -5.200 0.000 -0.316 -0.316
## C ~~
## D 0.408 0.052 7.764 0.000 0.552 0.552
##
## Variances:
## Estimate Std.Err z-value P(>|z|) Std.lv Std.all
## .C5_1_C5 0.590 0.052 11.459 0.000 0.590 0.462
## .C5_2_C5 1.134 0.075 15.157 0.000 1.134 0.574
## .C5_3_C5 0.720 0.035 20.319 0.000 0.720 0.782
## .C5_4_C5 0.692 0.035 19.964 0.000 0.692 0.761
## .E9_3_E9 0.501 0.031 16.093 0.000 0.501 0.655
## .E14_6_E14 0.522 0.032 16.160 0.000 0.522 0.657
## .E14_7_E14 0.955 0.046 20.958 0.000 0.955 0.863
## .E14_8_E14 0.879 0.045 19.348 0.000 0.879 0.780
## .E7_6_E7 1.084 0.070 15.582 0.000 1.084 0.618
## .E7_5_E7 1.799 0.085 21.150 0.000 1.799 0.865
## .E7_8_E7 1.565 0.078 20.102 0.000 1.565 0.803
## .E7_10_E7 1.050 0.062 16.936 0.000 1.050 0.664
## .E8_2_E8 1.551 0.119 13.087 0.000 1.551 0.656
## .E8_3_E8 1.375 0.115 11.978 0.000 1.375 0.626
## .E8_4_E8 1.587 0.072 21.990 0.000 1.587 0.944
## A 0.689 0.067 10.341 0.000 1.000 1.000
## B 0.264 0.034 7.868 0.000 1.000 1.000
## C 0.670 0.079 8.503 0.000 1.000 1.000
## D 0.813 0.125 6.527 0.000 1.000 1.000

Summary

| **Factor** | **Variable** | **Factor loading** | **Standard error** | **P-value** | **Standardised factor loading** |
| --- | --- | --- | --- | --- | --- |
| Belief in religious protection (A) | My religious faith protects me and my family from harm | 1.00 |  |  | 0.73 |
|  | My religious faith heals me and my family from illnesses | 1.12 | 0.08 | <0.001 | 0.65 |
|  | God is the only protection needed against harm | 0.54 | 0.05 | <0.001 | 0.47 |
|  | My religious faith guides decisions in my life | 0.56 | 0.05 | <0.001 | 0.49 |
| Vaccination is a norm and worth doing (B) | I believe that vaccines are effective | 1.00 |  |  | 0.59 |
|  | It is normal in this community to vaccinate your children | 1.02 | 0.09 | <0.001 | 0.59 |
|  | Religious leaders are supportive of vaccination | 0.76 | 0.09 | <0.001 | 0.40 |
|  | I trust that the government knows what is right for children | 0.97 | 0.10 | <0.001 | 0.47 |
| Vaccination is practically difficult (C) | I travel a lot so it’s hard to take my child to get vaccinated | 1.00 |  |  | 0.62 |
|  | It is not safe to travel to the clinic or hospital where the vaccination takes place | 0.65 | 0.07 | <0.001 | 0.37 |
|  | Vaccinations are not given if you miss the scheduled date | 0.76 | 0.08 | <0.001 | 0.44 |
|  | I am too busy to go to the clinic or hospital for vaccinations | 0.89 | 0.08 | <0.001 | 0.58 |
| Poor service delivery experience (D) | The staff in the hospital are rude to me | 1.00 |  |  | 0.59 |
|  | The clinic or hospital is dirty | 1.01 | 0.12 | <0.001 | 0.61 |
|  | The queues are too long at the clinic/ hospital where the vaccination takes place | 0.34 | 0.06 | <0.001 | 0.24 |

The measurement model showed an acceptable fit to the data: CFI = 0.96, TLI = 0.94, RMSEA = 0.03, SRMR = 0.04.

## Structural Model

The structural model for Uganda is given below.

## lavaan 0.6-10 ended normally after 72 iterations
##
## Estimator DWLS
## Optimization method NLMINB
## Number of model parameters 56
##
## Number of observations 1054
##
## Model Test User Model:
## Standard Robust
## Test Statistic 188.761 245.802
## Degrees of freedom 95 95
## P-value (Chi-square) 0.000 0.000
## Scaling correction factor 0.831
## Shift parameter 18.606
## simple second-order correction
##
## Model Test Baseline Model:
##
## Test statistic 2523.700 1523.289
## Degrees of freedom 120 120
## P-value 0.000 0.000
## Scaling correction factor 1.713
##
## User Model versus Baseline Model:
##
## Comparative Fit Index (CFI) 0.961 0.893
## Tucker-Lewis Index (TLI) 0.951 0.864
##
## Robust Comparative Fit Index (CFI) NA
## Robust Tucker-Lewis Index (TLI) NA
##
## Root Mean Square Error of Approximation:
##
## RMSEA 0.031 0.039
## 90 Percent confidence interval - lower 0.024 0.033
## 90 Percent confidence interval - upper 0.037 0.045
## P-value RMSEA <= 0.05 1.000 0.999
##
## Robust RMSEA NA
## 90 Percent confidence interval - lower NA
## 90 Percent confidence interval - upper NA
##
## Standardized Root Mean Square Residual:
##
## SRMR 0.037 0.037
##
## Parameter Estimates:
##
## Standard errors Robust.sem
## Information Expected
## Information saturated (h1) model Unstructured
##
## Latent Variables:
## Estimate Std.Err z-value P(>|z|) Std.lv Std.all
## A =~
## C5_1_C5 1.000 0.760 0.672
## C5_2_C5 1.077 0.103 10.467 0.000 0.818 0.582
## C5_3_C5 0.652 0.059 11.099 0.000 0.496 0.517
## C5_4_C5 0.658 0.059 11.112 0.000 0.500 0.524
## B =~
## E9_3_E9 1.000 0.516 0.589
## E14_6_E14 1.011 0.091 11.168 0.000 0.521 0.585
## E14_7_E14 0.749 0.091 8.202 0.000 0.386 0.367
## E14_8_E14 0.961 0.091 10.531 0.000 0.495 0.467
## C =~
## E7_6_E7 1.000 0.805 0.608
## E7_5_E7 0.644 0.091 7.078 0.000 0.519 0.360
## E7_8_E7 0.764 0.101 7.567 0.000 0.615 0.441
## E7_10_E7 0.919 0.107 8.616 0.000 0.740 0.589
## D =~
## E8_2_E8 1.000 0.932 0.606
## E8_3_E8 1.066 0.176 6.063 0.000 0.994 0.671
## E8_4_E8 0.237 0.062 3.811 0.000 0.221 0.170
##
## Regressions:
## Estimate Std.Err z-value P(>|z|) Std.lv Std.all
## outcome ~
## A 0.061 0.067 0.917 0.359 0.046 0.046
## B 0.083 0.147 0.567 0.571 0.043 0.043
## C -0.482 0.121 -3.982 0.000 -0.388 -0.388
## D 0.052 0.072 0.721 0.471 0.049 0.049
##
## Covariances:
## Estimate Std.Err z-value P(>|z|) Std.lv Std.all
## A ~~
## B 0.108 0.020 5.304 0.000 0.277 0.277
## C -0.048 0.027 -1.767 0.077 -0.079 -0.079
## D -0.008 0.034 -0.229 0.819 -0.011 -0.011
## B ~~
## C -0.246 0.032 -7.613 0.000 -0.593 -0.593
## D -0.141 0.030 -4.736 0.000 -0.293 -0.293
## C ~~
## D 0.399 0.070 5.733 0.000 0.531 0.531
##
## Intercepts:
## Estimate Std.Err z-value P(>|z|) Std.lv Std.all
## .C5_1_C5 4.190 0.060 69.664 0.000 4.190 3.705
## .C5_2_C5 3.729 0.062 59.995 0.000 3.729 2.651
## .C5_3_C5 4.510 0.062 72.876 0.000 4.510 4.703
## .C5_4_C5 4.369 0.050 88.191 0.000 4.369 4.584
## .E9_3_E9 4.472 0.046 97.891 0.000 4.472 5.111
## .E14_6_E14 4.459 0.047 94.037 0.000 4.459 5.002
## .E14_7_E14 4.228 0.051 82.541 0.000 4.228 4.021
## .E14_8_E14 4.198 0.051 81.953 0.000 4.198 3.955
## .E7_6_E7 1.912 0.078 24.506 0.000 1.912 1.443
## .E7_5_E7 2.478 0.053 46.712 0.000 2.478 1.718
## .E7_8_E7 2.153 0.064 33.600 0.000 2.153 1.542
## .E7_10_E7 1.843 0.075 24.499 0.000 1.843 1.466
## .E8_2_E8 2.872 0.047 60.489 0.000 2.872 1.868
## .E8_3_E8 2.270 0.068 33.213 0.000 2.270 1.532
## .E8_4_E8 3.944 0.063 62.530 0.000 3.944 3.042
## .outcome 0.000 0.000 0.000
## A 0.000 0.000 0.000
## B 0.000 0.000 0.000
## C 0.000 0.000 0.000
## D 0.000 0.000 0.000
##
## Thresholds:
## Estimate Std.Err z-value P(>|z|) Std.lv Std.all
## outcome|t1 -0.265 0.039 -6.766 0.000 -0.265 -0.265
##
## Variances:
## Estimate Std.Err z-value P(>|z|) Std.lv Std.all
## .C5_1_C5 0.702 0.047 14.870 0.000 0.702 0.549
## .C5_2_C5 1.308 0.089 14.684 0.000 1.308 0.662
## .C5_3_C5 0.674 0.031 21.870 0.000 0.674 0.733
## .C5_4_C5 0.659 0.030 22.109 0.000 0.659 0.725
## .E9_3_E9 0.499 0.027 18.590 0.000 0.499 0.653
## .E14_6_E14 0.523 0.026 19.924 0.000 0.523 0.658
## .E14_7_E14 0.956 0.048 19.732 0.000 0.956 0.865
## .E14_8_E14 0.881 0.045 19.581 0.000 0.881 0.782
## .E7_6_E7 1.105 0.074 14.889 0.000 1.105 0.630
## .E7_5_E7 1.811 0.136 13.310 0.000 1.811 0.871
## .E7_8_E7 1.571 0.111 14.124 0.000 1.571 0.806
## .E7_10_E7 1.032 0.065 15.906 0.000 1.032 0.653
## .E8_2_E8 1.495 0.142 10.516 0.000 1.495 0.632
## .E8_3_E8 1.207 0.141 8.532 0.000 1.207 0.550
## .E8_4_E8 1.632 0.114 14.275 0.000 1.632 0.971
## .outcome 0.841 0.841 0.841
## A 0.577 0.073 7.913 0.000 1.000 1.000
## B 0.266 0.032 8.242 0.000 1.000 1.000
## C 0.649 0.115 5.620 0.000 1.000 1.000
## D 0.869 0.185 4.692 0.000 1.000 1.000
##
## Scales y*:
## Estimate Std.Err z-value P(>|z|) Std.lv Std.all
## outcome 1.000 1.000 1.000

## Summary

| **Factor** | **B (95% CI)** | **β (95% CI)** | **P-value** |
| --- | --- | --- | --- |
| Belief in religious protection (A) | 0.06 (-0.07, 0.19) | 0.05 (-0.05. 0.15) | 0.40 |
| Vaccination is a norm and worth doing (B) | 0.08 (-0.20, 0.37) | 0.04 (-0.11, 0.19) | 0.57 |
| Vaccination is practically difficult (C) | -0.48 (-0.72, -0.24) | -0.39 (-0.56, -0.21) | <0.001 |
| Poor service delivery experience (D) | 0.05 (-0.09. 0.19) | 0.05 (-0.08, 0.18) | 0.47 |

## Interpretation

The structural model is shown to be a good fit to the data (CFI = 0.96, TLI = 0.95, RMSEA = 0.03, SRMR = 0.04).

In the regression analysis, only Vaccination is practically difficult (Factor C) was observed to have an impact on the vaccination outcome, making it less likely (B = -0.48, $\beta$ = -0.39, p=0<0.001). The other factors had no observed effect on the outcome (Belief in religious protection: B = 0.061, $\beta$ = 0.046, p=0.92; Vaccination is a norm and worth doing: B = -0.083, $\beta$ = -0.043, p=0.57 Poor service delivery experience: B = 0.052, $\beta$ = 0.049, p=0.72).

# Guinea

## Factor analysis and Measurement Model

The factor analysis for Guinea produced 4 factors:

| Factor | Variables |
| --- | --- |
| Belief in religious protection (A) | My religious faith protects me and my family from harm (C5_1_C5), God is the only protection needed against harm (C5_3_C5), My religious faith guides decisions in my life (C5_4_C5) |
| Support for vaccination from others (B) | My spouse / partner helped/ ensured that my child was vaccinated (E14_3_E14), My mother/ mother-in-law helped/ ensured that my child was vaccinated (E14_4_E14), It is normal in this community to vaccinate your children (E14_6_E14), Religious leaders are supportive of vaccination (E14_7_E14), I trust that the government knows what is right for children (E14_8_E14) |
| Belief that vaccines are harmful (C) | Having many vaccinations at once is hard for children to bear (E6_3_E6), It is difficult for me to manage the side effects (fever, rash, pain) of vaccination (E6_7_E6), Vaccines are a way for global/western countries/organisations to control us (E6_9_E6) |
| Poor service delivery experience (D) | The staff in the hospital are rude to me (E8_2_E8), The clinic or hospital is dirty (E8_3_E8), The queues are too long at the clinic/ hospital where the vaccination takes place (E8_4_E8) |

The measurement model for these factors is given below.

## lavaan 0.6-10 ended normally after 56 iterations
##
## Estimator ML
## Optimization method NLMINB
## Number of model parameters 34
##
## Number of observations 1000
##
## Model Test User Model:
##
## Test statistic 236.831
## Degrees of freedom 71
## P-value (Chi-square) 0.000
##
## Model Test Baseline Model:
##
## Test statistic 2147.011
## Degrees of freedom 91
## P-value 0.000
##
## User Model versus Baseline Model:
##
## Comparative Fit Index (CFI) 0.919
## Tucker-Lewis Index (TLI) 0.897
##
## Loglikelihood and Information Criteria:
##
## Loglikelihood user model (H0) -19793.093
## Loglikelihood unrestricted model (H1) -19674.678
##
## Akaike (AIC) 39654.186
## Bayesian (BIC) 39821.050
## Sample-size adjusted Bayesian (BIC) 39713.064
##
## Root Mean Square Error of Approximation:
##
## RMSEA 0.048
## 90 Percent confidence interval - lower 0.042
## 90 Percent confidence interval - upper 0.055
## P-value RMSEA <= 0.05 0.645
##
## Standardized Root Mean Square Residual:
##
## SRMR 0.043
##
## Parameter Estimates:
##
## Standard errors Standard
## Information Expected
## Information saturated (h1) model Structured
##
## Latent Variables:
## Estimate Std.Err z-value P(>|z|) Std.lv Std.all
## A =~
## C5_1_C5 1.000 0.513 0.740
## C5_3_C5 0.684 0.050 13.603 0.000 0.351 0.661
## C5_4_C5 0.712 0.053 13.514 0.000 0.365 0.620
## B =~
## E14_3_E14 1.000 0.536 0.591
## E14_4_E14 1.287 0.105 12.242 0.000 0.690 0.573
## E14_6_E14 0.687 0.054 12.634 0.000 0.368 0.610
## E14_7_E14 0.815 0.082 9.967 0.000 0.437 0.424
## E14_8_E14 1.119 0.089 12.539 0.000 0.599 0.600
## C =~
## E6_3_E6 1.000 0.860 0.564
## E6_7_E6 1.282 0.133 9.674 0.000 1.102 0.642
## E6_9_E6 1.035 0.108 9.612 0.000 0.890 0.512
## D =~
## E8_2_E8 1.000 0.958 0.582
## E8_3_E8 1.176 0.143 8.230 0.000 1.127 0.683
## E8_4_E8 0.449 0.063 7.117 0.000 0.430 0.324
##
## Covariances:
## Estimate Std.Err z-value P(>|z|) Std.lv Std.all
## A ~~
## B 0.034 0.012 2.729 0.006 0.123 0.123
## C 0.028 0.021 1.338 0.181 0.064 0.064
## D -0.028 0.024 -1.164 0.244 -0.056 -0.056
## B ~~
## C -0.075 0.023 -3.206 0.001 -0.162 -0.162
## D -0.093 0.026 -3.507 0.000 -0.181 -0.181
## C ~~
## D 0.352 0.057 6.227 0.000 0.427 0.427
##
## Variances:
## Estimate Std.Err z-value P(>|z|) Std.lv Std.all
## .C5_1_C5 0.218 0.020 10.968 0.000 0.218 0.453
## .C5_3_C5 0.158 0.011 14.691 0.000 0.158 0.563
## .C5_4_C5 0.213 0.013 16.413 0.000 0.213 0.615
## .E14_3_E14 0.536 0.031 17.293 0.000 0.536 0.651
## .E14_4_E14 0.972 0.055 17.741 0.000 0.972 0.671
## .E14_6_E14 0.229 0.014 16.754 0.000 0.229 0.628
## .E14_7_E14 0.873 0.043 20.329 0.000 0.873 0.820
## .E14_8_E14 0.638 0.037 17.033 0.000 0.638 0.640
## .E6_3_E6 1.587 0.103 15.419 0.000 1.587 0.682
## .E6_7_E6 1.735 0.142 12.210 0.000 1.735 0.588
## .E6_9_E6 2.231 0.130 17.219 0.000 2.231 0.738
## .E8_2_E8 1.793 0.135 13.267 0.000 1.793 0.661
## .E8_3_E8 1.454 0.162 8.977 0.000 1.454 0.534
## .E8_4_E8 1.575 0.076 20.689 0.000 1.575 0.895
## A 0.263 0.026 10.181 0.000 1.000 1.000
## B 0.287 0.034 8.407 0.000 1.000 1.000
## C 0.740 0.107 6.944 0.000 1.000 1.000
## D 0.918 0.142 6.478 0.000 1.000 1.000

Summary

| **Factor** | **Variable** | **Factor loading** | **Standard error** | **P-value** | **Standardised factor loading** |
| --- | --- | --- | --- | --- | --- |
| Belief in religious protection (A) | My religious faith protects me and my family from harm | 1.00 |  |  | 0.74 |
|  | God is the only protection needed against harm | 0.68 | 0.05 | <0.001 | 0.66 |
|  | My religious faith guides decisions in my life | 0.71 | 0.05 | <0.001 | 0.62 |
| Support for vaccination from others (B) | My spouse / partner helped/ ensured that my child was vaccinated | 1.00 |  |  | 0.59 |
|  | My mother/ mother-in-law helped/ ensured that my child was vaccinated | 1.29 | 0.12 | <0.001 | 0.57 |
|  | It is normal in this community to vaccinate your children | 0.69 | 0.05 | <0.001 | 0.61 |
|  | Religious leaders are supportive of vaccination | 0.82 | 0.08 | <0.001 | 0.42 |
|  | I trust that the government knows what is right for children | 1.12 | 0.09 | <0.001 | 0.60 |
| Belief that vaccines are harmful (C) | Having many vaccinations at once is hard for children to bear | 1.00 |  |  | 0.56 |
|  | It is difficult for me to manage the side effects (fever, rash, pain) of vaccination | 1.28 | 0.13 | <0.001 | 0.64 |
|  | Vaccines are a way for global/western countries/organisations to control us | 1.04 | 0.12 | <0.001 | 0.51 |
| Poor service delivery experience (D) | The staff in the hospital are rude to me | 1.00 |  |  | 0.58 |
|  | The clinic or hospital is dirty | 1.18 | 0.14 | <0.001 | 0.68 |
|  | The queues are too long at the clinic/ hospital where the vaccination takes place | 0.45 | 0.06 | <0.001 | 0.32 |

The measurement model showed an acceptable fit to the data: CFI = 0.92, TLI = 0.90, RMSEA = 0.05, SRMR = 0.04.

## Structural Model

The structural model for Guinea is given below.

## lavaan 0.6-10 ended normally after 104 iterations
##
## Estimator DWLS
## Optimization method NLMINB
## Number of model parameters 53
##
## Number of observations 1000
##
## Model Test User Model:
## Standard Robust
## Test Statistic 204.478 244.668
## Degrees of freedom 81 81
## P-value (Chi-square) 0.000 0.000
## Scaling correction factor 0.910
## Shift parameter 20.006
## simple second-order correction
##
## Model Test Baseline Model:
##
## Test statistic 3013.336 1849.894
## Degrees of freedom 105 105
## P-value 0.000 0.000
## Scaling correction factor 1.667
##
## User Model versus Baseline Model:
##
## Comparative Fit Index (CFI) 0.958 0.906
## Tucker-Lewis Index (TLI) 0.945 0.878
##
## Robust Comparative Fit Index (CFI) NA
## Robust Tucker-Lewis Index (TLI) NA
##
## Root Mean Square Error of Approximation:
##
## RMSEA 0.039 0.045
## 90 Percent confidence interval - lower 0.032 0.039
## 90 Percent confidence interval - upper 0.046 0.051
## P-value RMSEA <= 0.05 0.997 0.896
##
## Robust RMSEA NA
## 90 Percent confidence interval - lower NA
## 90 Percent confidence interval - upper NA
##
## Standardized Root Mean Square Residual:
##
## SRMR 0.043 0.043
##
## Parameter Estimates:
##
## Standard errors Robust.sem
## Information Expected
## Information saturated (h1) model Unstructured
##
## Latent Variables:
## Estimate Std.Err z-value P(>|z|) Std.lv Std.all
## A =~
## C5_1_C5 1.000 0.510 0.734
## C5_3_C5 0.675 0.027 24.830 0.000 0.344 0.648
## C5_4_C5 0.735 0.029 24.942 0.000 0.375 0.636
## B =~
## E14_3_E14 1.000 0.517 0.570
## E14_4_E14 1.190 0.104 11.444 0.000 0.615 0.511
## E14_6_E14 0.757 0.052 14.415 0.000 0.391 0.648
## E14_7_E14 0.828 0.077 10.700 0.000 0.428 0.415
## E14_8_E14 1.177 0.088 13.447 0.000 0.608 0.609
## C =~
## E6_3_E6 1.000 0.799 0.524
## E6_7_E6 1.478 0.284 5.210 0.000 1.181 0.687
## E6_9_E6 1.114 0.208 5.348 0.000 0.890 0.512
## D =~
## E8_2_E8 1.000 0.943 0.573
## E8_3_E8 1.337 0.274 4.879 0.000 1.260 0.764
## E8_4_E8 0.390 0.095 4.097 0.000 0.368 0.277
##
## Regressions:
## Estimate Std.Err z-value P(>|z|) Std.lv Std.all
## outcome ~
## A 0.135 0.078 1.726 0.084 0.069 0.069
## B 0.462 0.081 5.675 0.000 0.239 0.239
## C -0.267 0.084 -3.178 0.001 -0.213 -0.213
## D 0.130 0.067 1.944 0.052 0.123 0.123
##
## Covariances:
## Estimate Std.Err z-value P(>|z|) Std.lv Std.all
## A ~~
## B 0.044 0.009 4.678 0.000 0.167 0.167
## C 0.029 0.022 1.296 0.195 0.070 0.070
## D -0.022 0.020 -1.073 0.283 -0.045 -0.045
## B ~~
## C -0.062 0.021 -2.923 0.003 -0.150 -0.150
## D -0.077 0.027 -2.837 0.005 -0.157 -0.157
## C ~~
## D 0.308 0.073 4.230 0.000 0.409 0.409
##
## Intercepts:
## Estimate Std.Err z-value P(>|z|) Std.lv Std.all
## .C5_1_C5 4.807 0.065 73.792 0.000 4.807 6.926
## .C5_3_C5 4.880 0.047 104.332 0.000 4.880 9.196
## .C5_4_C5 4.845 0.049 98.239 0.000 4.845 8.225
## .E14_3_E14 4.687 0.090 51.843 0.000 4.687 5.166
## .E14_4_E14 4.437 0.112 39.447 0.000 4.437 3.687
## .E14_6_E14 4.793 0.039 121.994 0.000 4.793 7.943
## .E14_7_E14 4.480 0.070 63.799 0.000 4.480 4.344
## .E14_8_E14 4.550 0.078 58.310 0.000 4.550 4.556
## .E6_3_E6 3.909 0.103 38.009 0.000 3.909 2.563
## .E6_7_E6 3.415 0.080 42.563 0.000 3.415 1.988
## .E6_9_E6 2.872 0.056 51.647 0.000 2.872 1.652
## .E8_2_E8 2.230 0.097 22.885 0.000 2.230 1.354
## .E8_3_E8 2.204 0.107 20.686 0.000 2.204 1.335
## .E8_4_E8 4.203 0.102 41.021 0.000 4.203 3.168
## .outcome 0.000 0.000 0.000
## A 0.000 0.000 0.000
## B 0.000 0.000 0.000
## C 0.000 0.000 0.000
## D 0.000 0.000 0.000
##
## Thresholds:
## Estimate Std.Err z-value P(>|z|) Std.lv Std.all
## outcome|t1 0.253 0.040 6.315 0.000 0.253 0.253
##
## Variances:
## Estimate Std.Err z-value P(>|z|) Std.lv Std.all
## .C5_1_C5 0.222 0.007 33.022 0.000 0.222 0.461
## .C5_3_C5 0.163 0.004 39.123 0.000 0.163 0.580
## .C5_4_C5 0.207 0.006 37.253 0.000 0.207 0.595
## .E14_3_E14 0.556 0.025 22.316 0.000 0.556 0.676
## .E14_4_E14 1.070 0.070 15.214 0.000 1.070 0.739
## .E14_6_E14 0.211 0.009 22.864 0.000 0.211 0.580
## .E14_7_E14 0.880 0.043 20.511 0.000 0.880 0.828
## .E14_8_E14 0.627 0.028 22.807 0.000 0.627 0.629
## .E6_3_E6 1.689 0.153 11.065 0.000 1.689 0.726
## .E6_7_E6 1.557 0.201 7.763 0.000 1.557 0.528
## .E6_9_E6 2.232 0.233 9.593 0.000 2.232 0.738
## .E8_2_E8 1.822 0.211 8.620 0.000 1.822 0.672
## .E8_3_E8 1.136 0.258 4.402 0.000 1.136 0.417
## .E8_4_E8 1.624 0.130 12.475 0.000 1.624 0.923
## .outcome 0.890 0.890 0.890
## A 0.260 0.018 14.305 0.000 1.000 1.000
## B 0.267 0.033 8.131 0.000 1.000 1.000
## C 0.638 0.167 3.831 0.000 1.000 1.000
## D 0.889 0.266 3.345 0.001 1.000 1.000
##
## Scales y*:
## Estimate Std.Err z-value P(>|z|) Std.lv Std.all
## outcome 1.000 1.000 1.000

## Summary

| **Factor** | **B (95% CI)** | **β (95% CI)** | **P-value** |
| --- | --- | --- | --- |
| Belief in religious protection (A) | 0.14 (-0.02, 0.29) | 0.07 (-0.01, 0.15) | 0.08 |
| Support for vaccination from others (B) | 0.46 (0.30, 0.62) | 0.24 (0.16, 0.32) | <0.001 |
| Belief that vaccines are harmful (C) | -0.27 (-0.43, 0.10) | -0.21 (-0.33, -0.09) | 0.001 |
| Poor service delivery experience (D) | 0.13 (0.00, 0.26) | 0.12 (0.01, 0.24) | 0.05 |

##

## Interpretation

The structural model is shown to be a good fit to the data (CFI = 0.96, TLI = 0.95, RMSEA = 0.04, SRMR = 0.04).

In the regression analysis, there was some evidence that Belief in religious protection (Factor A) is observed to increase the probability of full vaccination (B = 0.135, $\beta$ = -0.069, p= 0.08). Support for vaccination from others (Factor B) was observed to increase the probability of vaccination (B = 0.462, $\beta$ = 0.239, p<0.001). Belief that vaccines are harmful (Factor C) was observed to decrease the probability of vaccination (B = -0.267, $\beta$ = -0.213, p= 0.001) and Poor service delivery experience (Factor D) was observed to increase it (B = 0.130, $\beta$ = 0.123, p=0.05)
